# Design, Synthesis and Biological Evaluation of 3-Hydrazonoindolin-2-one Derivatives as Novel HIV-1 RNase H Inhibitors

**DOI:** 10.3390/molecules30091868

**Published:** 2025-04-22

**Authors:** Yiying Zhang, Rao Wang, Yueyue Bu, Angela Corona, Laura Dettori, Enzo Tramontano, Christophe Pannecouque, Erik De Clercq, Shuai Wang, Ge Meng, Fen-Er Chen

**Affiliations:** 1 Engineering Center of Catalysis and Synthesis for Chiral Molecules, Department of Chemistry, Fudan University, Shanghai 200433, China; 22210220066@m.fudan.edu.cn (Y.Z.); shuaiwang@fudan.edu.cn (S.W.); 2 Shanghai Engineering Center of Industrial Asymmetric Catalysis for Chiral Drugs, Shanghai 200433, China; 3 Henan Key Laboratory of Nanomedicine for Targeting Diagnosis and Treatment, School of Pharmaceutical Sciences, Zhengzhou University, Zhengzhou 450001, China; wr20012022@163.com (R.W.); byy11292023@163.com (Y.B.); 4 Department of Life and Environmental Sciences, University of Cagliari, 09042 Monserrato, Italy; angela.corona@unica.it (A.C.); laura.dettori@unica.it (L.D.); tramon@unica.it (E.T.); 5 National Ph.D. Programme in One Health Approaches to Infectious Diseases and Life Science Research, Department of Public Health, Experimental and Forensic Medicine, University of Pavia, 27100 Pavia, Italy; 6 Rega Institute for Meical Research, KU Leuven, Herestraat 49, B-3000 Leuven, Belgium; christophe.pannecouque@kuleuven.be (C.P.); erik.declercq@kuleuven.be (E.D.C.); 7 College of Tea (Pu’er), West Yunnan University of Applied Sciences, Pu’er 665000, China

**Keywords:** AIDS, HIV, 3-hydrazonoindolin-2-one, RNase H

## Abstract

Targeting ribonuclease H (RNase H) has emerged as a highly promising strategy for treating HIV-1. In this study, a series of novel 3-hydrazonoindolin-2-one derivatives were designed and synthesized as potential inhibitors of HIV-1 RNase H. Notably, several of these derivatives displayed micromolar inhibitory activity. Among the compounds examined, the hit compound demonstrated potent inhibition of HIV-1 RNase H, boasting a Ki value of 2.31 μM. Additionally, the most potent compound of this general structure exhibited remarkable inhibitory activity, with Ki values of 0.55 μM. Through docking studies, the key interactions of this ligand within the active site of RNase H were uncovered. This novel chemical structure can be regarded as a prospective scaffold for the future development of RNase H inhibitors.

## 1. Introduction

Acquired Immunodeficiency Syndrome (AIDS) is primarily caused by the Human Immunodeficiency Virus (HIV), which remains a significant global public health concern, with ongoing transmission observed in all countries worldwide [1,2]. By the end of 2023, it was estimated that 39.9 million people were living with HIV [3]. In 2023, 1.3 million people were newly infected with HIV, and 630,000 people died from HIV-related causes [3]. Consequently, the necessity for prevention and treatment of HIV remains critical. At present, active antiretroviral therapy (ART) represents the most efficacious treatment for the prevention of the progression of AIDS and has transformed HIV infection from a fatal disease to a chronic one [4,5]. Nevertheless, the efficacy of HAART may be diminished as a consequence of the emergence of drug-resistant mutants of HIV-1 [6]. Therefore, there is still a great need for novel anti-HIV-1 medications to tackle the issues of drug resistance.

Reverse transcriptase (RT) is the most commonly targeted protein encoded by HIV, with over half of the approved anti-HIV-1 therapeutics designed to inhibit its function. Structurally, RT consists of two distinct domains with specialized enzymatic activities: the polymerase (pol) domain and the RNase H domain [7,8,9]. Currently, the primary class of reverse transcriptase inhibitors in clinical use includes both nucleoside and non-nucleoside inhibitors, which specifically target the polymerase activity of RT [10,11]. In contrast, no inhibitors targeting the RNase H domain of RT have progressed to clinical development to date [12].

**Figure 1:**
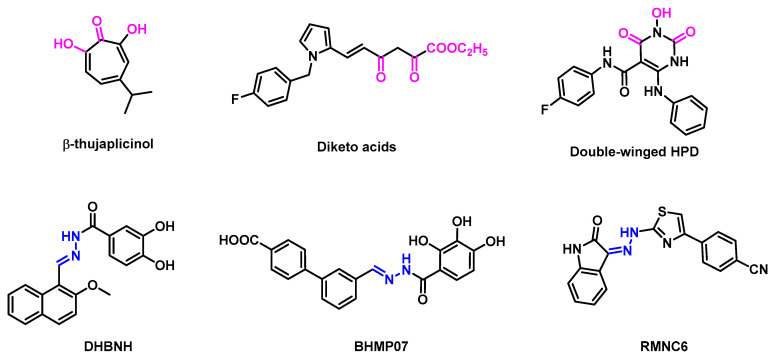
Chemical structures of some representative RNHIs. The pink areas indicate the three-oxygen pharmacophore of the RNHIs. The blue areas show the hydrazone moiety.

The reported RNase H inhibitors are primarily classified into two categories: active site inhibitors and allosteric inhibitors [6,13] (Figure 1). RNase H active site inhibitors usually contain trioxo-chelating pharmacophores or their equivalent structures [14]. Those inhibitors targeting the active site interact with four conserved amino acid residues, abbreviated as DEDD (D443, E478, D498, and D549) motifs, by chelating with two magnesium ions in the active site, thus interfering with the synergistic interaction between the DEDD sequences and the two metal ions, resulting in a drastic decrease in the hydrolytic activity of RNase H or even inactivation [15,16]. However, the current inhibitory effects observed at the enzyme level have hardly been translated into a significant antiviral activity in cell culture [17]. Encouragingly, the antiviral activity against HIV-1 was observed with the dual-wing HPD (3-hydroxypyrimidine-2,4-diones) subtype [18]. Interestingly, it was discovered that some hydrazone derivatives could inhibit HIV-1 RNase H function by acting as a dual inhibitor of RNase H and DNA polymerase. DHBNH was the first allosteric RNHI to be identified [19]. Based on the DHBNH, RMNC6 was identified through virtual screening as a dual inhibitor, which interacted near the active site [20].

## 2. Results and Discussion

### 2.1. Design of New Compounds

**Figure 2:**
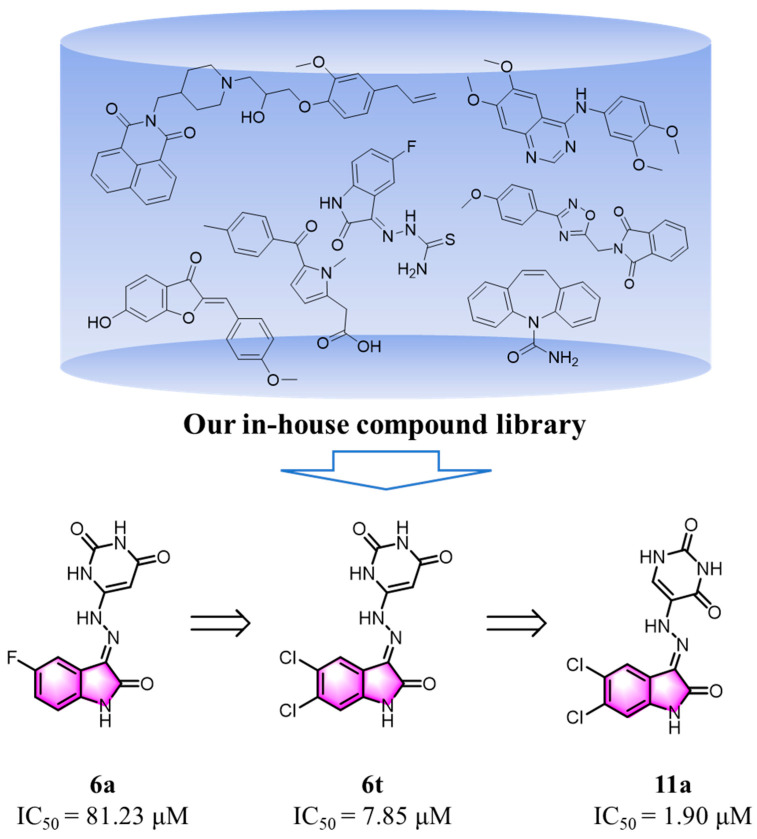
The discovery of novel 3-hydrazonoindolin-2-one derivatives.

Our research journey commenced with a systematic screening of our internal compound library of approximately 9900 compounds. This screening effort led to the discovery of the first 3-hydrazonoindolin-2-one-based RNase H inhibitor **6a** (IC_50_ = 81.23 μM) (Figure 2). Following this initial finding, 22 novel 3-hydrazonoindolin-2-one analogs were synthesized and evaluated for their inhibitory activity. Through an iterative optimization process, we developed compound **6t**, which had significantly enhanced potency, with an IC_50_ value of 7.85 μM. This represented an approximate 10-fold improvement compared to compound **6a**. To thoroughly explore the structure–activity relationship (SAR) of this chemical framework, we carried out further extensive modifications. This ultimately led to the identification of two more analogs: compounds **11a** and **11b**. These two compounds displayed strong inhibitory activity against RNase H. Specifically, compound **11a** had an IC_50_ value of 1.90 μM, and compound **11b** had an IC_50_ value of 2.20 μM. Compared to the initial lead compound **6a**, this was roughly a 40-fold increase in potency.

### 2.2. Chemistry

**Scheme 1:**
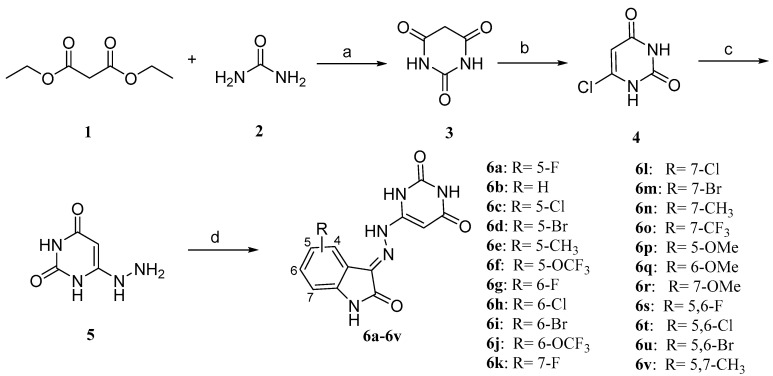
General synthetic route for target compounds. (a) Sodium methoxide, methanol, reflux, 10 h, yield 90%; (b) (1) POCl_3_, DMF, 130 °C, 4 h, yield 90%; (2) 10% NaOH, Water, Reflux, 2 h, yield 99%; (c) 50% N_2_H_4_·H_2_O, Water, Reflux, 2 h, yield 95%; (d) Indoline-2,3-dione, Ethanol, drops of glacial acetic acid, 80 °C, 2 h, yield 58–73%.

A muti-step synthetic approach was used to synthesize the novel 3-hydrazonoindolin-2-one compounds **6a**–**6v** (Scheme 1). Briefly, the barbituric acid (**3**) was prepared by the condensation reaction of diethyl malonate with urea catalyzed by sodium methanol. Then, 6-chloropyrimidine-2,4(1*H*,3*H*)-dione (**4**) was prepared according to a procedure reported by White and Hansen [21] and afterward was hydrolyzed. The key intermediate 6-hydrazineylpyrimidine-2,4(1*H*,3*H*)-dione (**5**) was synthesized by refluxing a mixture of 50% hydrazine hydrate (2.5 eqv.) and the compound 4 in anhydrous ethanol. Afterward, 6-hydrazineylpyrimidine-2,4(1*H*,3*H*)-dione (**5**) was condensed with the suitable substituted isatin in the presence of catalytic glacial acetic acid in absolute ethanol at refluxing for 2 h. The target compounds 6a-6v were isolated in the good yields ranging from 58% to 73% as a yellow solid.

**Scheme 2:**
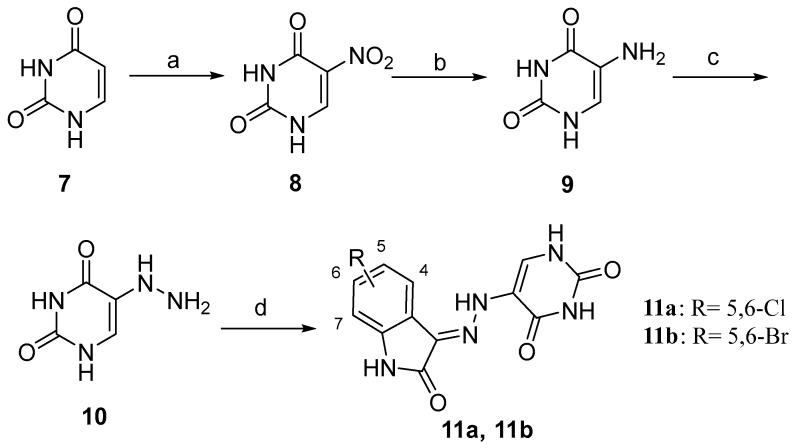
General synthetic route for target compounds. (a) Nitric acid, sulfuric acid, 50 °C, 3 h, yield 90%; (b) 25% ammonium hydroxide, Na_2_S_2_O_2_, water, 80 °C, 4 h, yield 91%; (c) (1) NaNO_2_, 36% hydrochloric acid, water, 0 °C, 2 h; (2) stannous chloride, 0 °C, 2 h, yield 90%; (d) indoline-2,3-dione, ethanol, drops of glacial acetic acid, 80 °C, 2 h, yield 70–83%.

The synthesis of compounds **11a** and **11b** was performed as reported in Scheme 2. The commercially available uracil (**7**) is nitrated in a 1:3 system of concentrated nitric and sulphuric acids to afford 5-nitropyrimidine-2,4(1*H*,3*H*)-dione (**8**). Subsequently, the compound **8** proceeded through a reduction reaction, resulting in the formation of 5-aminopyrimidine-2,4(1*H*,3*H*)-dione (**9**). This was then reduced to the 5-hydrazinepyrimidine-2,4(1*H*,3*H*)-dione (**10**), the pivotal intermediate **10**, via the Sandmeyer reaction. Afterward, 5-hydrazineylpyrimidine-2,4(1*H*,3*H*)-dione (**10**) was condensed with the suitable substituted isatin in the presence of catalytic glacial acetic acid in absolute ethanol at refluxing for 2 h. The target compounds **11a** and **11b** were isolated in good yields ranging from 70% to 83% as a yellow solid.

### 2.3. Evaluation of Biological Activities

A total of 24 functionalized 3-hydrazonoindolin-2-one derivatives were evaluated against HIV-1 RNase H (Table 1). Most of the compounds showed effective inhibitory activity against HIV-1 RNase H with the exception of **6b**, **6d**, **6e**, **6h**, **6k** and **6p**–**6r**. The results confirmed that the pyrimidine-2,4(1*H*,3*H*)-dione scaffold was particularly efficacious in achieving inhibition. Moderate inhibitory activities were observed for most 7-subsitituted compounds: **6n** (IC_50_ = 25.49 ± 1.37 μM), **6o** (IC_50_ = 24.06 ± 7.02 μM), **6v** (IC_50_ = 23.58 ± 3.89 μM) and 6-subsitituted compounds: **6i** (IC_50_ = 23.59 ± 1.11 μM). In particular, the compounds **6f** (IC_50_ = 8.94 ± 1.43 μM), **6j** (IC_50_ = 15.28 ± 0.010 μM), **6t** (IC_50_ = 7.85 ± 0.55 μM) and **6u** (IC_50_ = 11.83 ± 0.81 μM), which carried the trifluoromethoxy group at the C-5 or C-6 of the phenylene ring and halogen di-substitution at both the C-5 and C-6 positions, exhibited IC_50_ values in the low micromolar range. Introducing the methoxy group on the isatin phenyl ring (**6p**–**6r**) resulted in a complete loss of activity, in contrast to the few examples of compounds bearing an *O*-methyl group on the phenyl rings, which were previously reported to be active [22].

Then, an attempt was made to alter the position of pyrimidine-2,4(1*H*,3*H*)-dione in order to ascertain whether a more promising active molecule could be obtained. It was encouraging to note that compounds **11a** and **11b** exhibited notable activity, with IC_50_ values of 1.90 μM and 2.20 μM, respectively. These values were 4-fold and 40-fold more potent than those observed for compounds **6t** (IC_50_ = 7.85 ± 0.55 μM) and **6a** (IC_50_ = 81.23 ± 11.36 μM), respectively.

The newly synthesized compounds **6a**, **11a**, and **11b** were evaluated for their anti-HIV-1 (strain III_B_) activity in MT-4 cells, with their cytotoxicity assessed in parallel, as summarized in Table 2. Analysis of the activity data revealed that three compounds exhibited antiviral activity at the cellular level, with **11a** demonstrating the most potent anti-HIV-1 effect, achieving an SI of 10. These results confirm the validity of the isatin pyrimidine-2,4(1*H*,3*H*)-dione hydrazone scaffold and highlight its potential for further structural optimization and investigation.

### 2.4. Molecular Modeling

**Figure 3:**
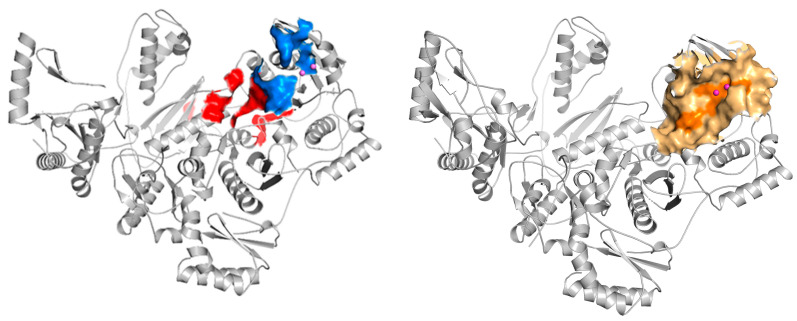
The binding site of BHMP07 (blue domain), the binding site of RMNC6 (red domain) and the designed binding site (orange domain).

According to the available literature, in the case of hydrazone/hydrazine RNase H inhibitors, the binding pocket has been predicted to occur at the interface between the DNA:RNA substrate and the RNase H domain, in the vicinity of residue Q500 [23]. This residue has been proven to interact with the RNA strand in the RNA:DNA double-stranded substrate, which has drawn attention to the search for some novel druggable sites for RNase H inhibition [24,25]. Solution NMR experiments [26] and docking results for BHMP07 demonstrated that residues near BHMP07 included D443, G444, A445, S499, Q500 and Y501 (Figure 3: blue domain). Another result of mutagenesis studies [20] demonstrated that the residues within the RT RNase H domain, specifically N474, Y501, A502 and A508, played a pivotal role in the inhibition of the endonuclease activity of RT by the inhibitor RMNC6 (Figure 3: red domain). Therefore, we defined the binding site as an 8 Å grid centered on key residues, including the highly conserved key residues D443, E478, D498, and D549, which chelates Mg^2+^ and Q500 of the p66 subunit of HIV-1 RT (Figure 3: orange domain).

**Figure 4:**
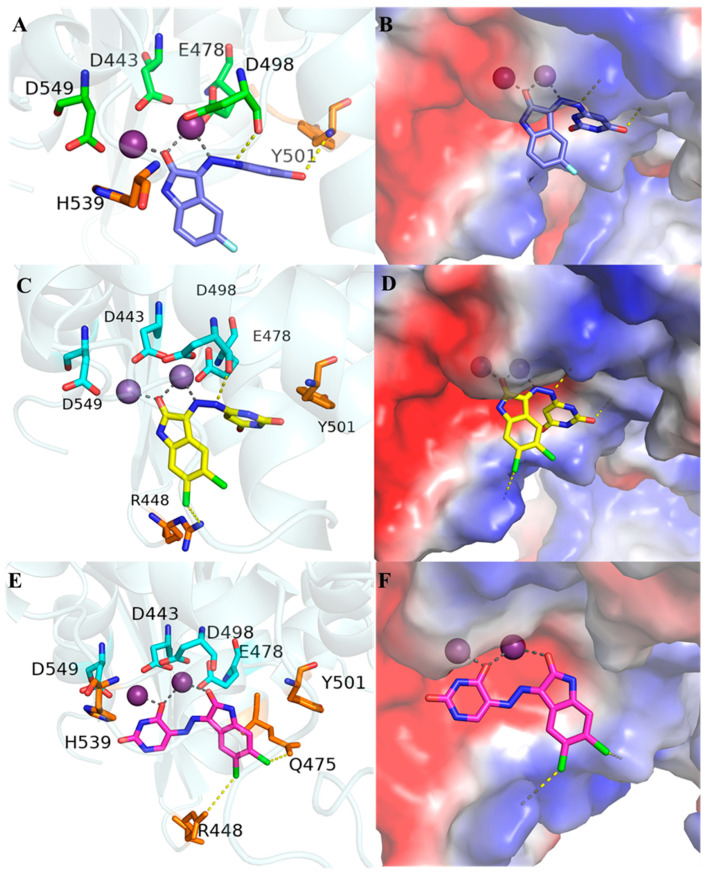
(**A**,**B**) Binding mode of compound **6a** (blue sticks), (**C**,**D**) **6t** (yellow sticks), and (**E**,**F**) **11a** (purple sticks) with RNase H (PDB: 3QIP). The highly conserved DEDD residues are shown as greensticks and cyan sticks and other interacting residues are illustrated as orange sticks. The metal ions Mg^2+^ are displayed as purple balls. The dotted yellow line indicates the interactions between ligands and residues, while the grey line indicates the metal coordination.

To clarify the reasons behind the inhibitory activity displayed by the novel compounds, the molecular docking study of compounds **6a**, **6t** and **11a** with the RNase H active site were performed using a model generated from the PDB code 3QIP [27]) (Figure 4). The modeling indicated that in the case of **6t** (Figure 4C,D), the oxygen atom of the ketone on isatin and the NH group on hydrazine will form a tightly chelated complex with the Mg^2+^ atom in the active site, together with the highly conserved residues DEDD (D443, E549, D498, and D478). In regard to **11a** (Figure 4E,F), the oxygen atom of the ketone on isatin and the oxygen atom on uracil exhibited this same behavior. The binding geometry was consistent with the literature that isatin-derived Schiff bases can act as ligands to form stable coordination compounds with a range of transition metal ions [28,29,30,31] and also was in accordance with our previous work [32]. It was noteworthy that the major binding group was localized in a highly polar environment within the binding site, which contained residues D443, E478, D498, D549 and H539, in addition to two water molecules involved in chelating one of the Mg^2+^ ions.

A detailed comparison of the compounds **6a** and **6t** (Figure 4A–D) reveals that the introduction of two chlorine atoms in **6t** significantly enhances its binding interactions with the target protein. Specifically, one of the chlorine atoms in **6t** forms a strong and directional halogen bond with the arginine residue R448. Halogen bonding, a highly favorable non-covalent interaction, contributes to the stabilization of the ligand–protein complex by providing additional binding energy and improving the orientation of the ligand within the binding pocket. This result explains the improved inhibitory potency for **6t** compared to **6a**.

In contrast, compound **11a** exhibits even greater binding stabilization due to the strategic positioning of its two chlorine atoms, both of which form halogen bonds with the R448 and Q475. It is worth noting that both Q475 and R448 are amino acidic residues that are highly conserved (Q475 variability lower than 0.5% and R448 exhibiting a degree of conservation of 96.70% among treated and naïve patients) [33] with Q475 being part of the HIV-1 RT RNase H primer grip motif [34]. The dual-halogen-bonding interactions in **11a** not only enhance the overall binding energy but also improve shape complementarity by filling hydrophobic cavities within the binding pocket. Additionally, the increased rigidity conferred by the chlorine atoms reduces the conformational flexibility of the ligand, further stabilizing the ligand–protein complex. These combined effects: halogen bonding and improved shape complementarity explain the superior binding performance of **11a** compared to **6t**, as evidenced by the structural analyses presented in Figure 4C–F.

The di-halogen substitution in compounds **6s**, **6t**, **6u**, **11a**, and **11b** significantly influences their biological activity through halogen bonding. Chlorine atoms, as seen in **6t** (IC_50_ = 7.85 μM) and **11a** (IC_50_ = 1.90 μM), form strong halogen bonds with key protein residues (e.g., R448 and Q475). This is exemplified by the improved activity of chlorine-substituted SSRIs (Selective Serotonin Reuptake Inhibitors), such as fluoxetine [35], where halogen bonding plays a critical role. In contrast, bromine atoms in **6u** (IC_50_ = 11.83 μM) and **11b** (IC_50_ = 2.20 μM) exhibit a more pronounced σ-hole, potentially leading to stronger halogen bonds than chlorine. However, their larger atomic radius introduces steric hindrance, which may compromise the complementarity with the protein binding pocket, resulting in slightly lower activity compared to chlorine-substituted analogs. Fluorine, as in **6s** (IC_50_ = 64.90 μM), is unable to form an effective halogen bond due to its high electronegativity and small size, and it also fails to contribute significantly to hydrophobic interactions or spatial filling, leading to the lowest activity among the series. Additionally, the moderate size of chlorine atoms allows them to fit well within hydrophobic pockets through van der Waals interactions without causing significant conformational distortion, as demonstrated in FGFR1 kinase inhibitors, where dichloro substitution enhanced activity by over 12,000-fold [35]. In contrast, the bulkier bromine atoms may introduce steric clashes, partially offsetting the benefits of halogen bonding. These findings highlight the delicate balance between electronic, steric, and hydrophobic effects in optimizing ligand–protein interactions.

### 2.5. Evaluation of the Mode of Action

**Figure 5:**
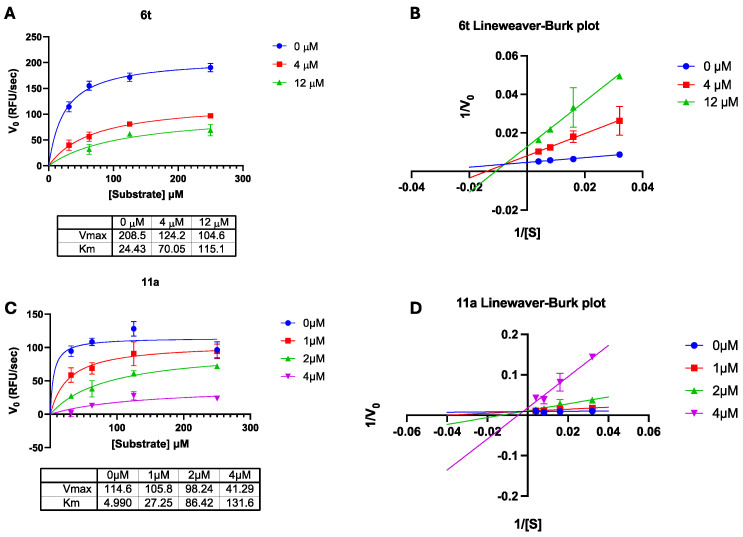
(**A**,**B**) Compound **6t** competition experiment: (**A**) Michaelis Menten and (**B**) Linewaver–Burk plot both display a pattern typical of a mixed model inhibitor. (**C**,**D**) Compound **11a** competition experiment: (**C**) Michaelis Menten and (**D**) Linewaver–Burk plot both display a pattern typical of a mixed model inhibitor.

Based on the docking binding predictions, compound **6t** and compound **11a** were chosen for further characterization. To study the interaction between the chemical compounds and the enzyme in complex with the substrate, we evaluated the competition of compounds **6a** and **11a** with the RNA substrate (Figure 5). The results showed a mixed model of competition for both compounds **6t** (Figure 5A,B) and compound **11a** (Figure 5C,D). The Ki values of both compounds were calculated, yielding a Ki value of 2.31 μM for compound **6t**, while compound **11a** showed a Ki value of 0.55 μM. Given the results obtained, it is hypothesized that the binding between the inhibitor and the RT protein may modulate the interaction between the enzyme and the RNA substrate, resulting in an increased apparent K_M_ and decreased apparent V_max_ when increasing the concentration of the inhibitor.

**Figure 6:**
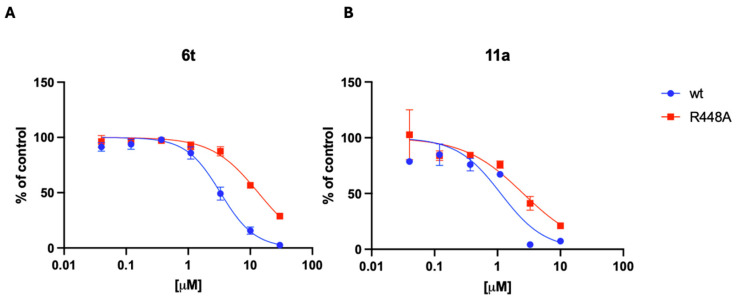
Dose–response curve of the inhibition of HIV-1 RT-associate RNase H activity curve with HIV-1 RT wt (blue) and HIV-1 RT R448A (red) (**A**) **6t** compound (**B**) **11a** compound.

Furthermore, to validate the docking binding prediction of the compounds in the active site of the RT, and the interaction with amino acid residue R448, which was predicted to stabilize the binding of the compounds through a hydrogen bond, we evaluated the activity of compounds **6t** and **11a** in parallel against the RT wt enzyme and the HIV-1 RT R448A mutant (Figure 6). Compound **6t** showed a marked four-fold shift of the inhibition curve, indicating a loss of potency of inhibition, which was a less pronounced two-fold shift for compound **11a.** The results confirmed the importance of residue R448 in the binding of inhibitors and validated the predicted binding site to the enzyme; indeed, the less pronounced effect of R448A mutation on **11a** inhibitory activity might be explained with the additional hydrogen bound predicted to form between **11a** and amino acid Q475 (Figure 6).

## 3. Conclusions

A novel series of compounds based on 3-hydrazonoindolin-2-one and uracil scaffold has been developed in this study. The most intriguing compounds were **6t** and **11a**. Of these, compound **11a** demonstrated the greatest potency as an inhibitor of RNase H functions, with an IC_50_ value of 1.90 μM and a Ki value of 0.55 μM. Docking studies suggested that the Schiff base moiety of the 3-hydrazonoindolin-2-one derivatives could bind to the active site of the RNase H domain and form a halogen bond with R448, which was widely favored as a ligand precursor owing to its coordination and stabilization [36]. Furthermore, competition studies and mutagenesis studies were carried out to confirm the binding action. Building upon the current findings, future studies will focus on the rational design of analogs with enhanced enzymatic activity, guided by the structure–activity relationships established in this work. Additionally, a comprehensive evaluation with cell-based antiviral assays will be prioritized to elucidate their therapeutic potential and cytotoxicity profiles. These efforts will integrate in vitro enzymatic data with cellular efficacy and safety assessments, ultimately bridging the gap between molecular-level activity and biological relevance. Though many different and promising chemical series of HIV-1 RNase H active site inhibitors have been discovered so far, few of these compounds are observed to exhibit antiviral activity in cell culture systems. In 2017, Vernekar et al. [18] found that an ultrapotent RNase H biochemical inhibitor (at under 0.08 micromolar) would compete against a much larger RNA:DNA duplex substrate, effectively resulting in the achievement of a moderate level of antiviral activity (under 20 micromolar). Despite the continuing absence of potent and selective HIV-1 RNase H inhibitors for the treatment of HIV-1 infections, further investigations into these compounds remain worthwhile in order to achieve an alternative method for anti-HIV treatment.

## 4. Experiment

### 4.1. Chemistry

The reagents and solvents utilized in this study were obtained from commercial suppliers and used as received, without further purification. Thin-layer chromatography (TLC) analysis was performed using a silica gel plate with a thickness of 0.25 mm. The separation results were subsequently visualized under ultraviolet (UV) light at a wavelength of 254 nm. The ^1^H NMR and ^13^C NMR spectra were recorded using a Bruker AV-400 spectrometer (Billerica, MA, USA), with samples dissolved in DMSO-*d*_6_ or CDCl_3_ and triflouroacetic acid-d. Tetramethylsilane (TMS) was employed as the internal standard for spectral characterization. Owing to the limited solubility of certain compounds, it was necessary to carefully select deuterated solvents that offered relatively higher solubility compatibility for each specific compound. Melting points were determined using a Hanon MP-430 digital melting point instrument (Jinan, China). High-resolution mass spectra (HRMSs) were acquired using a Waters Xevo G2-XS instrument (Milford, MA, USA). Analysis of sample purity was performed on an Agilent 1260 Infinity II (Santa Clara, CA, USA) using an Eclipse plus, 1.8 μm C18 column (50 mm × 2.1 mm).

#### 4.1.1. General Procedure for Preparation of Compound **3**

Diethyl malonate (**1**, 7.60 mL, 50 mmol) was added to a solution of sodium ethoxide (3.40 g, 50 mmol) in absolute ethanol (20 mL). Then, urea (**2**, 3.00 g, 50 mmol) was added to the resultant suspension of white precipitate, and the mixture was heated at reflux for 10 h. The reaction mixture was cooled to room temperature. The solvent was evaporated under reduced pressure, the solid residue was dissolved in water (50 mL), and the solution was acidified with hydrochloric acid (12 M) to pH = 2–3; then, the reaction mixture was placed in a refrigerator overnight and filtered, and the product was washed with cold water and dried at 45 °C to give the product of barbituric acid (**3**, 5.72 g, 44.66 mmol, 90%) as a white powder.

#### 4.1.2. General Procedure for Preparation of Compound **4**

Barbituric acid (**3**, 3.0 g, 23.42 mmol) and *N*,*N*-Dimethylaniline (296.89 μL, 2.34 mmol) were added in a flask. Under an ice bath, POCl_3_ (5.46 mL, 58.55 mL) was added to the suspension dropwise slowly. The temperature of the mixture was increased slowly to 100 °C for 2.5 h before being cooled to room temperature. Then, H_2_O was added carefully to hydrolyze the excess of POCl_3_. The crude product 2,4,6-trichloropyrimidine was filtered and dried and used for the next synthesis without further purification. To a solution of sodium hydroxide (4.69 g, 117.22 mmol) and water (40 mL), 2,4,6-trichloropyrimidine was added. The reaction mixture was heated at reflux for 2 h. The solution was cooled. While cooling in an ice bath, the pH was adjusted with concentrated hydrochloric acid to pH = 2. The resulting precipitate was separated by filtration. The resulting precipitate was washed with hot water. The resulting precipitate was dried to afford 6-chloropyrimidine-2,4(1*H*,3*H*)-dione (**4**, 3.42 g, 99%) as a white powder.

#### 4.1.3. General Procedure for Preparation of Compound **5**

Fifty percent hydrazine hydrate (3.20 mL, 51.18 mmol) was added to a solution of 6-chloropyrimidine-2,4(1*H*,3*H*)-dione (**4**, 3.00 g, 20.47 mmol) in absolute ethanol (25 mL). The mixture was heated at reflux for 3 h. The reaction was cooled to room temperature, and then the precipitate was filtered out. The precipitate was washed with dry ethanol (10 mL) three times and then dried to afford 6-hydrazineylpyrimidine-2,4(1*H*,3*H*)-dione (**5**, 2.79 g, 19.62 mmol, 95%) as a white powder.

#### 4.1.4. General Procedure for Preparation of Compounds **6a**–**6v** and **11a**–**11b**

The compounds were prepared from 6-hydrazineylpyrimidine-2,4(1*H*,3*H*)-dione (**5**, 0.50 mmol) and isatin (0.55 mmol) in dry ethanol with drops of glacial acetic acid refluxing for 2 h. After cooling to room temperature, the precipitate was filtered out and then purified through column chromatography with a mixture of DCM/TFA (from 100:1 to 25:1) as eluent. Or the precipitate could also be purified by recrystallization from TFA and petroleum ether to afford the products of **6a**–**6v** (yield 58–73%) as a yellow powder.

#### 4.1.5. General Procedure for Preparation of Compound **8**

A nitric acid solution (1.50 mL, 33.33 mmol, 70% solution) was added dropwise to a 25 mL round-bottomed flask containing concentrated sulphuric acid (5.47 mL, 100 mmol, 98% solution) under an ice-water bath. Pyrimidine-2,4(1*H*,3*H*)-dione (**7**, 2.00 g, 16.67 mmol) was added in several portions to the stirred solution in an ice bath. The reaction was heated to 55 °C for 3 h, cooled to 0 °C, and quenched with iced water (10 mL). The resulting white precipitate was collected by filtration, washed with a small amount of ice water and dried under reduced pressure to give 5-nitrobarbituric acid (**8**, 1.60 g, yield: 58%) as a white solid.

#### 4.1.6. General Procedure for Preparation of Compound **9**

A 50 mL round-bottom flask was charged with water (10 mL), 25% ammonia (6.50 mL) and 5-nitropyrimidine-2,4(1*H*,3*H*)-dione (**8**, 1.00 g, 6.38 mmol). Subsequently, Na_2_S_2_O_4_ (4.45 g, 25.55 mmol) was added in several portions, and the pH of the solution was adjusted to 8 with 25% ammonia. The resulting solution was stirred at 70 °C for three hours. The reaction mixture was then cooled to 0 °C using an ice/water bath, after which the solid was collected by filtration, yielding 0.68 mg (84%) of 5-aminopyrimidine-2,4(1*H*,3*H*)-dione as a white solid.

#### 4.1.7. General Procedure for Preparation of Compound **10**

5-aminopyrimidine-2,4(1*H*,3*H*)-dione (**9**, 1.00 g, 7.87 mmol) was dissolved in 6 mL of 18% hydrochloric acid in an ice bath. A solution of NaNO_2_ (0.54 g, 7.87 mmol) in 6 mL water was added dropwise to the reaction mixture. The reaction mixture was stirred at 0 °C for one hour in order to obtain a clear solution. A solution of SnCl_2_ (2.13 g, 9.44 mmol) in 6 mL of concentrated hydrochloric acid was added dropwise at 0 °C. The mixture was stirred at room temperature for two hours. The precipitate was filtered and washed with a small amount of 0.2 M sodium hydroxide solution and dried to give 5-hydrazineylpyrimidine-2,4(1*H*,3*H*)-dione(**10*,*** 1.02 g, yield: 91.22%) as a white solid.

*6-(2-(5-Fluoro-2-oxoindolin-3-ylidene)hydrazineyl)pyrimidine-2,4(1H,3H)-dione* (**6a**). Yellow solid, yield: 67%.^1^H NMR (400 MHz, DMSO-*d*_6_) *δ*: 12.30 (s, 1H, Isatin-NH), 11.26 (s, 1H, Pyrimidine-NH), 11.15 (s, 1H, Pyrimidine-NH), 10.74 (s, 1H, NH), 7.63 (dd, *J* = 8.3, 2.7 Hz, 1H, PhH), 7.22–7.08 (m, 1H, PhH), 6.90 (dd, *J* = 8.6, 4.2 Hz, 1H, PhH), 5.53 (s, 1H,CH).^13^C NMR (101 MHz, DMSO-*d*_6_) *δ*: 160.72, 158.69, 158.30, 150.43, 149.05, 145.58, 140.82, 129.86 (d, *J* = 8.9 Hz), 128.79 (d, *J* = 5.1 Hz), 121.67 (d, *J* = 25.1 Hz), 121.09 (d, *J* = 11.2 Hz), 111.38 (d, *J* = 25.6 Hz), 109.06. mp: 342.3–344.0 °C. HRMS (ESI^−^): *m*/*z*: calcd. for C_12_H_8_FN_5_O_3_ [M − H]^−^: 288.0538, found: 288.0528.

*6-(2-(2-Oxoindolin-3-ylidene)hydrazineyl)pyrimidine-2,4(1H,3H)-dione* (**6b**). Yellow solid, yield: 73%. ^1^H NMR (400 MHz, DMSO-*d*_6_) *δ*: 12.43 (s, 1H, Isatin-NH), 11.42 (s, 1H, Pyrimidine-NH), 11.30 (s, 1H, Pyrimidine-NH), 10.85 (s, 1H, NH), 7.82 (d, *J* = 7.5 Hz, 1H, PhH), 7.48 (t, *J* = 7.7 Hz, 1H, PhH), 7.21 (t, *J* = 7.5 Hz, 1H, PhH), 7.06 (d, *J* = 7.8 Hz, 1H, PhH), 5.60 (s, 1H, CH). ^13^C NMR (101 MHz, DMSO-*d*_6_) *δ*: 164.55, 162.79, 151.71, 151.04, 142.31, 134.71, 131.46, 122.79, 121.28, 120.54, 111.33, 78.52. mp: 335.5–337.1 °C. HRMS (ESI^−^): *m*/*z*: calcd. for C_12_H_9_N_5_O_3_ [M − H]^−^: 270.0627 found: 270.0605.

*6-(2-(5-Chloro-2-oxoindolin-3-ylidene)hydrazineyl)pyrimidine-2,4(1H,3H)-dione* (**6c**). Yellow solid, yield: 65%. ^1^H NMR (400 MHz, DMSO-*d*_6_) *δ*: 12.28 (s, 1H, Isatin-NH), 11.25 (s, 1H, Pyrimidine-NH), 11.23 (s, 1H, Pyrimidine-NH), 10.74 (s, 1H, NH), 7.87 (s, 1H, PhH), 7.35 (d, *J* = 8.3 Hz, 1H, PhH), 6.92 (d, *J* = 8.3 Hz, 1H, PhH), 5.55 (s, 1H, CH). ^13^C NMR (101 MHz, DMSO-*d*_6_) *δ*: 164.59, 162.49, 151.34, 151.05, 140.82, 133.16, 130.57, 126.97, 122.47, 121.20, 112.70, 79.59. mp: 344.8–346.4 °C. HRMS (ESI^−^): *m*/*z*: calcd. for C_12_H_8_ClN_5_O_3_ [M − H]^−^: 304.0243 found: 304.0229.

*6-(2-(5-Bromo-2-oxoindolin-3-ylidene)hydrazineyl)pyrimidine-2,4(1H,3H)-dione* (**6d**). Yellow solid, yield: 62%. ^1^H NMR (400 MHz, DMSO-*d*_6_) *δ*: 12.45 (s, 1H, Isatin-NH), 11.39 (s, 1H, Pyrimidine-NH), 11.32 (s, 1H, Pyrimidine-NH), 10.75 (s, 1H, NH), 7.32–7.22 (m, 2H, PhH), 6.96 (d, *J* = 7.2 Hz, 1H, PhH), 5.51 (s, 1H, CH).^13^C NMR (101 MHz, DMSO-d_6_) *δ*: 164.57, 162.32, 152.09, 150.95, 143.97, 134.09, 132.38, 127.11, 118.77, 115.65, 110.60, 78.52. mp: 330.2–333.1 °C. HRMS (ESI^−^): *m*/*z*: calcd. for C_12_H_8_BrN_5_O_3_ [M − H]^−^: 347.9732 found: 347.9729.

*6-(2-(5-Methyl-2-oxoindolin-3-ylidene)hydrazineyl)pyrimidine-2,4(1H,3H)-dione* (**6e**). Yellow solid, yield: 71%. ^1^H NMR (400 MHz, DMSO-*d*_6_) *δ* 12.28 (s, 1H, Isatin-NH), 11.27 (s, 1H, Pyrimidine-NH), 11.05 (s, 1H, Pyrimidine-NH), 10.70 (s, 1H, NH), 7.52 (s, 1H, PhH), 7.14 (d, *J* = 8.0, 1H, PhH), 6.81 (d, *J* = 7.9 Hz, 1H, PhH), 5.45 (s, 1H, CH), 2.30 (s, 3H CH_3_). ^13^C NMR (101 MHz, DMSO-*d*_6_) *δ*: 164.56, 162.88, 151.71, 151.05, 140.07, 134.82, 131.87, 131.84, 121.67, 120.54, 111.08, 78.47, 21.04. mp: 338.4–340.1 °C. HRMS (ESI^−^): *m*/*z*: calcd. for C_13_H_11_N_5_O_3_ [M − H]^−^: 284.0784, found: 284.0761.

*6-(2-(2-Oxo-5-(trifluoromethoxy)indolin-3-ylidene)hydrazineyl)pyrimidine-2,4(1H,3H)-dione* (**6f**). Yellow solid, yield: 58%. ^1^H NMR (400 MHz, DMSO-*d*_6_) *δ*: 12.85 (s, 1H, Isatin-NH), 11.55 (s, 1H, Pyrimidine-NH), 11.30 (s, 1H, Pyrimidine-NH), 9.71–9.46 (m, 1H, PhH), 7.96 (d, *J* = 9.2 Hz, 1H, PhH), 7.86 (dd, *J* = 9.2, 2.7 Hz, 1H, PhH). ^13^C NMR (101 MHz, Chloroform-*d*) *δ*: 158.82, 149.76, 149.39, 147.31, 139.39, 138.32, 132.12, 129.42, 123.91, 120.70, 119.15, 108.77. mp: 325.7–327.8 °C. HRMS (ESI^−^): *m*/*z*: calcd. for C_13_H_8_F_3_N_5_O_3_ [M − H]^−^: 354.0528 found: 354.0464.

*6-(2-(6-Fluoro-2-oxoindolin-3-ylidene)hydrazineyl)pyrimidine-2,4(1H,3H)-dione* (**6g**). Yellow solid, yield: 69%. ^1^H NMR (400 MHz, DMSO-*d*_6_) *δ*: 12.20 (s, 1H, Isatin-NH), 11.30 (s, 1H, Pyrimidine-NH), 11.26 (s, 1H, Pyrimidine-NH), 10.71 (s, 1H, NH), 7.73 (dd, *J* = 8.4, 5.6 Hz, 1H, PhH), 6.94–6.85 (m, 1H, PhH), 6.76 (dd, *J* = 9.1, 2.4 Hz, 1H, PhH), 5.47 (s, 1H, CH). ^13^C NMR (101 MHz, DMSO-*d*_6_) *δ*: 164.57, 162.98, 151.66, 151.03, 143.96 (d, *J* = 12.6 Hz), 133.69, 123.19 (d, *J* = 10.3 Hz), 117.04, 114.30, 109.46 (d, *J* = 23.3 Hz), 99.54 (d, *J* = 27.7 Hz), 78.63. mp: 341.2–344.2 °C. HRMS (ESI^−^): *m*/*z*: calcd. for C_12_H_8_FN_5_O_3_ [M − H]^−^: 288.0538, found: 288.0539.

*6-(2-(6-Chloro-2-oxoindolin-3-ylidene)hydrazineyl)pyrimidine-2,4(1H,3H)-dione* (**6h**). Yellow solid, yield: 64%. ^1^H NMR (400 MHz, DMSO-*d*_6_) *δ*: 10.97 (s, 1H, Isatin-NH), 10.81 (s, 1H, Pyrimidine-NH), 10.65 (s, 1H, Pyrimidine-NH), 10.18 (s, 1H, NH), 8.02 (d, *J* = 8.2 Hz, 1H, PhH), 7.20 (dd, *J* = 8.2, 2.0 Hz, 1H, PhH), 6.94 (d, *J* = 1.9 Hz, 1H, PhH), 5.32 (s, 1H, CH). ^13^C NMR (101 MHz, DMSO-*d*_6_) *δ*: 164.55, 162.69, 151.55, 151.00, 143.40, 135.36, 133.56, 122.68, 122.63, 119.57, 111.34, 78.95. mp: 345.6–347.3 °C. HRMS (ESI^−^): *m*/*z*: calcd. for C_12_H_8_ClN_5_O_3_ [M − H]^−^: 304.0061, found: 304.0173.

*6-(2-(6-Bromo-2-oxoindolin-3-ylidene)hydrazineyl)pyrimidine-2,4(1H,3H)-dione* (**6i**). Yellow solid, yield: 70%. ^1^H NMR (400 MHz, DMSO-*d*_6_) *δ*: 12.26 (s, 1H, Isatin-NH), 11.28 (s, 2H, Pyrimidine-NH), 10.74 (s, 1H, NH), 7.66 (d, *J* = 8.1 Hz, 1H, PhH), 7.27 (d, *J* = 8.1 Hz, 1H, PhH), 7.08 (s, 1H, PhH), 5.49 (s, 1H, CH). ^13^C NMR (101 MHz, DMSO-*d*_6_) *δ*: 164.54, 162.57, 151.54, 151.01, 143.49, 133.63, 125.48, 123.88, 122.93, 119.94, 114.08, 79.00. mp: 335.8–338.1 °C. HRMS (ESI^−^): *m*/*z*: calcd. for C_12_H_8_BrN_5_O_3_ [M − H]^−^: 349.9781, found: 349.9720.

*6-(2-(2-Oxo-6-(trifluoromethoxy)indolin-3-ylidene)hydrazineyl)pyrimidine-2,4(1H,3H)-dione* (**6j**). Yellow solid, yield: 61%. ^1^H NMR (400 MHz, DMSO-*d*_6_) *δ*: 12.83 (s, 1H, Isatin-NH), 11.58 (s, 1H, Pyrimidine-NH), 11.31 (s, 1H, Pyrimidine-NH), 9.76 (d, *J* = 9.3 Hz, 1H, PhH), 7.69 (s, 1H, PhH), 7.62 (d, *J* = 9.3 Hz, 1H, PhH). ^13^C NMR (101 MHz, Chloroform-*d*) *δ*: 158.70, 154.89, 149.58, 148.26, 141.26, 139.47, 132.49, 131.35, 121.95, 117.65, 111.27, 107.84. mp: 356.7–358.1°C. HRMS (ESI^−^): *m*/*z*: calcd. for C_13_H_8_F_3_N_5_O_3_ [M − H]^−^: 354.0450, found: 354.0447.

*6-(2-(7-Fluoro-2-oxoindolin-3-ylidene)hydrazineyl)pyrimidine-2,4(1H,3H)-dione* (**6k**). Yellow solid, yield: 63%. ^1^H NMR (400 MHz, DMSO-*d*_6_) *δ*: 12.31 (s, 1H, Isatin-NH), 11.67 (s, 1H, Pyrimidine-NH), 11.30 (s, 1H, Pyrimidine-NH), 10.75 (s, 1H, NH), 7.56 (d, *J* = 7.4 Hz, 1H, PhH), 7.26 (dd, *J* = 10.5, 8.3 Hz, 1H, PhH), 7.08 (td, *J* = 8.0, 4.6 Hz, 1H, PhH), 5.50 (s, 1H, CH). ^13^C NMR (101 MHz, DMSO-*d*_6_) *δ* 164.54, 162.56, 151.54, 151.00, 133.83, 129.07 (d, *J* = 13.7 Hz), 123.58, 117.99 (d, *J* = 17.4 Hz), 117.26 (d, *J* = 27.5 Hz), 114.25, 79.04. mp: 343.6–346.1 °C. HRMS (ESI^−^): *m*/*z*: calcd. for C_12_H_8_FN_5_O_3_ [M − H]^−^: 288.0533, found: 288.0516.

*6-(2-(7-Chloro-2-oxoindolin-3-ylidene)hydrazineyl)pyrimidine-2,4(1H,3H)-dione* (**6l**). Yellow solid, yield: 59%. ^1^H NMR (400 MHz, DMSO-*d*_6_) *δ*: 12.30 (s, 1H, Isatin-NH), 11.59 (s, 1H, Pyrimidine-NH), 11.29 (s, 1H, Pyrimidine-NH), 10.76 (s, 1H, NH), 7.69 (d, *J* = 7.5 Hz, 1H, PhH), 7.41 (dd, *J* = 8.2, 1.0 Hz, 1H, PhH), 7.09 (t, *J* = 7.8 Hz, 1H, PhH), 5.51 (s, 1H, CH). ^13^C NMR (101 MHz, DMSO-*d*_6_) *δ*: 164.53, 162.79, 151.52, 150.99, 139.55, 133.92, 130.81, 123.93, 122.63, 119.84, 115.43, 79.13. mp: 326.8–328.2 °C. HRMS (ESI^−^): *m*/*z*: calcd. for C_12_H_8_ClN_5_O_3_ [M − H]^−^: 304.0237, found: 304.0255.

*6-(2-(7-Bromo-2-oxoindolin-3-ylidene)hydrazineyl)pyrimidine-2,4(1H,3H)-dione* (**6m**). Yellow solid, yield: 66%. ^1^H NMR (400 MHz, DMSO-*d*_6_) *δ*: 12.31 (s, 1H, Isatin-NH), 11.46 (s, 1H, Pyrimidine-NH), 11.30 (s, 1H, Pyrimidine-NH), 10.76 (s, 1H, NH), 7.72 (d, *J* = 7.5 Hz, 1H, PhH), 7.53 (d, *J* = 8.1 Hz, 1H, PhH), 7.03 (t, *J* = 7.8 Hz, 1H, PhH), 5.51 (s, 1H, CH). ^13^C NMR (101 MHz, DMSO-*d*_6_) *δ*: 164.60, 162.79, 151.56, 150.99, 141.24, 134.10, 133.72, 124.26, 122.63, 119.85, 103.61, 79.07. mp: 332.6–334.8 °C. HRMS (ESI^−^): *m*/*z*: calcd. for C_12_H_8_BrN_5_O_3_ [M − H]^−^: 347.9732, found: 347.9722.

*6-(2-(7-Methyl-2-oxoindolin-3-ylidene)hydrazineyl)pyrimidine-2,4(1H,3H)-dione* (**6n**). Yellow solid, yield: 72%. ^1^H NMR (400 MHz, DMSO-*d*_6_) *δ*: 12.33 (s, 1H, Isatin-NH), 11.25 (s, 1H, Pyrimidine-NH), 11.22 (s, 1H, Pyrimidine-NH), 10.71 (s, 1H, NH), 7.52 (d, *J* = 7.4 Hz, 1H, PhH), 7.17 (d, *J* = 7.4 Hz, 1H, PhH), 6.99 (t, *J* = 7.6 Hz, 1H, PhH), 5.46 (s, 1H, CH), 2.23 (s, 3H, CH_3_). ^13^C NMR (101 MHz, DMSO-*d*_6_) *δ*: 164.55, 163.25, 151.71, 151.03, 140.95, 135.11, 132.69, 122.78, 120.72, 120.22, 118.71, 78.45, 16.38. mp: 330.1–333.5 °C. HRMS (ESI^−^): *m*/*z*: calcd. for C_13_H_11_N_5_O_3_ [M − H]^−^: 284.0784, found: 284.0761.

*6-(2-(2-Oxo-7-(trifluoromethyl)indolin-3-ylidene)hydrazineyl)pyrimidine-2,4(1H,3H)-dione* (**6o**). Yellow solid, yield: 60%. ^1^H NMR (400 MHz, DMSO-*d*_6_) *δ*: 12.30 (s, 1H, Isatin-NH), 11.58 (s, 1H, Pyrimidine-NH), 11.29 (s, 1H, Pyrimidine-NH), 10.77 (s, 1H, NH), 8.02 (d, *J* = 7.4 Hz, 1H, PhH), 7.62 (d, *J* = 7.9 Hz, 1H, PhH), 7.26 (d, *J* = 7.7 Hz, 1H, PhH), 5.56 (s, 1H, CH). ^13^C NMR (101 MHz, DMSO-*d*_6_) *δ*: 164.55, 163.03, 151.47, 150.99, 150.26, 140.71, 138.91, 132.58, 124.96, 122.74, 122.52, 79.42. mp: 317.5–319.3 °C. HRMS (ESI^−^): *m*/*z*: calcd. for C_13_H_8_F_3_N_5_O_3_ [M − H]^−^: 339.0528, found: 339.0513.

*6-(2-(5-Methoxy-2-oxoindolin-3-ylidene)hydrazineyl)pyrimidine-2,4(1H,3H)-dione* (**6p**). Yellow solid, yield: 73%. ^1^H NMR (400 MHz, DMSO-*d*_6_) *δ*: 12.32 (s, 1H, Isatin-NH), 11.25 (s, 1H, Pyrimidine-NH), 10.97 (s, 1H, Pyrimidine-NH), 10.73 (s, 1H, NH), 7.33 (d, 1H, PhH), 6.91 (d, *J* = 5.9 Hz, 1H, PhH), 6.83 (d, *J* = 8.5 Hz, 1H, PhH), 5.52 (s, 1H, CH), 3.83 (s, 3H, OCH_3_). ^13^C NMR (101 MHz, DMSO-*d*_6_) *δ*: 164.59, 162.94, 155.75, 151.06, 135.96, 134.88, 121.32, 117.70, 112.09, 106.44, 78.75, 56.11. mp: 343.9–345.6 °C. HRMS (ESI^−^): *m*/*z*: calcd. for C_13_H_11_N_5_O_4_ [M − H]^−^: 300.0733, found: 300.0717.

*6-(2-(6-Methoxy-2-oxoindolin-3-ylidene)hydrazineyl)pyrimidine-2,4(1H,3H)-dione* (**6q**). Yellow solid, yield: 61%. ^1^H NMR (400 MHz, DMSO-*d*_6_) *δ*: 12.14 (s, 1H, Isatin-NH), 11.22 (s, 1H, Pyrimidine-NH), 11.12 (s, 1H, Pyrimidine-NH), 10.66 (s, 1H, NH), 7.59 (d, *J* = 8.5 Hz, 1H, PhH), 6.64 (dd, *J* = 8.4, 2.3 Hz, 1H, PhH), 6.46 (d, *J* = 2.2 Hz, 1H, PhH), 5.41 (s, 1H, CH), 3.81 (s, 3H, OCH_3_). ^13^C NMR (101 MHz, DMSO-*d*_6_) *δ*: 164.56, 163.44, 162.58, 151.82, 151.06, 144.14, 134.65, 122.80, 113.10, 108.55, 97.73, 77.88, 56.02. mp: 346.3–346.8 °C. HRMS (ESI^−^): *m*/*z*: calcd. for C_13_H_11_N_5_O_4_ [M − H]^−^: 300.0733, found: 300.0714.

*6-(2-(7-Methoxy-2-oxoindolin-3-ylidene)hydrazineyl)pyrimidine-2,4(1H,3H)-dione* (**6r**). Yellow solid, yield: 69%. ^1^H NMR (400 MHz, DMSO-*d*_6_) *δ*: 12.34 (s, 1H, Isatin-NH), 11.37–11.19 (m, 2H, Pyrimidine-NH), 10.71 (s, 1H, NH), 7.29 (t, *J* = 5.8 Hz, 1H, PhH), 7.05 (dt, *J* = 9.6, 4.4 Hz, 2H, PhH), 5.45 (s, 1H, CH), 3.85 (d, *J* = 4.9 Hz, 3H, OCH_3_).^13^C NMR (101 MHz, DMSO-*d*_6_) *δ*: 164.54, 162.74, 151.70, 151.03, 144.64, 135.03, 130.94, 123.54, 121.21, 114.41, 113.60, 78.53, 56.26. mp: 341.1–344.8 °C. HRMS (ESI^−^): *m*/*z*: calcd. for C_13_H_11_N_5_O_4_ [M − H]^−^: 300.0733, found: 300.0710.

*6-(2-(5,6-Difluoro-2-oxoindolin-3-ylidene)hydrazineyl)pyrimidine-2,4(1H,3H)-dione* (**6s**). Yellow solid, yield: 69%. ^1^H NMR (400 MHz, DMSO-*d*_6_) *δ*: 12.22 (s, 1H, Isatin-NH), 11.25 (s, 1H, Pyrimidine-NH), 11.23 (s, 1H, Pyrimidine-NH), 10.74 (s, 1H, NH), 7.89 (dd, *J* = 10.0, 8.1 Hz, 1H, PhH), 6.98 (dd, *J* = 10.4, 6.5 Hz, 1H, PhH), 5.54 (s, 1H, CH). ^13^C NMR (101 MHz, DMSO-*d*_6_) *δ*: 164.57, 162.71, 151.40, 151.02, 138.91 (d, *J* = 10.8 Hz), 132.95, 116.89 (d, *J* = 11.3 Hz), 114.27, 110.70 (d, *J* = 21.0 Hz), 101.27 (d, *J* = 23.3 Hz), 79.39. mp: 341.5–344.1 °C. HRMS (ESI^−^): *m*/*z*: calcd. for C_12_H_7_F_2_N_5_O_3_ [M − H]^−^: 306.0517, found: 306.0450.

*6-(2-(5,6-Dichloro-2-oxoindolin-3-ylidene)hydrazineyl)pyrimidine-2,4(1H,3H)-dione* (**6t**). Yellow solid, yield: 63%. ^1^H NMR (400 MHz, DMSO-*d*_6_) *δ*: 12.25 (s, 1H, Isatin-NH), 11.35 (s, 1H, Pyrimidine-NH), 11.25 (s, 1H, Pyrimidine-NH), 10.77 (s, 1H, NH), 8.08 (s, 1H, PhH), 7.11 (s, 1H, PhH), 5.59 (s, 1H, CH). ^13^C NMR (101 MHz, DMSO-*d*_6_) *δ*: 158.36, 150.82, 150.21, 147.70, 140.68, 134.97, 128.35, 128.18, 120.02, 109.37, 49.06. mp: 336.5–338.1 °C. HRMS (ESI^−^): *m*/*z*: calcd. for C_12_H_7_Cl_2_N_5_O_3_ [M − H]^−^: 337.9848, found: 337.9832.

*6-(2-(5,6-Dibromo-2-oxoindolin-3-ylidene)hydrazineyl)pyrimidine-2,4(1H,3H)-dione* (**6u**). Yellow solid, yield: 66%. ^1^H NMR (400 MHz, DMSO-*d*_6_) *δ*: 12.27 (s, 1H, Isatin-NH), 11.35 (s, 1H, Pyrimidine-NH), 11.26 (s, 1H, Pyrimidine-NH), 10.77 (s, 1H, NH), 8.18 (s, 1H, PhH), 7.25 (s, 1H, PhH), 5.59 (s, 1H, CH). ^13^C NMR (101 MHz, DMSO-*d*_6_) *δ*: 164.63, 162.25, 151.23, 151.03, 142.15, 132.19, 125.74, 125.39, 122.21, 116.76, 115.90, 79.96. mp: 341.2–344.5 °C. HRMS (ESI^−^): *m*/*z*: calcd. for C_12_H_7_Br_2_N_5_O_3_ [M − H]^−^: 427.8817, found: 427.8807.

*6-(2-(5,7-Dimethyl-2-oxoindolin-3-ylidene)hydrazineyl)pyrimidine-2,4(1H,3H)-dione* (**6v**). Yellow solid, yield: 71%. ^1^H NMR (400 MHz, DMSO-*d*_6_) *δ*: 12.31 (s, 1H, Isatin-NH), 11.18 (m, 2H, Pyrimidine-NH), 10.70 (s, 1H, NH), 7.35 (s, 1H, PhH), 6.98 (s, 1H, PhH), 5.45 (s, 1H, CH), 2.26 (s, 3H, CH_3_), 2.18 (s, 3H, CH_3_). ^13^C NMR (101 MHz, DMSO-*d*_6_) *δ*: 164.56, 163.32, 151.72, 151.03, 138.73, 135.22, 133.30, 131.78, 120.41, 120.25, 119.10, 78.39, 20.95, 16.27. mp: 343.8–345.3 °C. HRMS (ESI^−^): *m*/*z*: calcd. for C_14_H_13_Br_2_N_5_O_3_ [M − H]^−^: 298.0940, found: 298.0921.

*5-(2-(5,6-Dichloro-2-oxoindolin-3-ylidene)hydrazineyl)pyrimidine-2,4(1H,3H)-dione* (**11a**). Yellow solid, yield: 83%.^1^H NMR (400 MHz, DMSO-*d_6_*) *δ* 12.29 (s, 1H, Isatin-NH), 11.68 (s, 1H, Pyrimidine-NH), 11.24 (s, 1H, Pyrimidine-NH), 11.13–10.98 (s, 1H, NH), 7.74 (s, 1H, PhH), 7.61 (s, 1H, CH), 7.08 (s, 1H, PhH). ^13^C NMR (101 MHz, DMSO-*d_6_*) *δ*: 163.57, 159.76, 150.19, 139.46, 130.20, 126.47, 124.44, 122.65, 121.97, 120.37, 118.36, 112.55. mp: 250.7–252.4 °C. HRMS (ESI^−^): *m*/*z*: calcd. for C_12_H_7_Cl_2_N_5_O_3_ [M − H]^−^: 337.9848, found: 337.9829.

*5-(2-(5,6-Dibromo-2-oxoindolin-3-ylidene)hydrazineyl)pyrimidine-2,4(1H,3H)-dione* (**11b**). Yellow solid, yield: 70%.^1^H NMR (400 MHz, DMSO-d_6_) *δ* 12.29 (s, 1H, Isatin-NH), 11.68 (s, 1H, Pyrimidine-NH), 11.21 (s, 1H, Pyrimidine-NH), 11.09 (s, 1H, NH), 7.82 (s, 1H, PhH), 7.66–7.54 (m, 1H, CH), 7.20 (s, 1H, PhH). ^13^C NMR (101 MHz, DMSO-*d*_6_) *δ* 163.39, 159.75, 150.20, 140.00, 126.36, 123.21, 122.75, 122.66, 122.58, 118.35, 116.20, 115.50. mp: 255.3–258.1 °C. HRMS (ESI^−^): *m*/*z*: calcd. for C_12_H_7_Br_2_N_5_O_3_ [M − H]^−^: 427.8817, found: 427.8805.

### 4.2. Biology

#### 4.2.1. Expression and Purification of Recombinant HIV-1 RT

HIV-1 group M subtype B heterodimeric reverse transcriptase (RT) was expressed and purified according to previously established protocols. Briefly, Escherichia coli strain M15 harboring the p6HRT-Prot expression vector was cultured to an optical density of 0.7 at 600 nm, followed by induction with 1.7 mM isopropyl β-d-1-thiogalactoside (IPTG) for 4 h. The expressed RT protein was purified using a BioLogic LP system (Bio-Rad Laboratories, Hercules, CA, USA) through a combination of immobilized metal affinity chromatography (IMAC) and ion exchange chromatography (IEX).

For cell lysis, the harvested bacterial pellet was resuspended in a lysis buffer consisting of 50 mM sodium phosphate (pH = 7.8) supplemented with 0.5 mg/mL lysozyme. After incubation on ice for 20 min, NaCl was added to a final concentration of 0.3 M, and the cell lysate was subjected to ultrasonication and centrifugation at 30,000× *g* for 1 h. The resulting supernatant was applied to a Ni^2+^-NTA-Sepharose column pre-equilibrated with a binding buffer (50 mM sodium phosphate, 0.3 M NaCl, 10% glycerol, and 10 mM imidazole, pH = 7.8). The column was washed with a washing buffer (50 mM sodium phosphate, 0.3 M NaCl, 10% glycerol, and 80 mM imidazole, pH 6.0) to remove nonspecifically bound proteins.

RT was eluted using a gradient of the wash buffer supplemented with increasing concentrations of imidazole. The purity of the collected fractions was assessed by SDS-PAGE, demonstrating protein purity exceeding 90%.

To further purify the enzyme, the pooled RT fractions were diluted 1:1 with a 50 mM sodium phosphate buffer (pH = 7.0, containing 10% glycerol) and loaded onto a HiTrap Heparin HP GE column (Marlborough, MA, USA) pre-equilibrated with the same buffer. The RT protein was eluted with a high-salt elution buffer (50 mM sodium phosphate, 10% glycerol, 1 M NaCl, pH = 7.0). Subsequently, the purified protein was dialyzed against 50 mM Tris-HCl buffer (pH = 7.0) containing 25 mM NaCl, 1 mM EDTA, and 50% glycerol. The enzymatic activity and protein concentration of the purified RT were quantified, and the enzyme-containing fractions were aliquoted and stored at −80 °C for long-term use.

#### 4.2.2. Determination of HIV-1 RNase H Activity

The tested compounds were initially dissolved in dimethyl sulfoxide (DMSO) at a concentration of 50 mM and subsequently serially diluted (ratio 1:3). The compounds were tested as previously described [20].

Briefly, a reaction mixture containing 50 mM Tris-HCl buffer pH 7.8, 6 mM MgCl_2_, 1 mM dithiothreitol (DTT), 80 mM KCl, 0.25 μM hybrid RNA/DNA. 25 μM of RNA/DNA hybrid 5′-GAUCUGAGCCUGGAGCU-Fluorescin-3′ (HPLC, dry, QC: Mass Check), 5′-Dabcyl-AGCTCCCAGGCTCAGATC-3′ (HPLC, dry, QC: Mass Check), and increasing concentrations of inhibitors was diluted in DMSO. The activity of the RNase H function associated with HIV-1 RT was quantified by measuring the linear portion of the dose–response curve. The reaction mixture was incubated at 37 °C for 10 min with a Victor Nivo 5 multilabel plate reader (model 1420-051, PerkinElmer, Waltham, MA, USA), and fluorescence signals were recorded at excitation/emission wavelengths of 490/528 nm. Based on the obtained fluorescence data, the inhibitory concentration 50 (IC_50_) of the target compounds was calculated by interpolation on a dose–response curve. Results are reported as average and standard deviation of at least two independent replicates in triplicate.

#### 4.2.3. In Vitro Anti-HIV Assay

The anti-HIV-1 (strain IIIB) activity evaluation in MT-4 cells was conducted using an optimized MTT assay. The cell lines were obtained from the laboratory (Prof. Erik De Clercq, Rega Institute for Medical Research, KU Leuven, Herestraat 49, Leuven, Belgium). Compounds were serially diluted (5-fold increments, starting from 10× concentration) and dispensed into 96-well plates (25 µL/well) via an automated Biomek 3000 workstation (Beckman Instruments, Fullerton, CA, USA). Parallel experimental groups included virus-infected (50 µL HIV inoculum, 100–300 TCID50) and mock-infected controls (50 µL culture medium). MT-4 cells were centrifuged (220× *g*, 5 min), resuspended to 6 × 10⁵ cells/mL, and seeded (50 µL/well). After a 5-day incubation, cell viability was quantified by measuring mitochondrial dehydrogenase-mediated MTT-to-formazan conversion using dual-wavelength spectrophotometry (540 nm primary, 690 nm reference). The 50% cytotoxic concentration (CC_50_) was calculated from triplicate median absorbance values, representing the compound concentration reducing mock-infected cell viability by 50%.

#### 4.2.4. Determination of Inhibitors Kinetic Parameters

Kinetic analysis of the DNA-polymerase independent RNase H activity was performed as already reported [20]. Briefly, a reaction mixture containing 50 mM Tris-HCl buffer pH 7.8, 6 mM MgCl_2_, 1 mM DTT, 80 mM KCl, and increasing concentrations of hybrid RNA/DNA 5′-GAUCUGAGCCUGGGAGCU-Fluorescin-3′/5′-Dabcyl-AGCTCCCAGGCTCAGATC-3′ was incubated at 37 °C for 10 min with a Victor Nivo 5 multilabel plate reader (model 1420-051, PerkinElmer, Waltham, MA, USA), and fluorescence signals were recorded at excitation/emission wavelengths of 490/528 nm. Results were represented according to Michaelis Menten and Lineaweaver–Burke plots.

## Figures and Tables

**Table 1:** Activity of compounds **6a**–**6v** and **11a**–**11b** on HIV-1 RT-associated enzymatic functions RNase H.

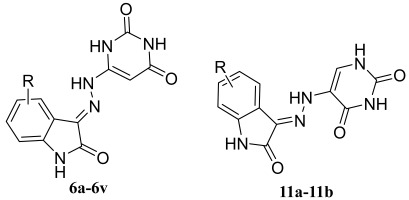			
Compd	IC_50_ [μM] *^a^*	Compd	IC_50_ [μM] *^a^*
**6a**	81.23 ± 11.36	**6m**	43.78 ± 7.92
**6b**	>100	**6n**	25.49 ± 1.37
**6c**	58.76 ± 2.40	**6o**	24.06 ± 7.02
**6d**	>100	**6p**	>100
**6e**	>100	**6q**	>100
**6f**	8.94 ± 1.43	**6r**	>100
**6g**	56.84 ± 15.90	**6s**	64.90 ± 0.77
**6h**	>100	**6t**	7.85 ± 0.55
**6i**	23.59 ± 1.11	**6u**	11.83 ± 0.81
**6j**	15.28 ± 0.01	**6v**	23.58 ± 3.89
**6k**	>100	**11a**	1.90 ± 0.01
**6l**	46.65 ± 3.25	**11b**	2.20 ± 0.20

*^a^* Concentration required to inhibit 50% of in vitro RNase H activity. Reported values are the average of three independent determinations.

**Table 2:** Anti-HIV-1 activity and cytotoxicity of **6t**, **11a** and **11b** on MT-4 cells.

Compd	HIV-1(III_B_) EC_50_ (μM) *^a^*	CC_50_ (μM) *^b^*	SI *^c^*
**6t**	109.71	294.37	3
**11a**	38.97	402.2	10
**11b**	44.86	348.93	8
NVP	0.19	>15.02	>80
EFV	0.0030	>4.59	>1538

*^a^* EC_50_: The effective concentration required to protect MT-4 cells against HIV-induced cytopathogenicity by 50%. *^b^* CC_50_: The cytotoxic concentration of the compound that reduced the normal uninfected MT-4 cell viability by 50%. *^c^* SI: selectivity index, ratio CC_50_/EC_50_.

## Data Availability

Data is contained within the article and Supplementary Materials.

## Supplementary Materials

The following supporting information can be downloaded at https://www.mdpi.com/article/10.3390/molecules30091868/s1. ^1^H & ^13^C NMR spectra and HRMS data for all synthesized compounds.
