# Supplementary material for: Design, Synthesis and Biological Evaluation of 3-Hydrazonoindolin-2-one Derivatives as Novel HIV-1 RNase H Inhibitors
Source: Molecules. 2025 Apr 22;30(9):1868. doi: 10.3390/molecules30091868 (PMC12073785; doi:10.3390/molecules30091868)
Supplement: Supplementary file 1 [file molecules-30-01868-s001.zip › molecules-3452319-supplementary.pdf]

Supporting information

Design, Synthesis and Biological Evaluation of 3-Hydrazonoindolin-2-one Derivatives as Novel HIV-1 RNase H Inhibitors

Yiying Zhang [1,2], Rao Wang [3], Yueyue Bu [3], Angela Corona [4], Laura Dettori [4,5], Enzo Tramontano [4], Christophe Pannecouque [6], Erik De Clercq [6], Shuai Wang [1,2], Ge Meng [1,2,7,*] and Fen-Er Chen [1,2,*]

1 Engineering Center of Catalysis and Synthesis for Chiral Molecules, Department of Chemistry, Fudan University, Shanghai 200433, China
2 Shanghai Engineering Center of Industrial Asymmetric Catalysis for Chiral Drugs, Shanghai 200433, China
3 Henan Key Laboratory of Nanomedicine for Targeting Diagnosis and Treatment, School of Pharmaceutical Sciences, Zhengzhou University, Zhengzhou 450001, China
4 Department of Life and Environmetal Sciences, University of Cagliari, 09042 Monserrato, Italy;
5 National PhD Programme in One Health Approaches to Infectious Diseases and Life Science Research, Department of Public Health, Experimental and Forensic Medicine, University of Pavia, 27100 Pavia, Italy
6 Rega Institute for Meical Research, KU Leuven, Herestraat 49, B-3000 Leuven, Belgium
7 College of Tea (Pu'er), West Yunnan University of Applied Sciences, Pu'er 665000, China

*Corresponding Author:

Fen-Er Chen (rfchen@fudan.edu.cn)

Ge Meng (mgfudan@fudan.edu.cn)

Table of contents

1. Molecular docking 2
2. ^{1}H NMR, ^{13}C NMR, and HRMS of target compounds 3

1. Molecular docking

The modelling study was conducted using Schrödinger Maestro. For RNase H, we chose the crystal structure of the HIV-1 RT in complex with an RNase H inhibitor and nevirapine available in the Protein Data Bank (PDB code: 3QIP). The three-dimensional structure of the ligand was generated using the Maestro and prepared for docking with Ligprep. The protonation states were predicted using the Epik program. The structures of the proteins were prepared using the "Protein Preparation Wizard" panel of the Schrödinger molecular modelling package. Initially, bond orders were defined and all the hydrogen atoms were added. Subsequently, a prediction of the ionization and tautomeric states of the amino acids side chains was conducted using Epik. Next, the hydrogen-bonding network was optimized, followed by a restrained minimization on hydrogen atoms. Last, all the water molecules were deleted. Then the docking studies were performed using Glide suite in Schrödinger Maestro. Visualized molecular docking results were generated by PyMol (http://pymol.sourceforge.net/).

2. ^{1}H NMR, ^{13}C NMR, and HRMS of target compounds

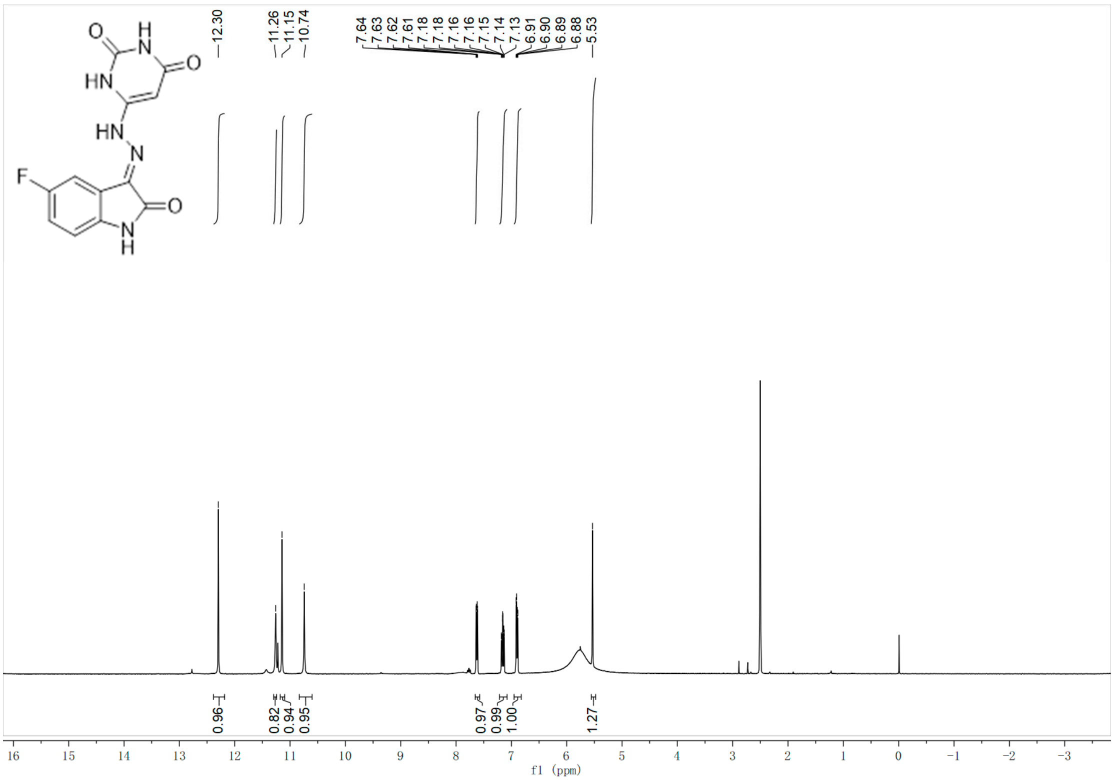

^{1}H NMR spectrum of **6a**.

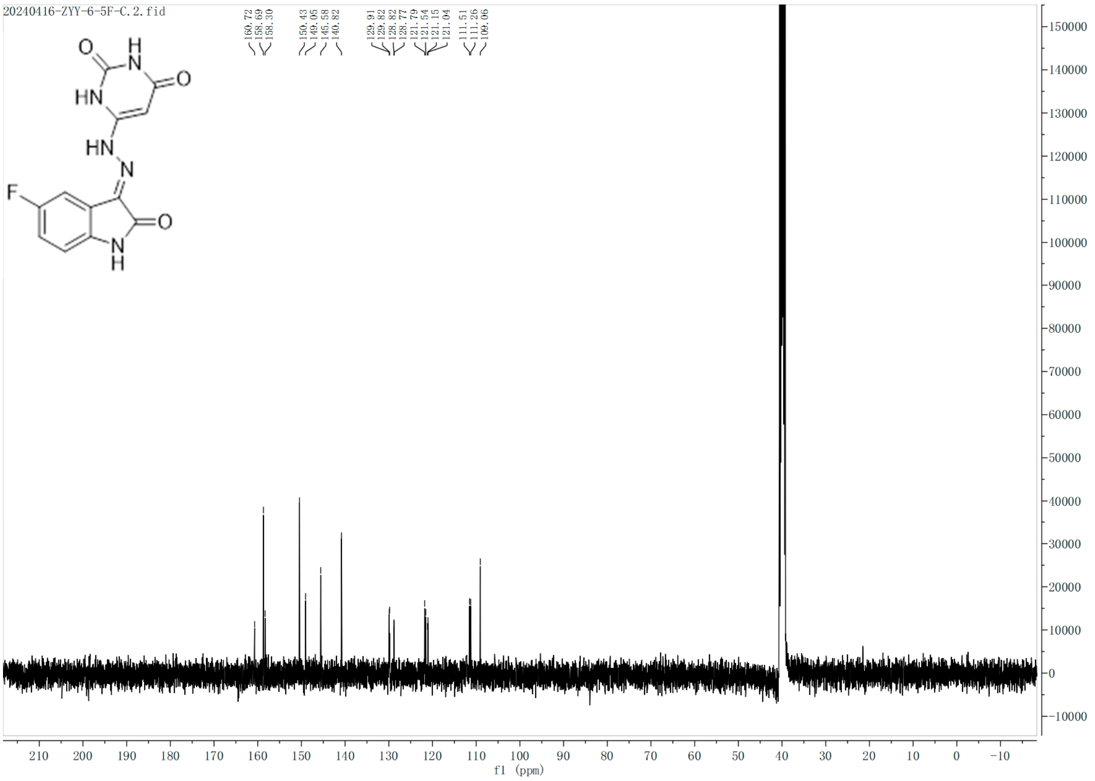

^{13}C NMR spectrum of **6a**.

Display Report

Analysis Info Acquisition Date 1/15/2024 10:38:28 AM

Sample Name 20240112-ZYY-6-5f
Comment

Acquisition Parameter

Source Type	ESI	Ion Polarity	Negative	Set Nebulizer	2.0 Bar
Focus	Active	Set Capillary	2800 V	Set Dry Heater	200 °C
Scan Begin	50 m/z	Set End Plate Offset	-500 V	Set Dry Gas	8.0 l/min
Scan End	1500 m/z	Set Charging Voltage	2000 V	Set Divert Valve	Waste
		Set Corona	0 nA	Set APCI Heater	0 °C

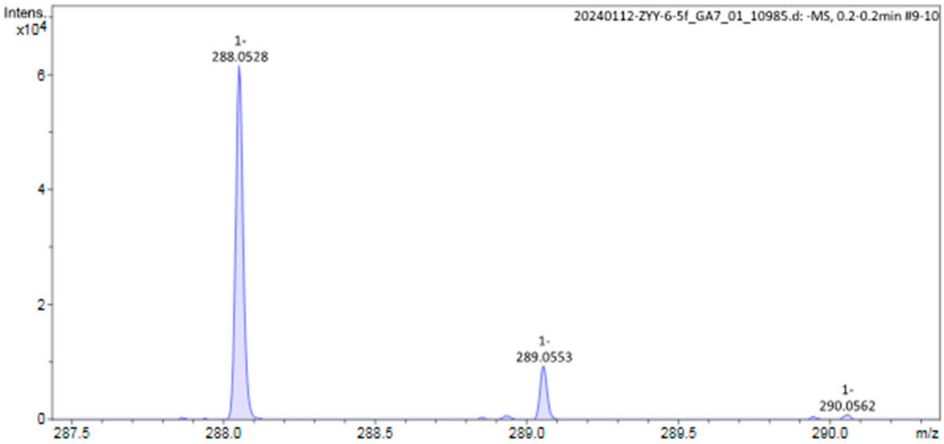

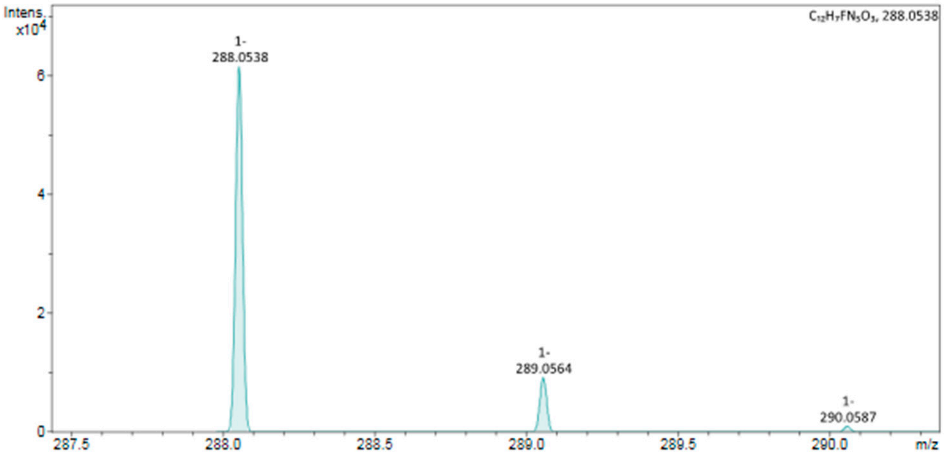

20240112-ZYY-6-5f_GA7_01_10985.d

printed: 1/15/2024 11:00:19 AM Page 1 of 1

HRMS spectrum of 6a.

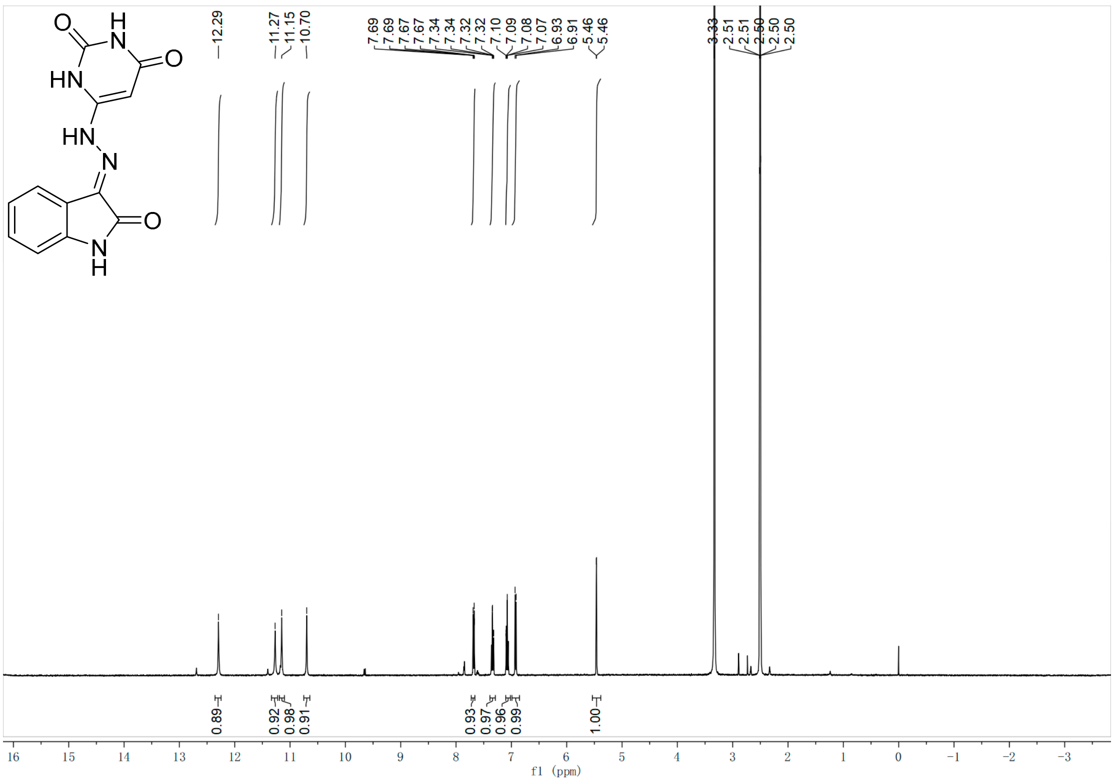

^{1}H NMR spectrum of **6b**.

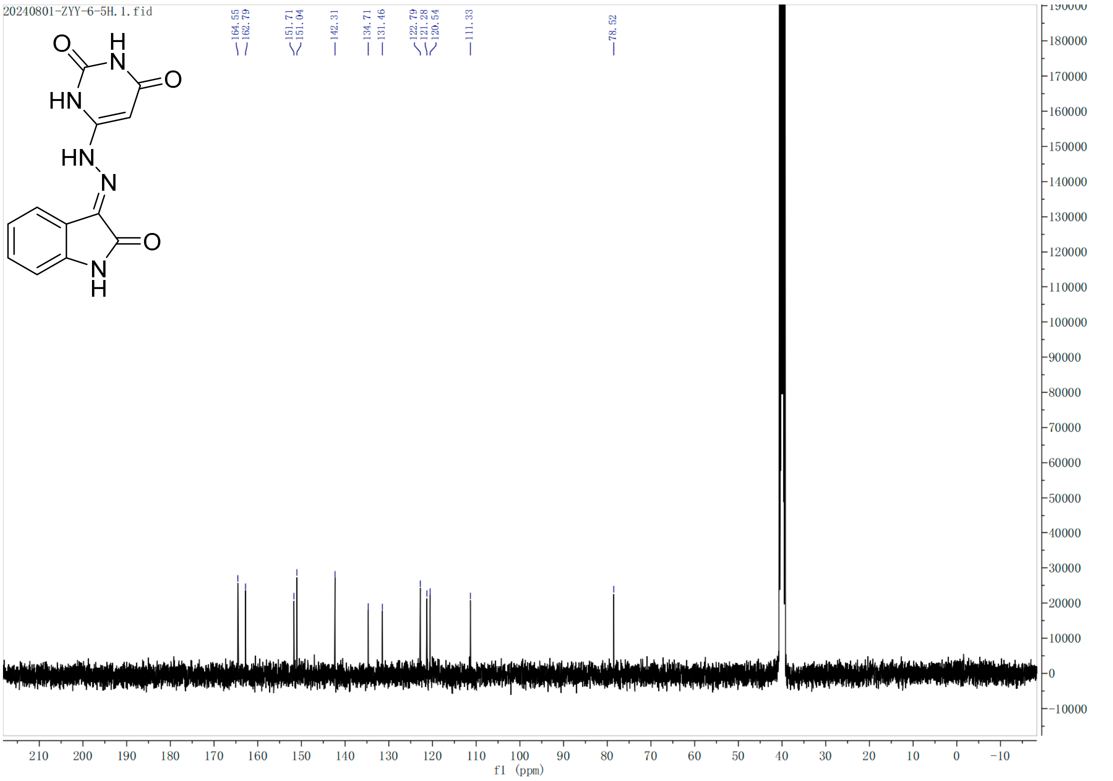

^{13}C NMR spectrum of **6b**.

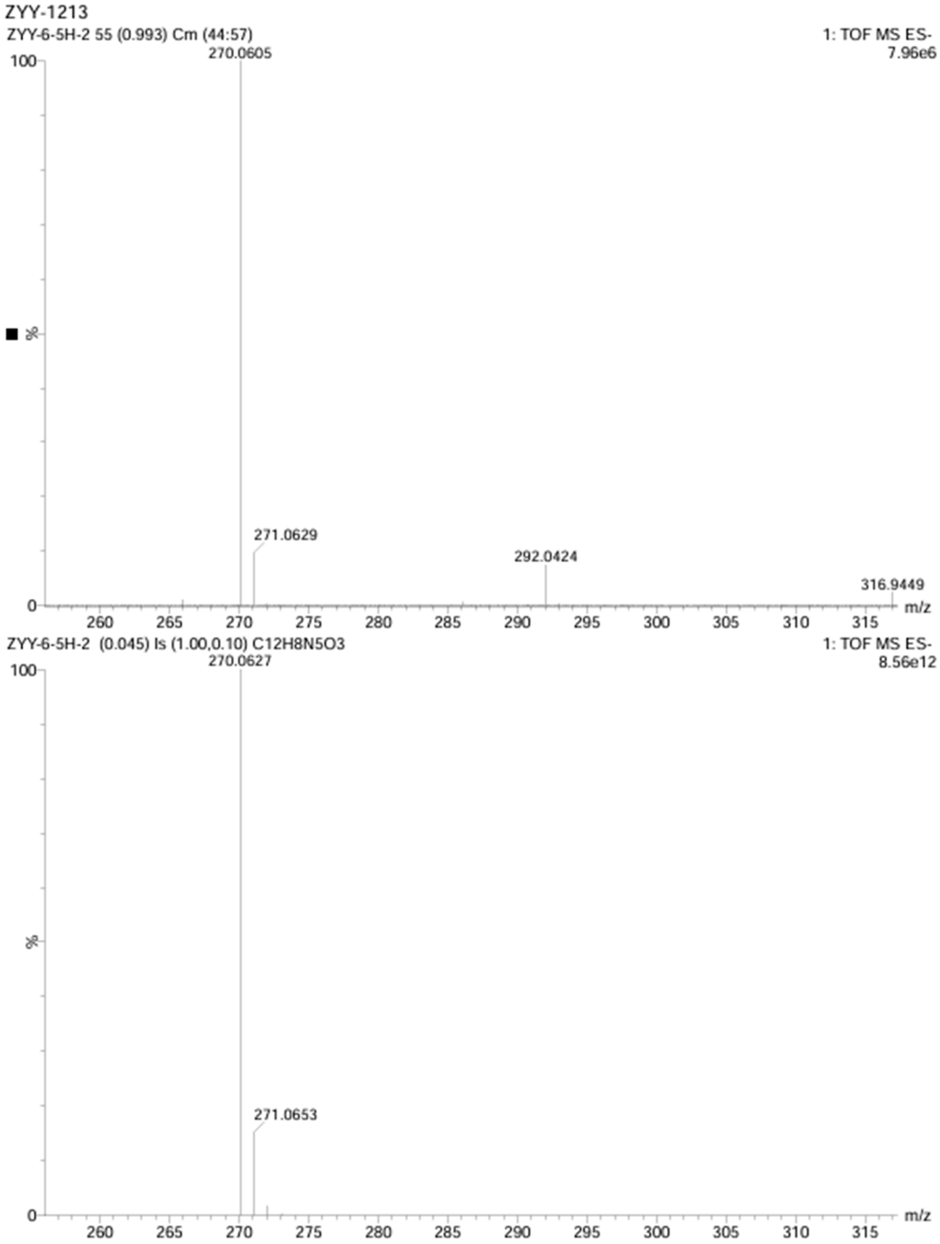

HRMS spectrum of **6b**.

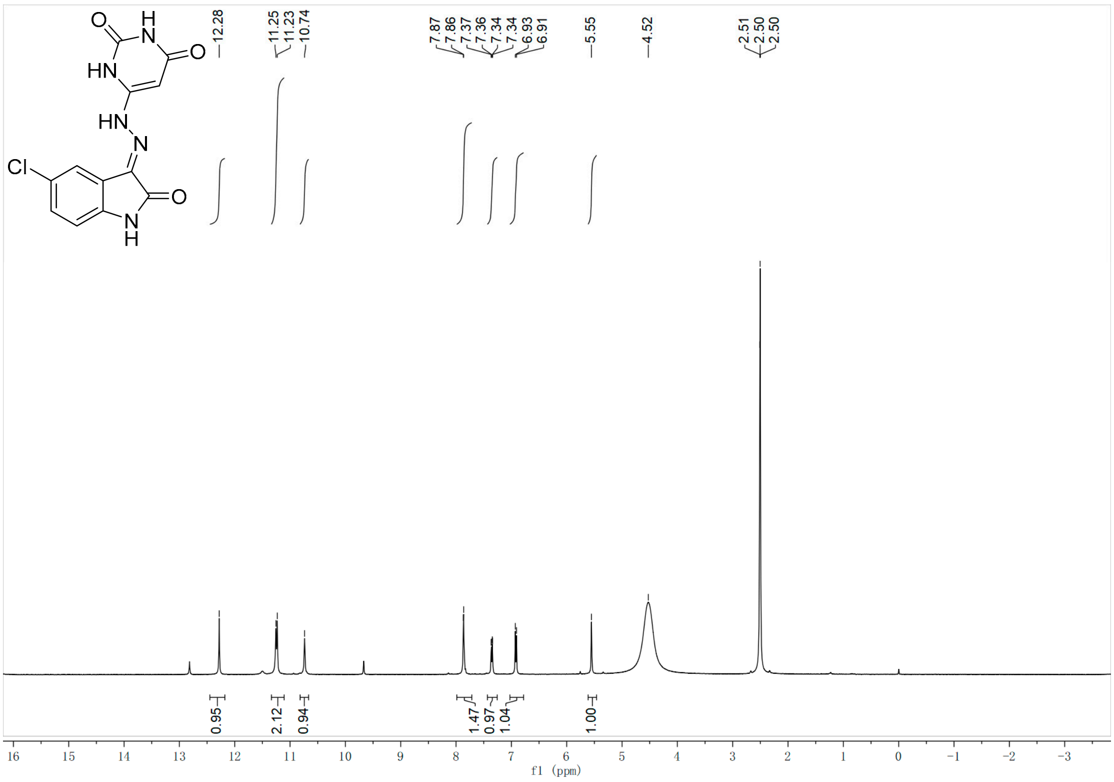

^{1}H NMR spectrum of **6c**.

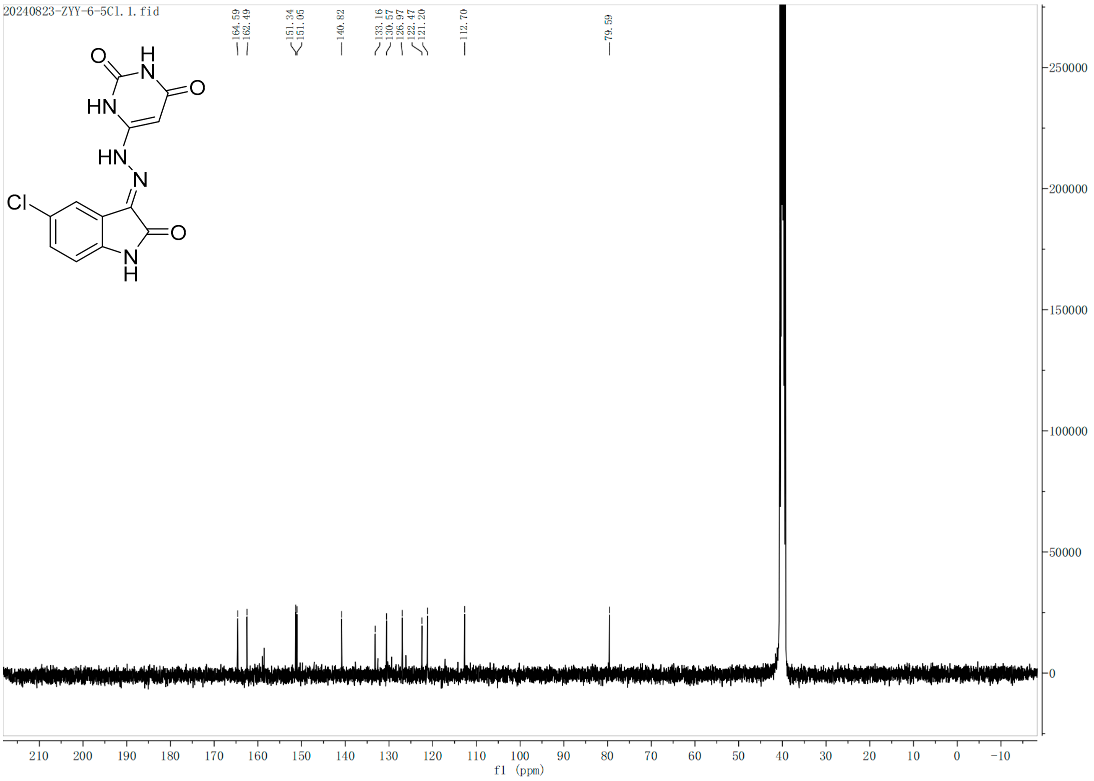

^{13}C NMR spectrum of **6c**.

Display Report

Analysis Info Acquisition Date 1/15/2024 11:01:40 AM

Sample Name 20240112-ZYY-6NH2-5Cl
Comment

Acquisition Parameter

Source Type	ESI	Ion Polarity	Negative	Set Nebulizer	2.0 Bar
Focus	Active	Set Capillary	2800 V	Set Dry Heater	200 °C
Scan Begin	50 m/z	Set End Plate Offset	-500 V	Set Dry Gas	8.0 l/min
Scan End	1500 m/z	Set Charging Voltage	2000 V	Set Divert Valve	Waste
		Set Corona	0 nA	Set APCI Heater	0 °C

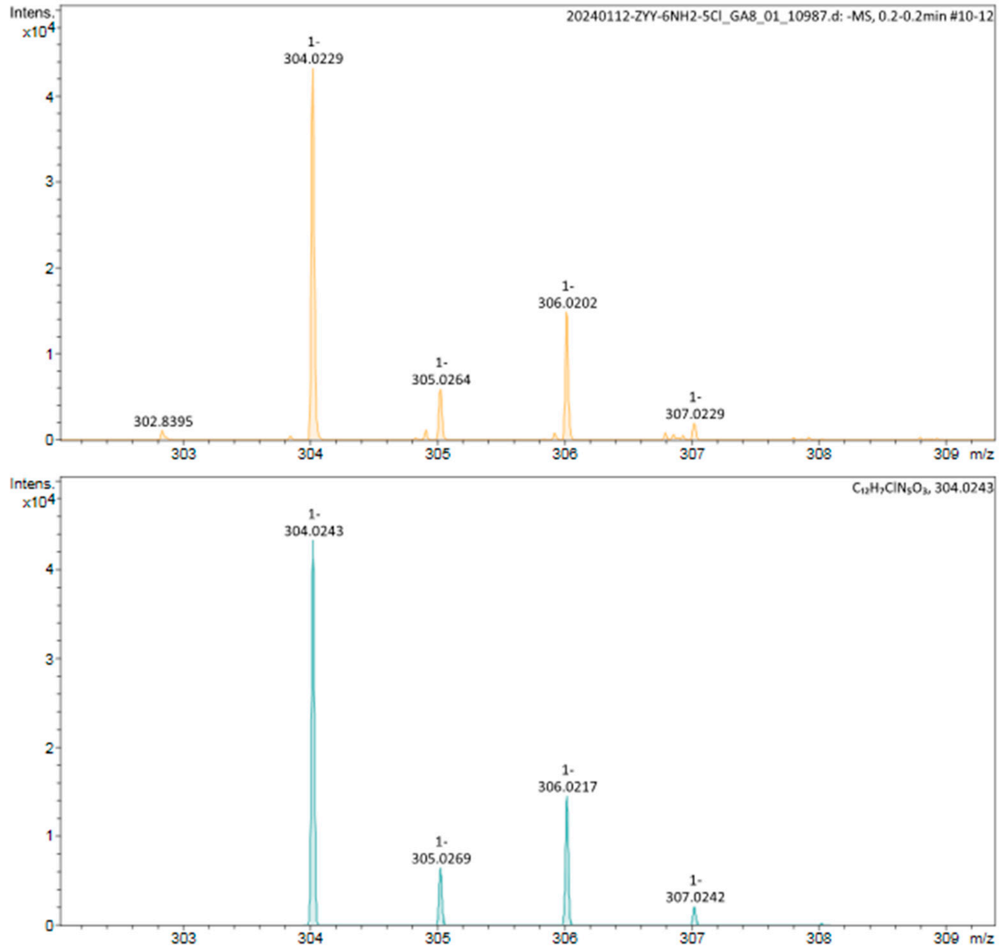

20240112-ZYY-6NH2-5Cl_GA8_01_10987.d
printed: 1/15/2024 11:25:53 AM Page 1 of 1

HRMS spectrum of **6c**.

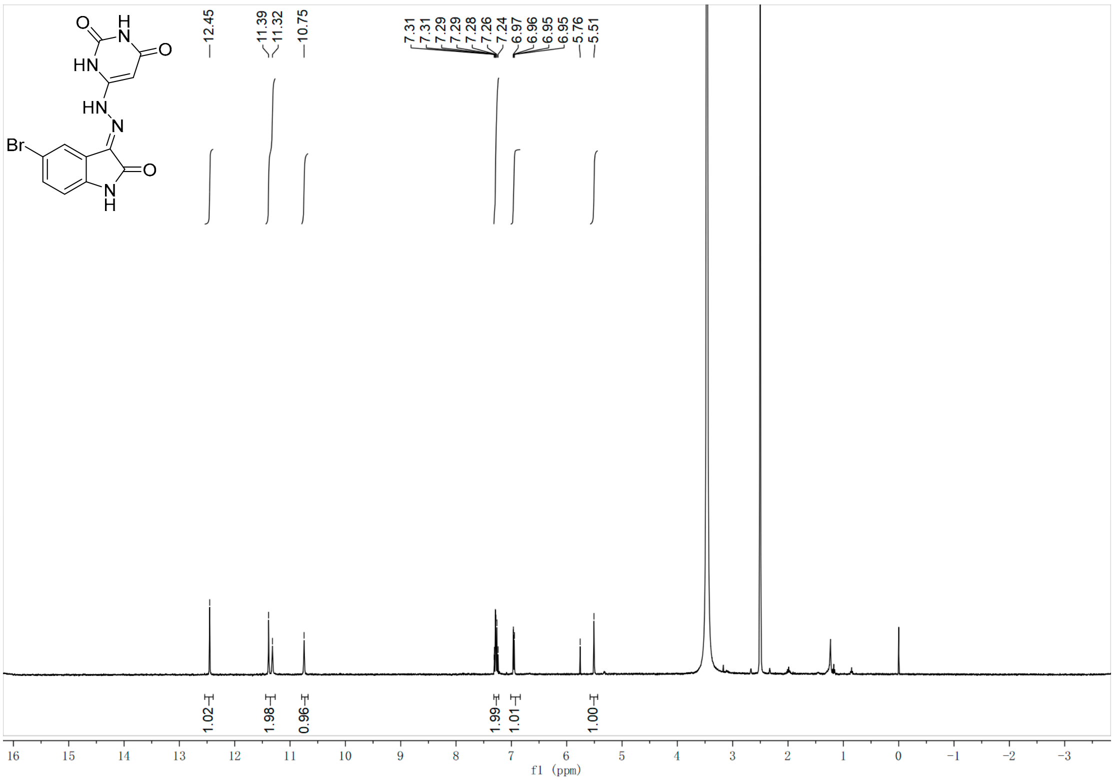

^1H NMR spectrum of **6d**.

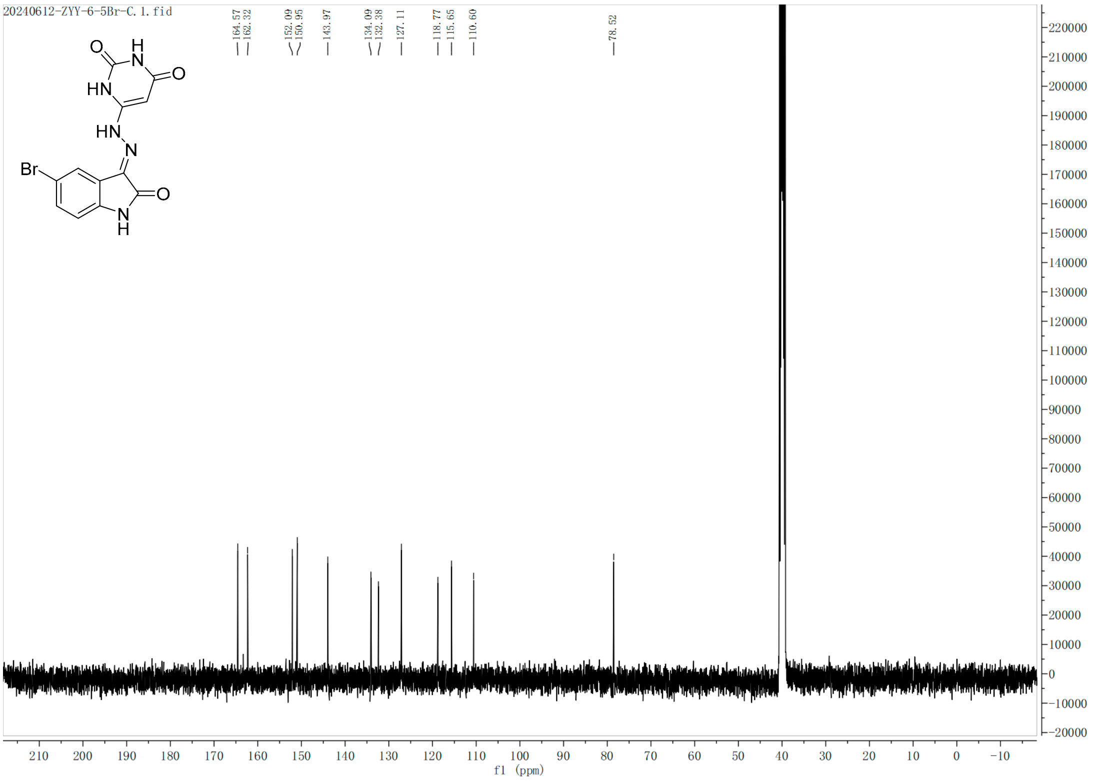

^{13}C NMR spectrum of **6d**.

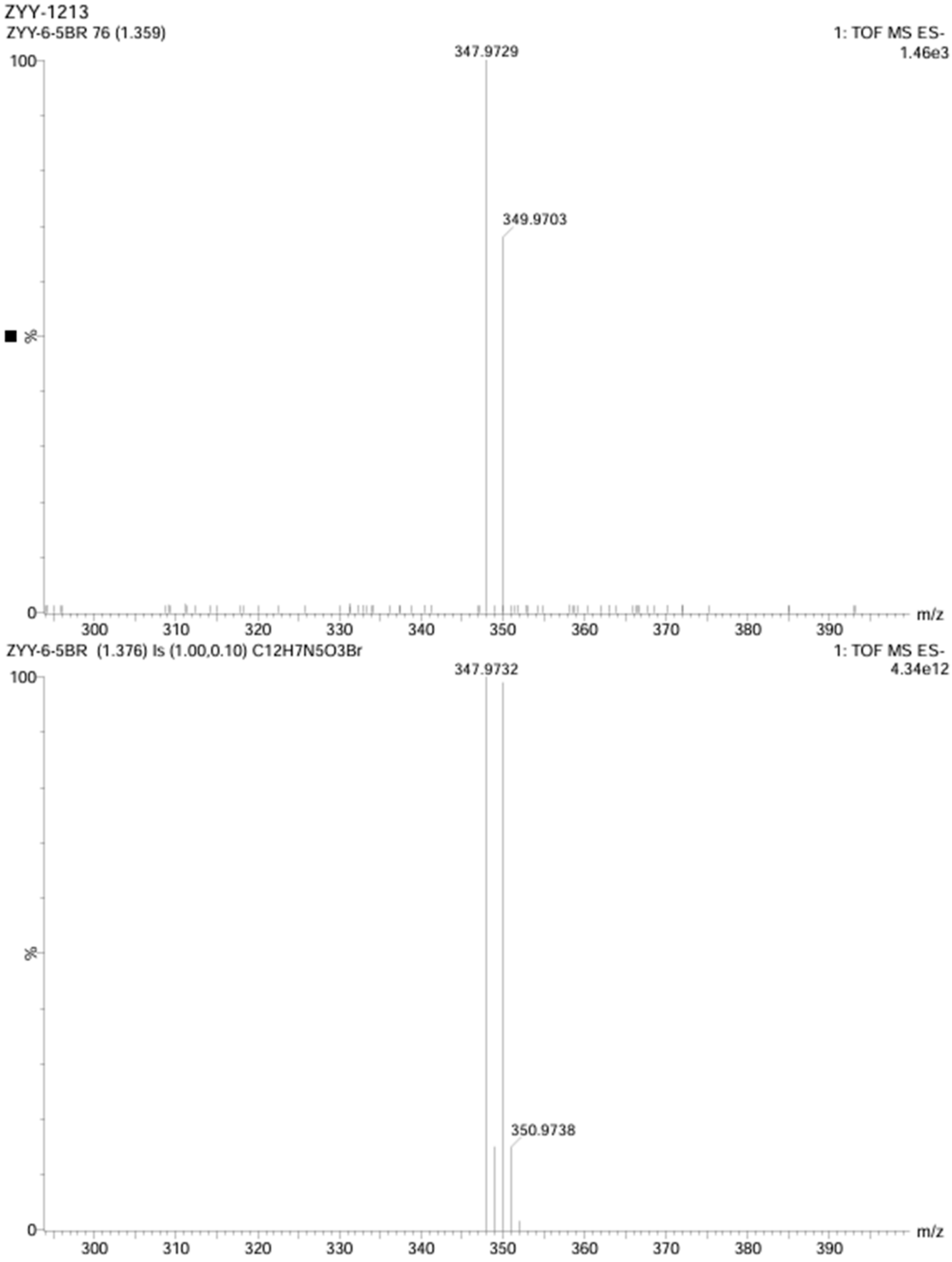

HRMS spectrum of **6d**.

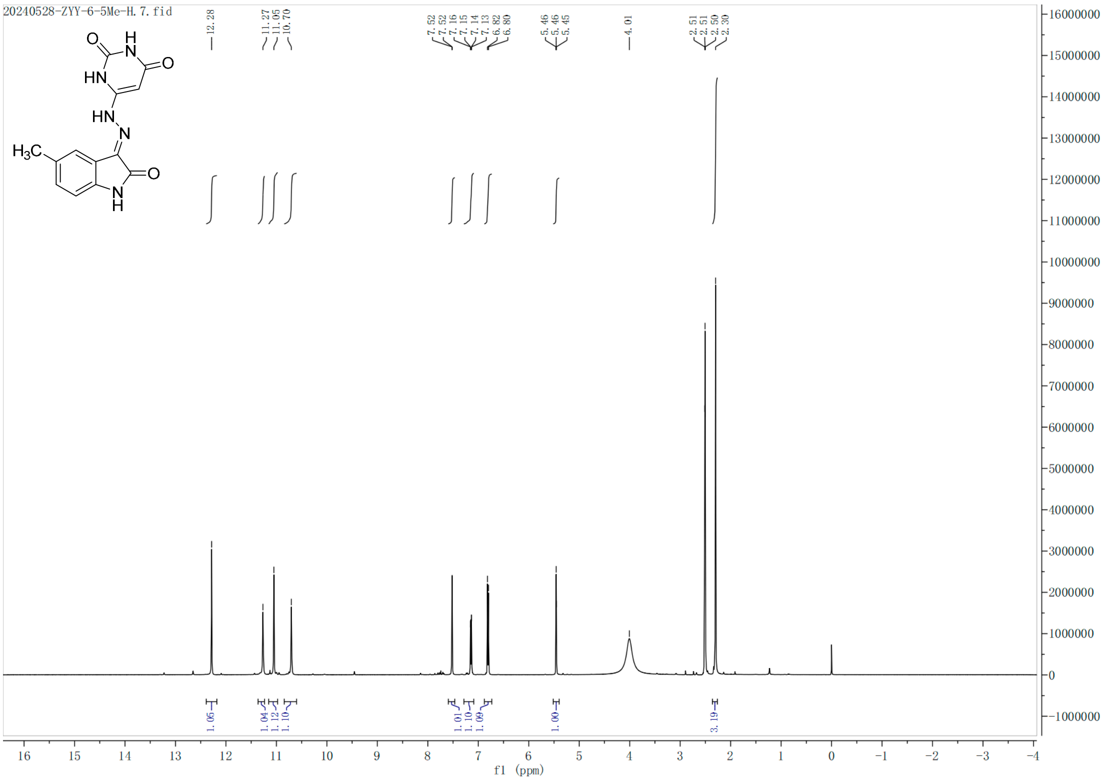

^{1}H NMR spectrum of **6e**.

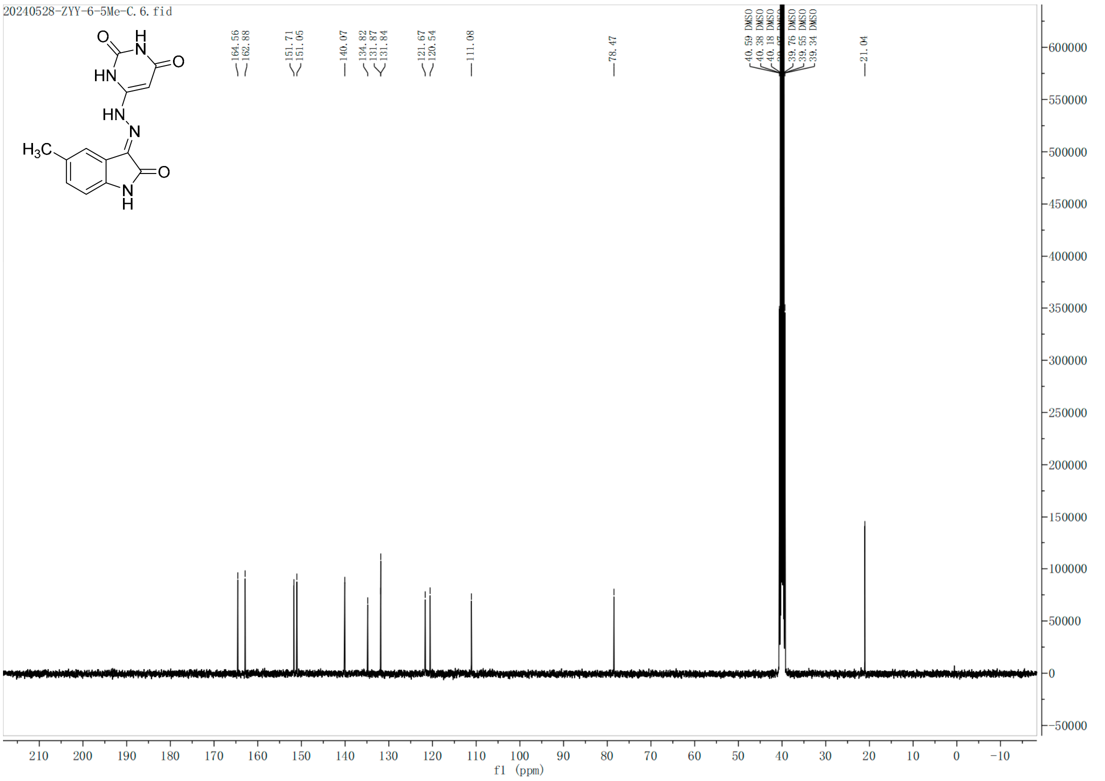

^{13}C NMR spectrum of **6e**.

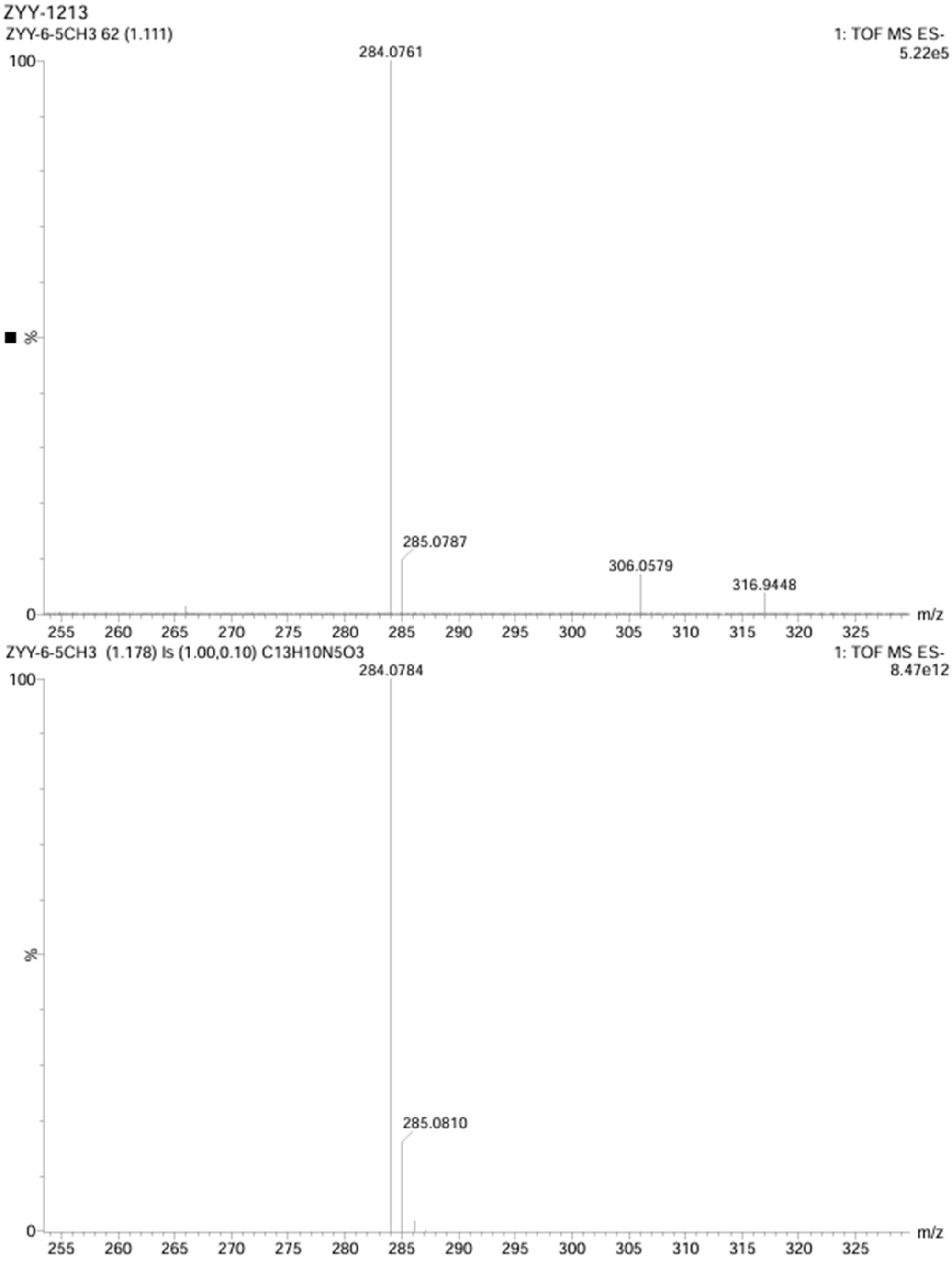

HRMS spectrum of **6e**.

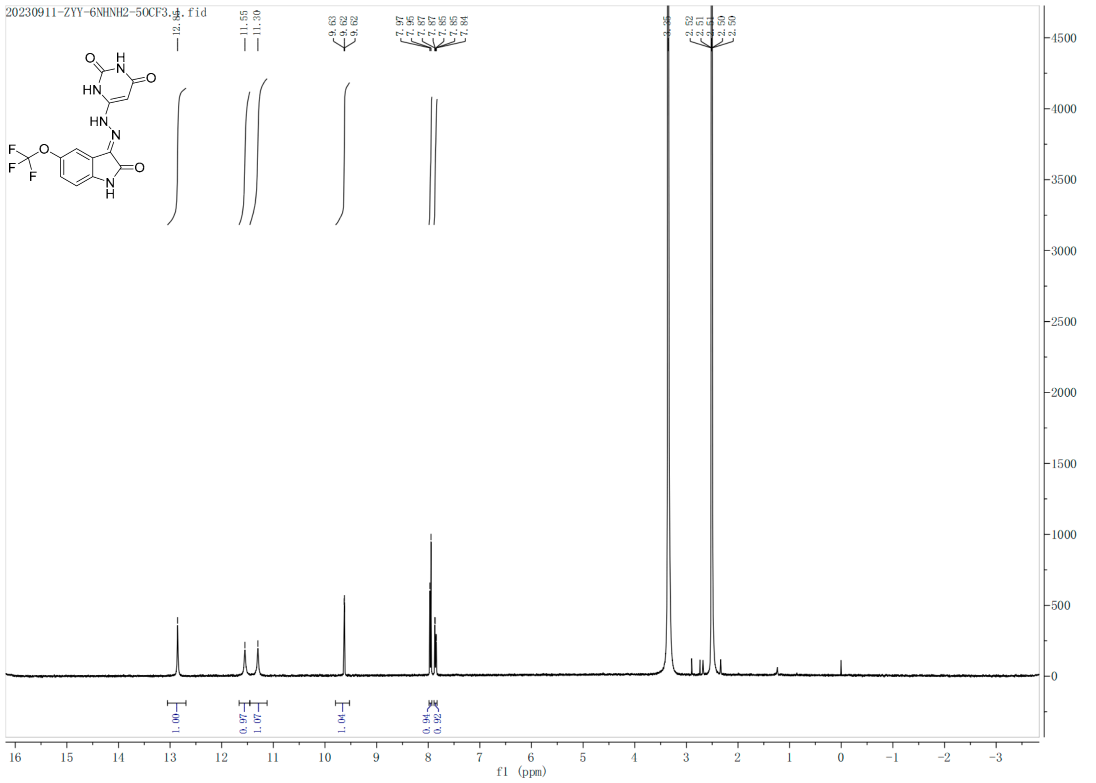

^{1}H NMR spectrum of **6f**.

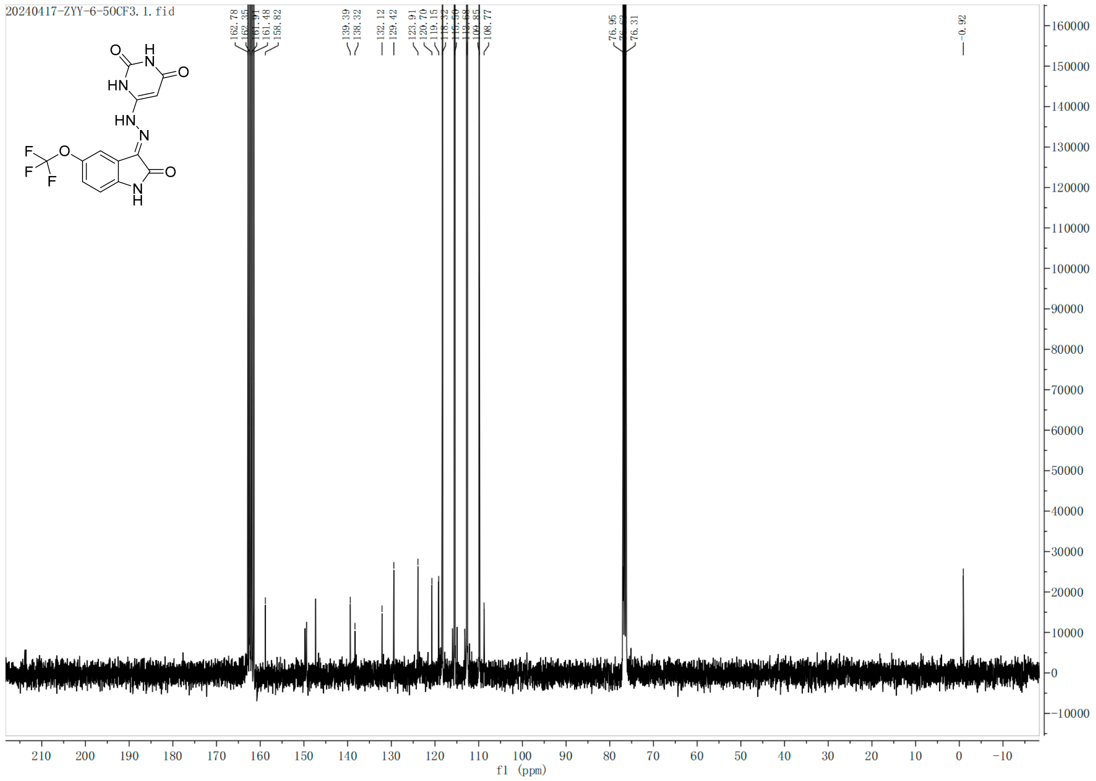

^{13}C NMR spectrum of **6f**.

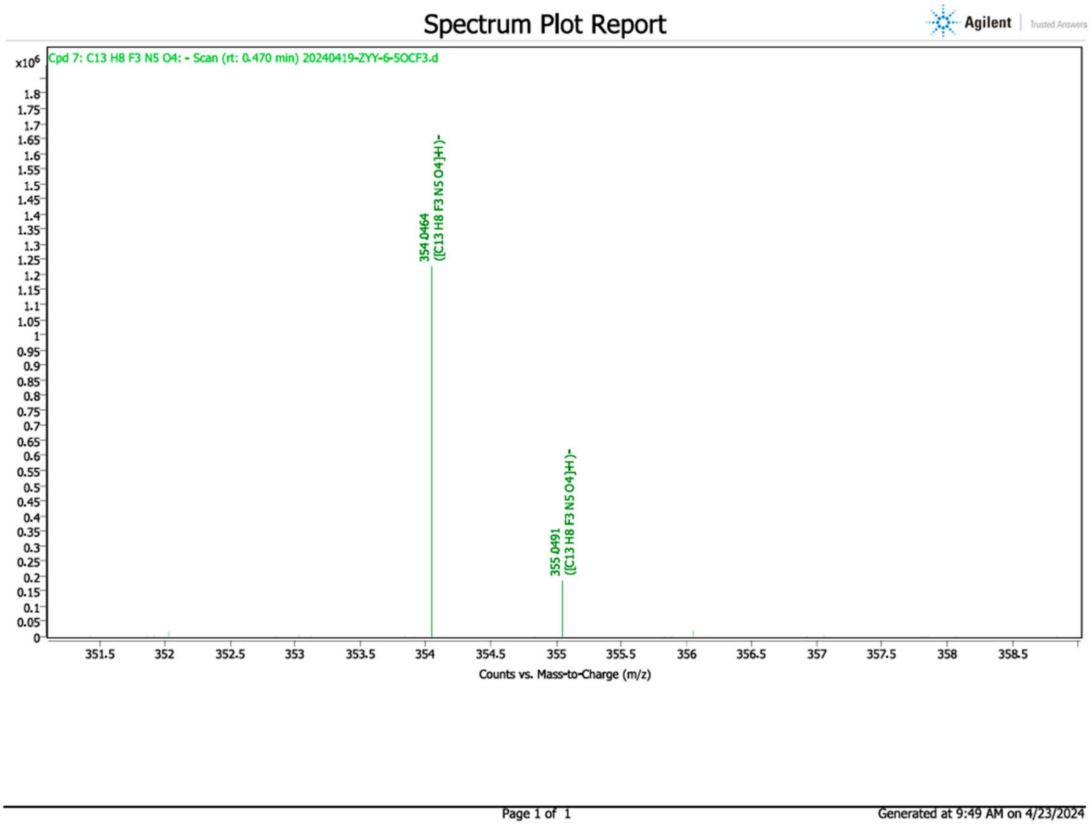

HRMS spectrum of **6f**.

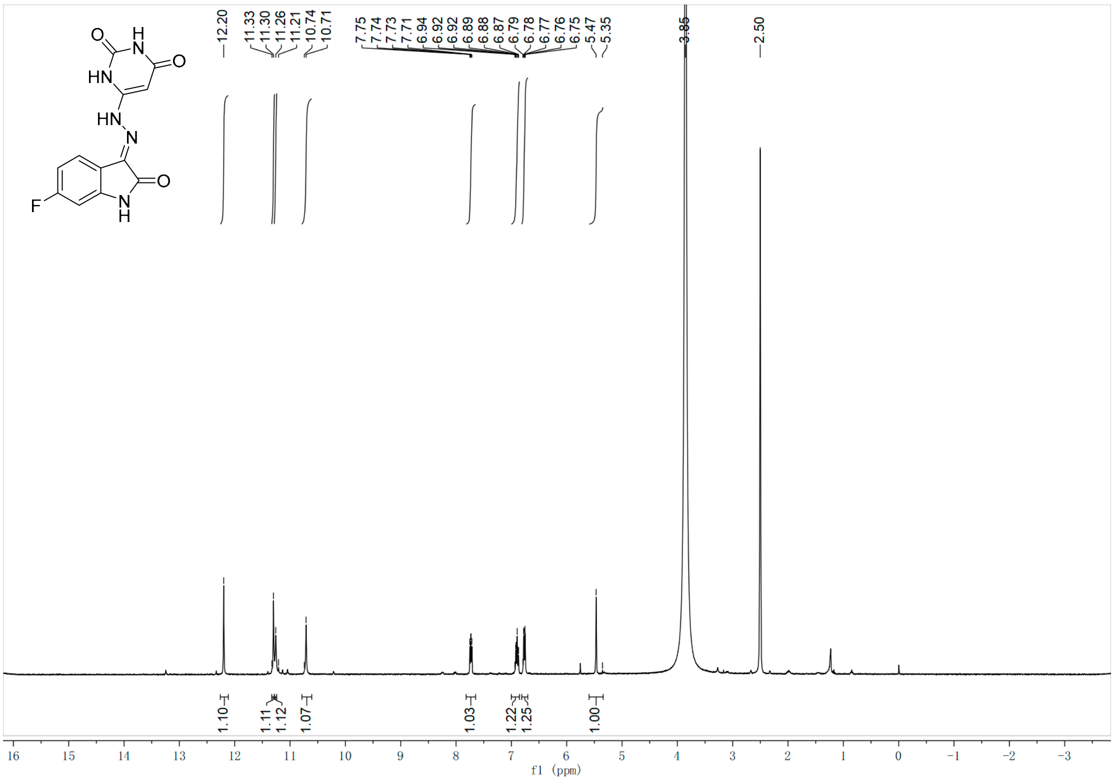

^{1}H NMR spectrum of **6g**.

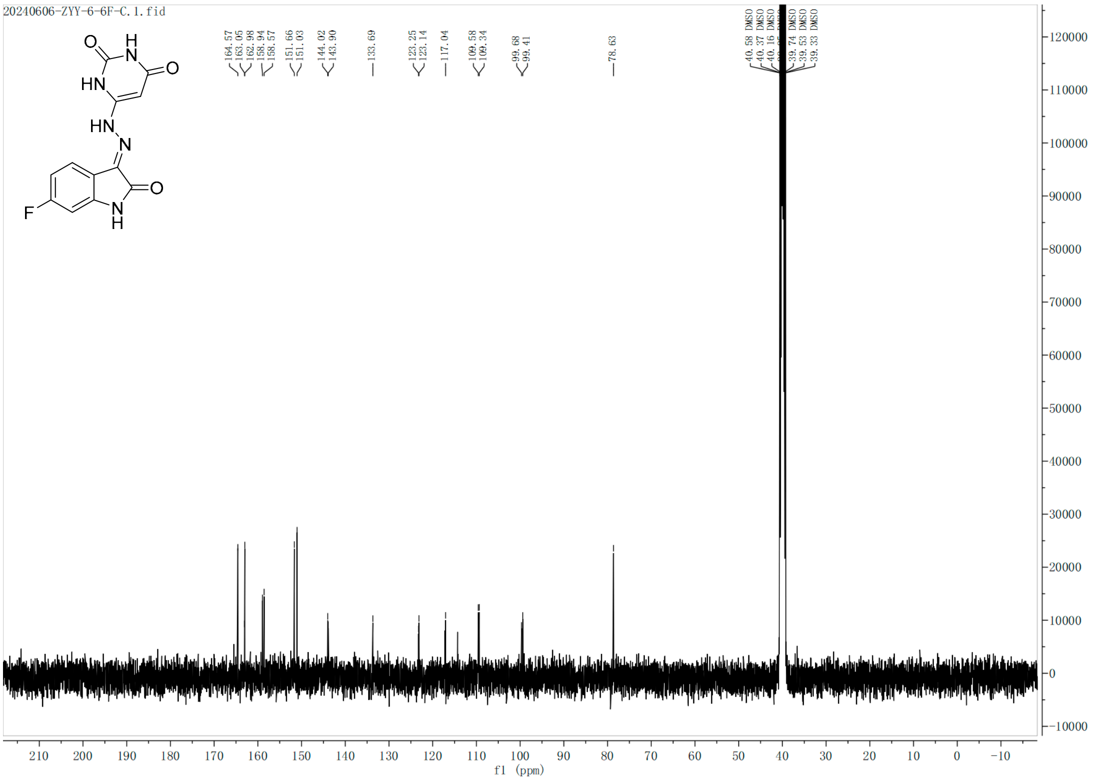

^{13}C NMR spectrum of **6g**.

Display Report

Analysis Info Acquisition Date 1/22/2024 10:27:30 AM

Sample Name 20240118-ZYY-6-6F-NEG
Comment

Acquisition Parameter

Source Type	ESI	Ion Polarity	Negative	Set Nebulizer	2.0 Bar
Focus	Active	Set Capillary	2800 V	Set Dry Heater	200 °C
Scan Begin	50 m/z	Set End Plate Offset	-500 V	Set Dry Gas	8.0 l/min
Scan End	1500 m/z	Set Charging Voltage	2000 V	Set Divert Valve	Waste
		Set Corona	0 nA	Set APCI Heater	0 °C

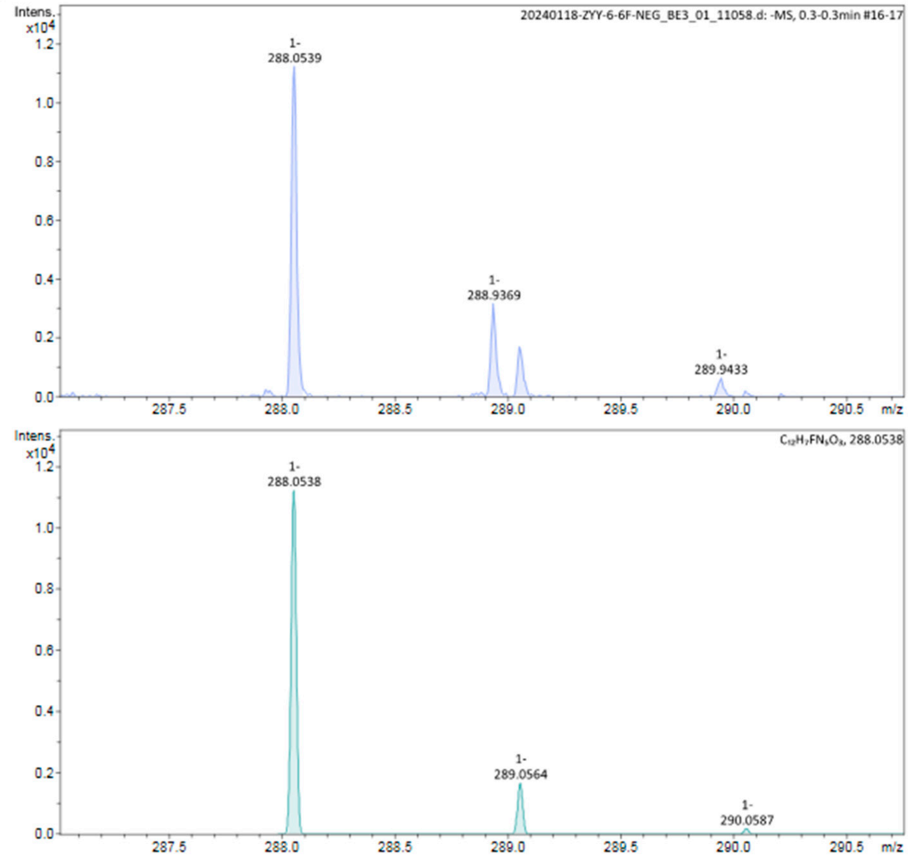

20240118-ZYY-6-6F-NEG_BE3_01_11058.d

printed: 1/22/2024 10:36:33 AM Page 1 of 1

HRMS spectrum of **6g**.

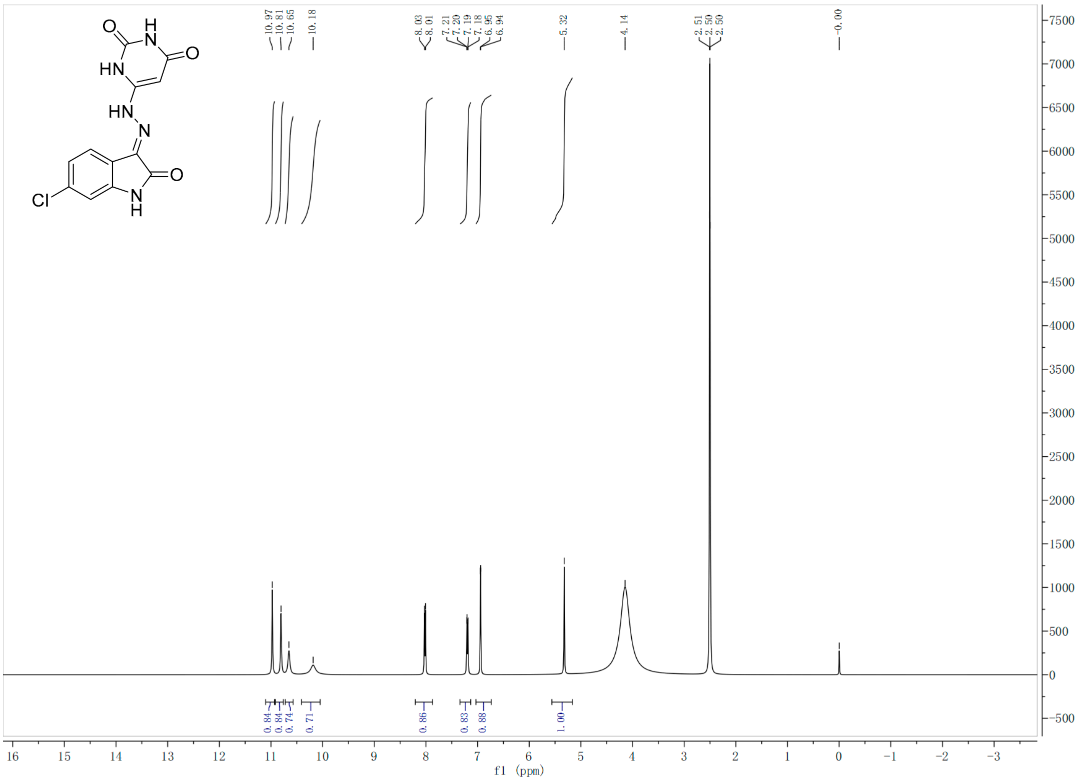

^{1}H NMR spectrum of **6h**.

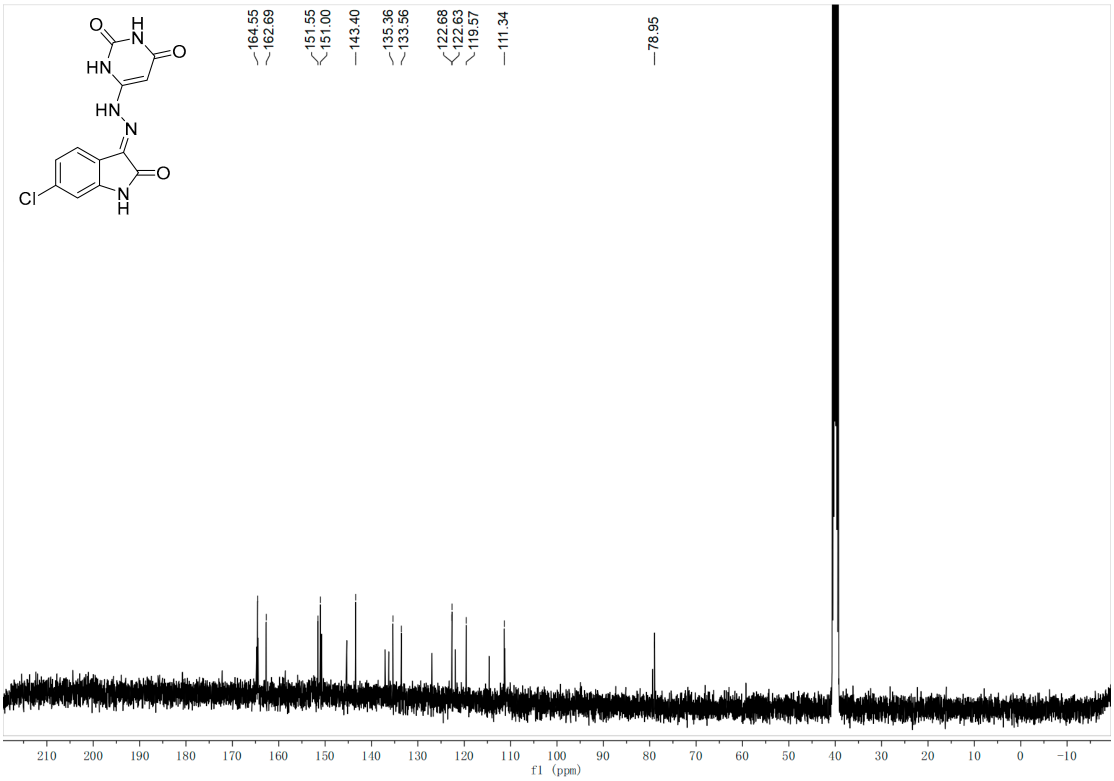

^{13}C NMR spectrum of **6h**.

Display Report

Analysis Info

Acquisition Date 1/22/2024 10:21:34 AM

Sample Name 20240118-ZYY-6-6Cl-NEG
Comment

Acquisition Parameter

Source Type	ESI	Ion Polarity	Negative	Set Nebulizer	2.0 Bar
Focus	Active	Set Capillary	2800 V	Set Dry Heater	200 °C
Scan Begin	50 m/z	Set End Plate Offset	-500 V	Set Dry Gas	8.0 l/min
Scan End	1500 m/z	Set Charging Voltage	2000 V	Set Divert Valve	Waste
		Set Corona	0 nA	Set APCI Heater	0 °C

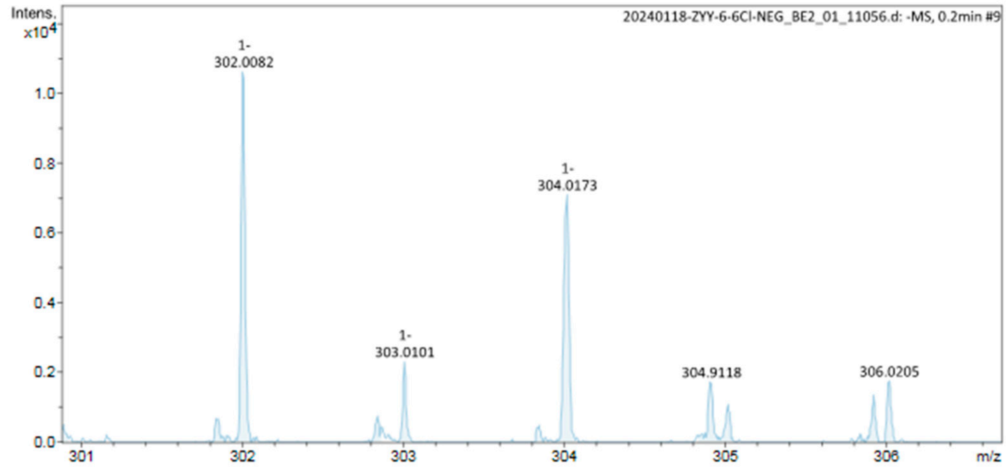

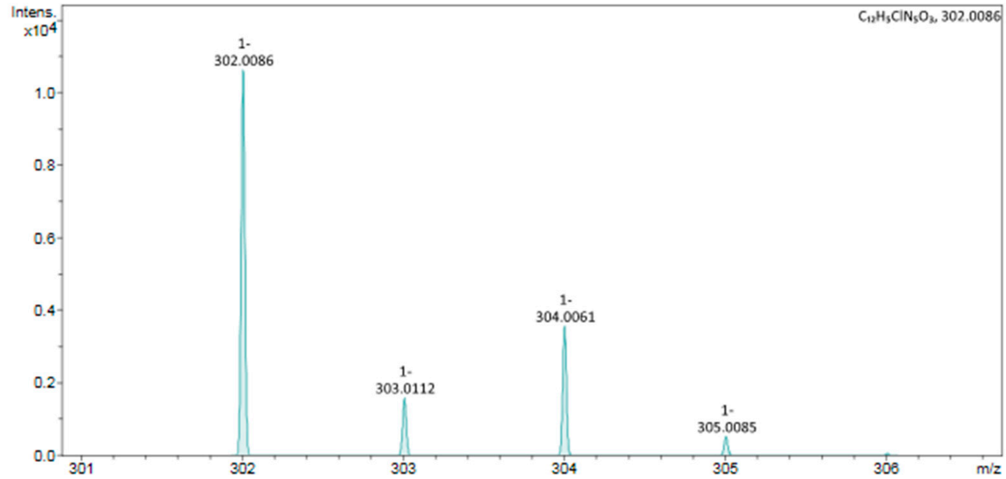

20240118-ZYY-6-6Cl-NEG_BE2_01_11056.d

printed: 1/22/2024 10:40:59 AM Page 1 of 1

HRMS spectrum of **6h**.

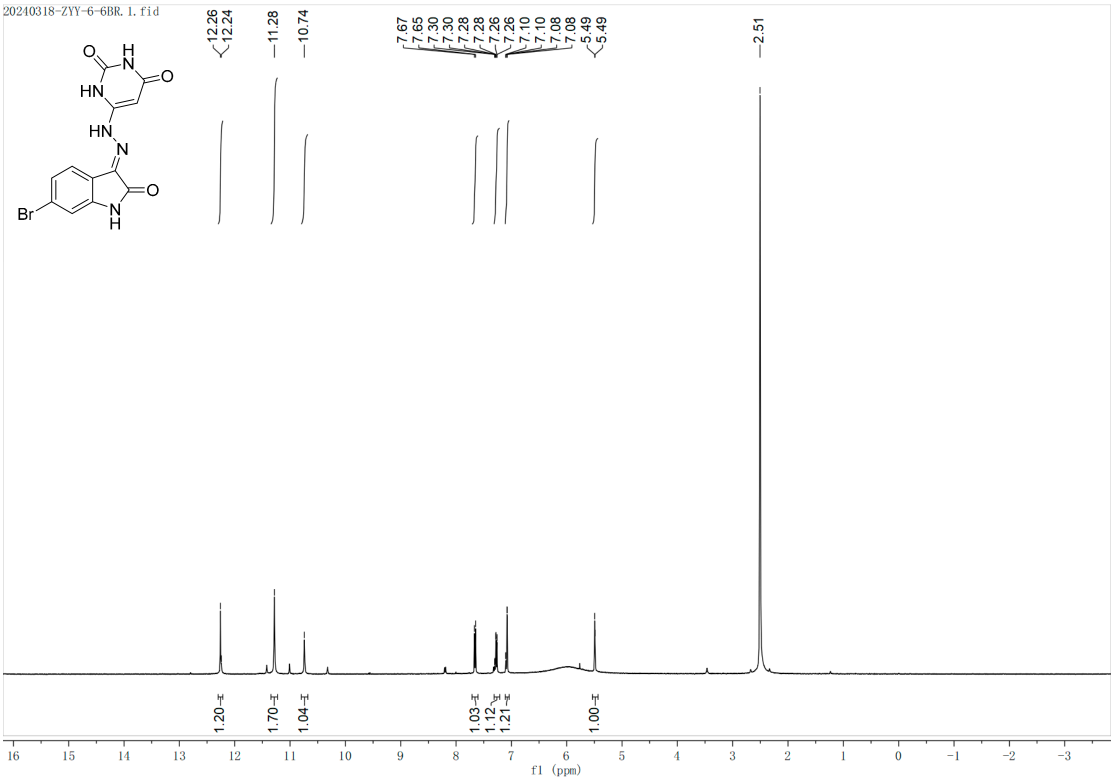

^{1}H NMR spectrum of **6i**.

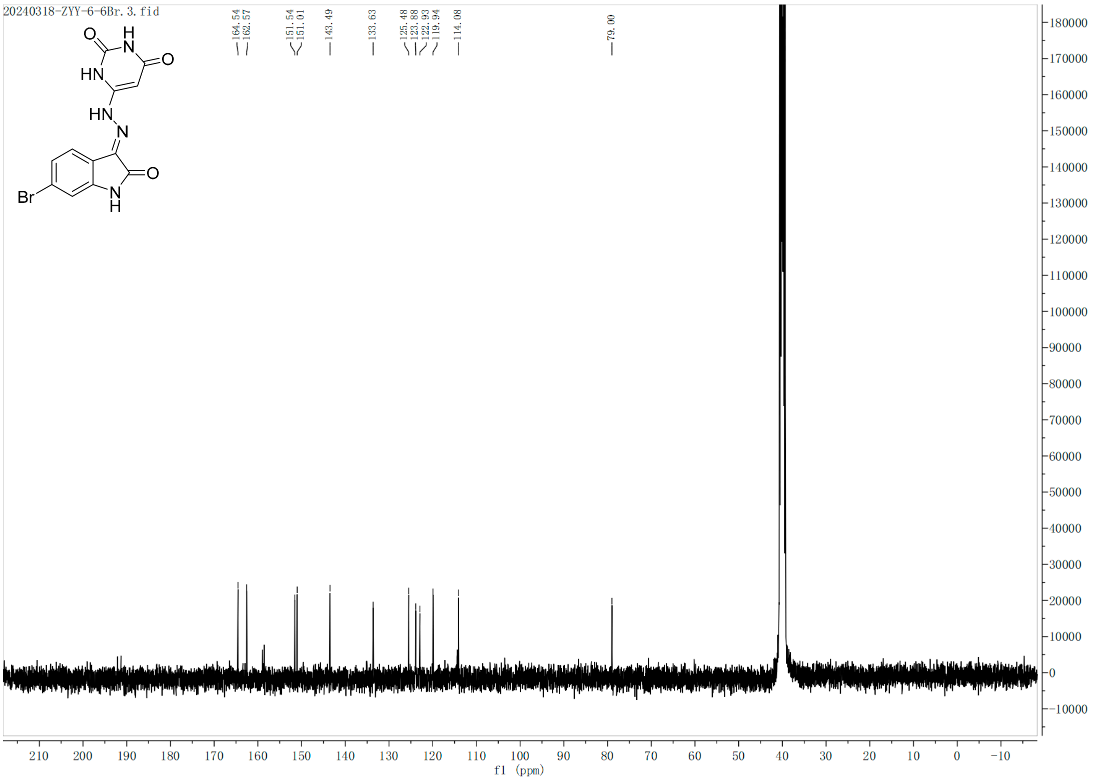

^{13}C NMR spectrum of **6i**.

Display Report

Analysis Info Acquisition Date 1/22/2024 10:15:16 AM

Sample Name 20240118-ZYY-6NH2-6BR-NEG
Comment

Acquisition Parameter

Source Type	ESI	Ion Polarity	Negative	Set Nebulizer	2.0 Bar
Focus	Active	Set Capillary	2800 V	Set Dry Heater	200 °C
Scan Begin	50 m/z	Set End Plate Offset	-500 V	Set Dry Gas	8.0 l/min
Scan End	1500 m/z	Set Charging Voltage	2000 V	Set Divert Valve	Waste
		Set Corona	0 nA	Set APCI Heater	0 °C

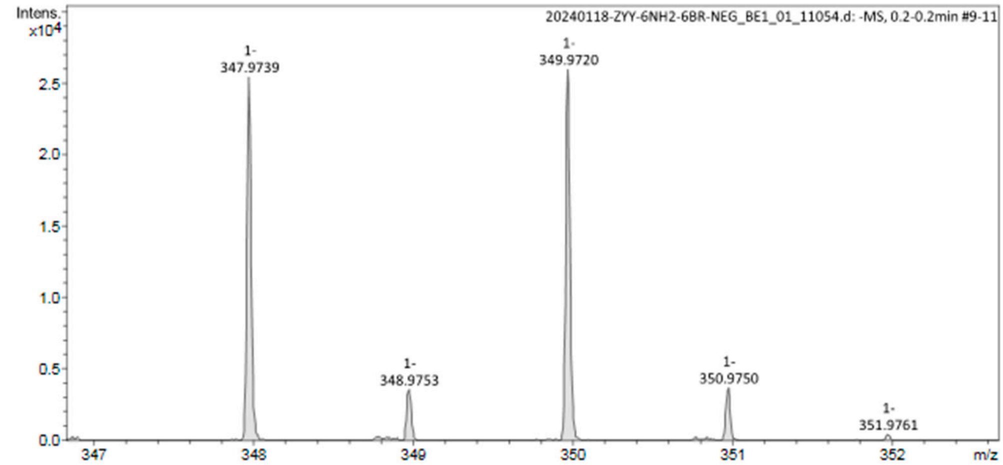

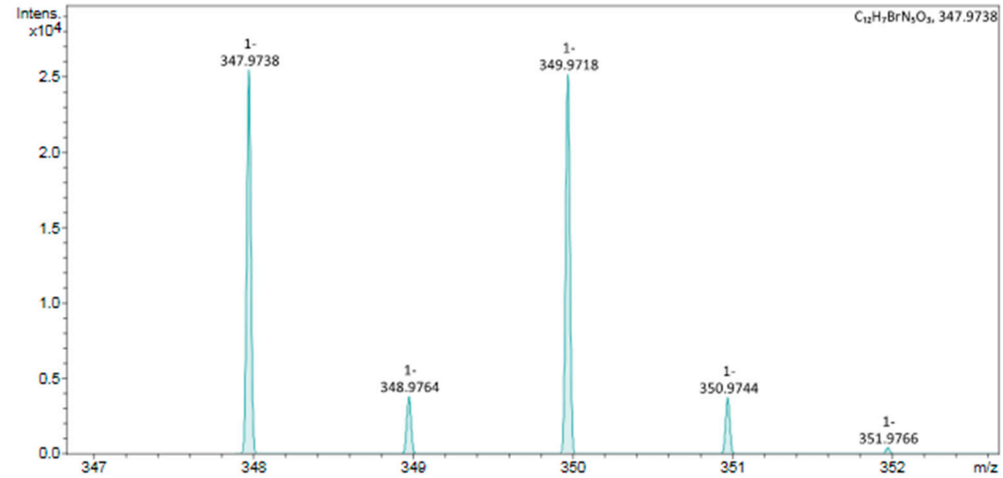

20240118-ZYY-6NH2-6BR-NEG_BE1_01_11054.d

printed: 1/22/2024 10:37:44 AM Page 1 of 1

HRMS spectrum of **6i**.

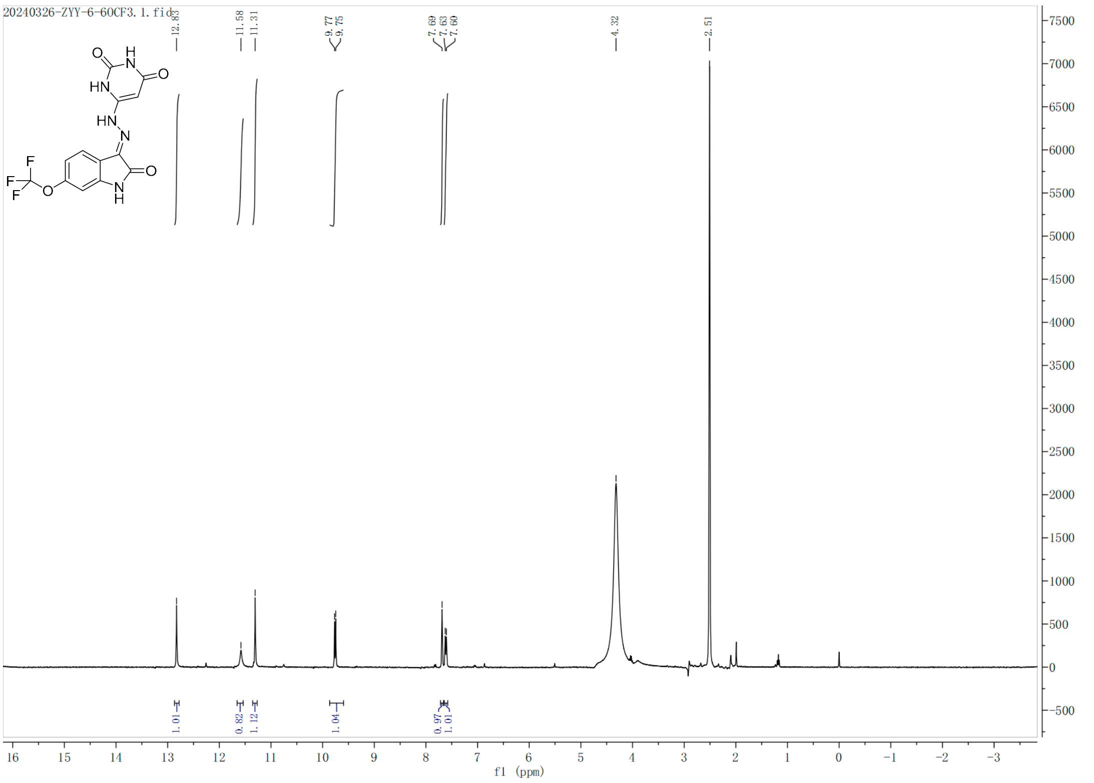

^{1}H NMR spectrum of **6j**.

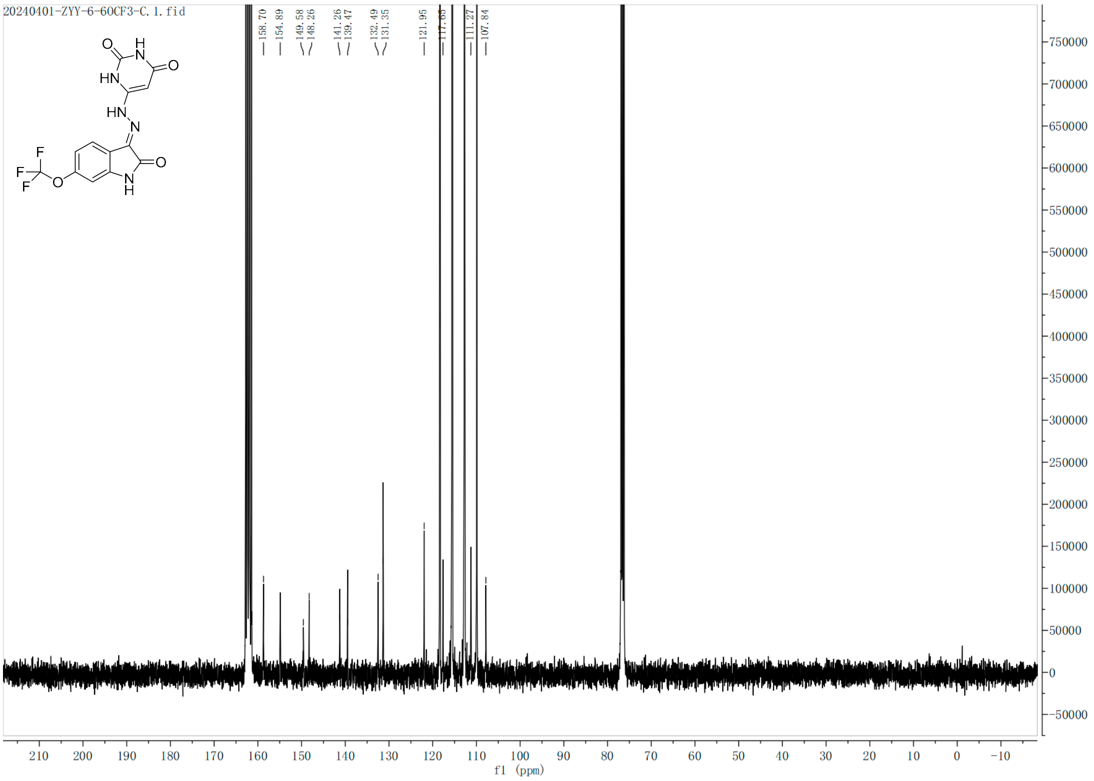

^{13}C NMR spectrum of **6j**.

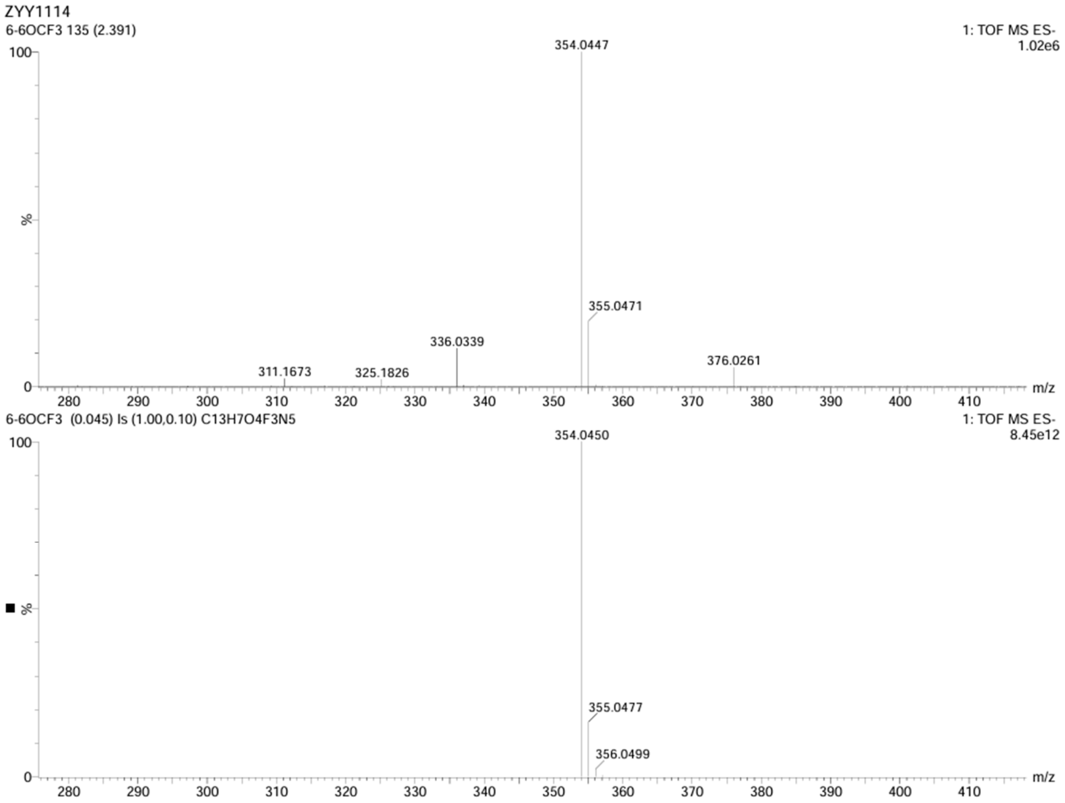

HRMS spectrum of **6j**.

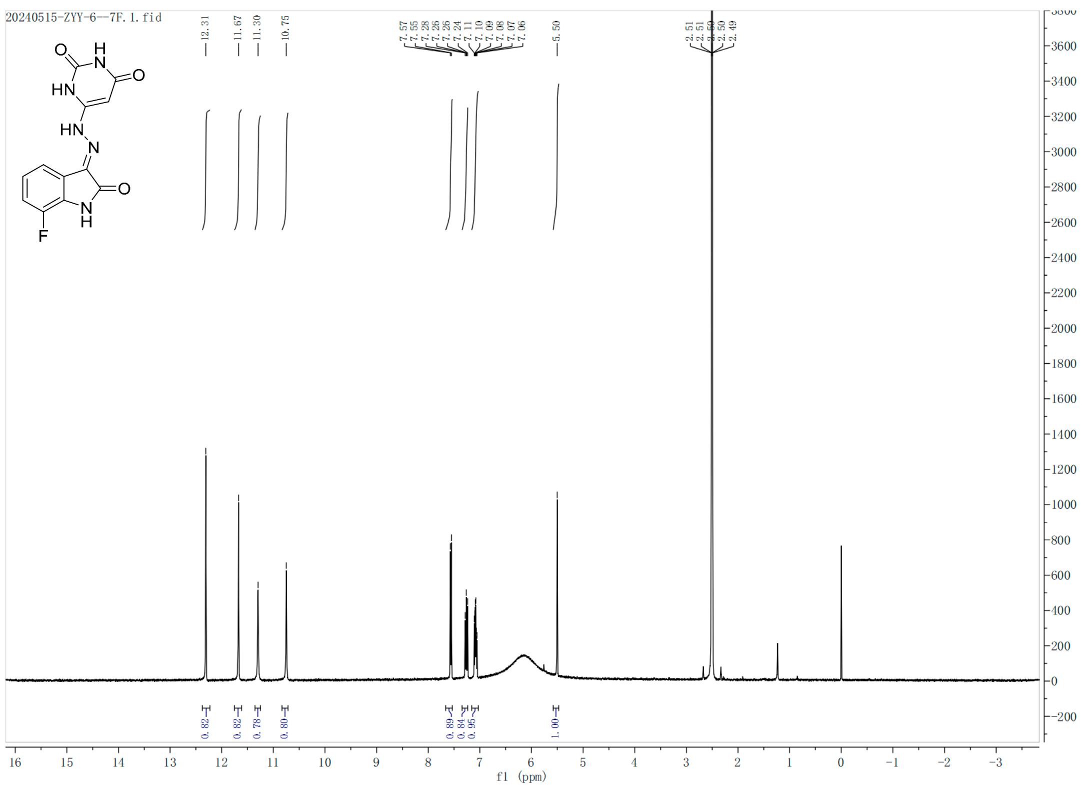

^{1}H NMR spectrum of **6k**.

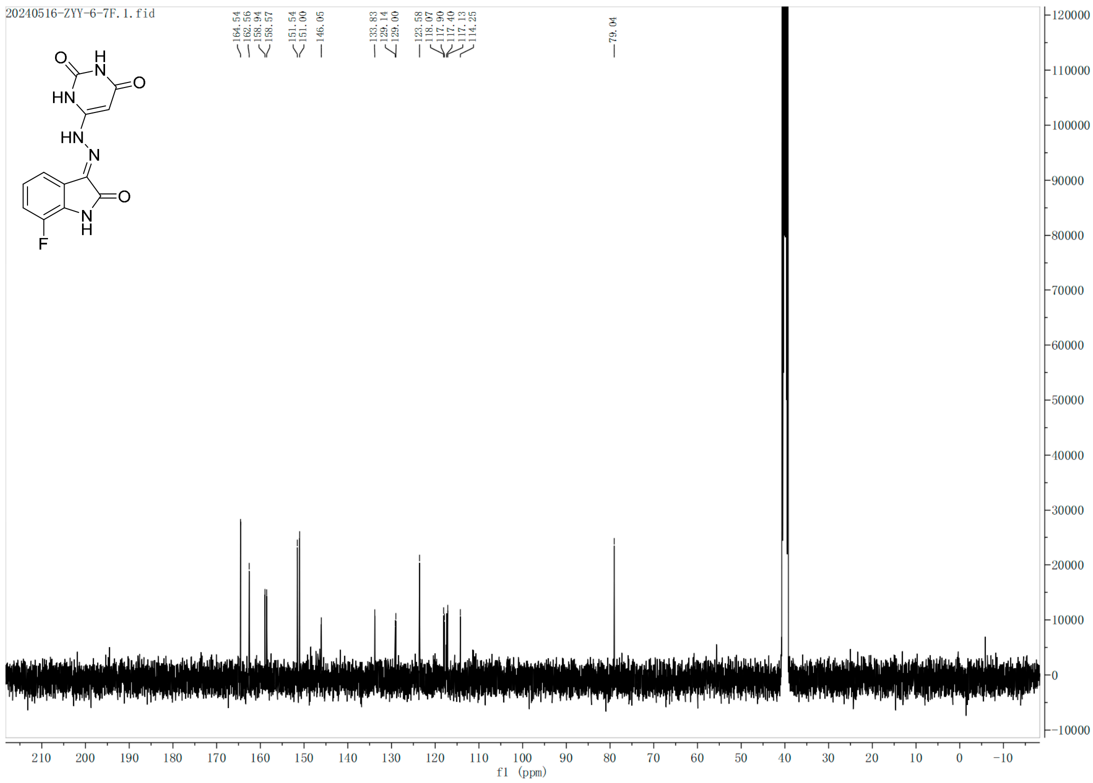

^{13}C NMR spectrum of **6k**.

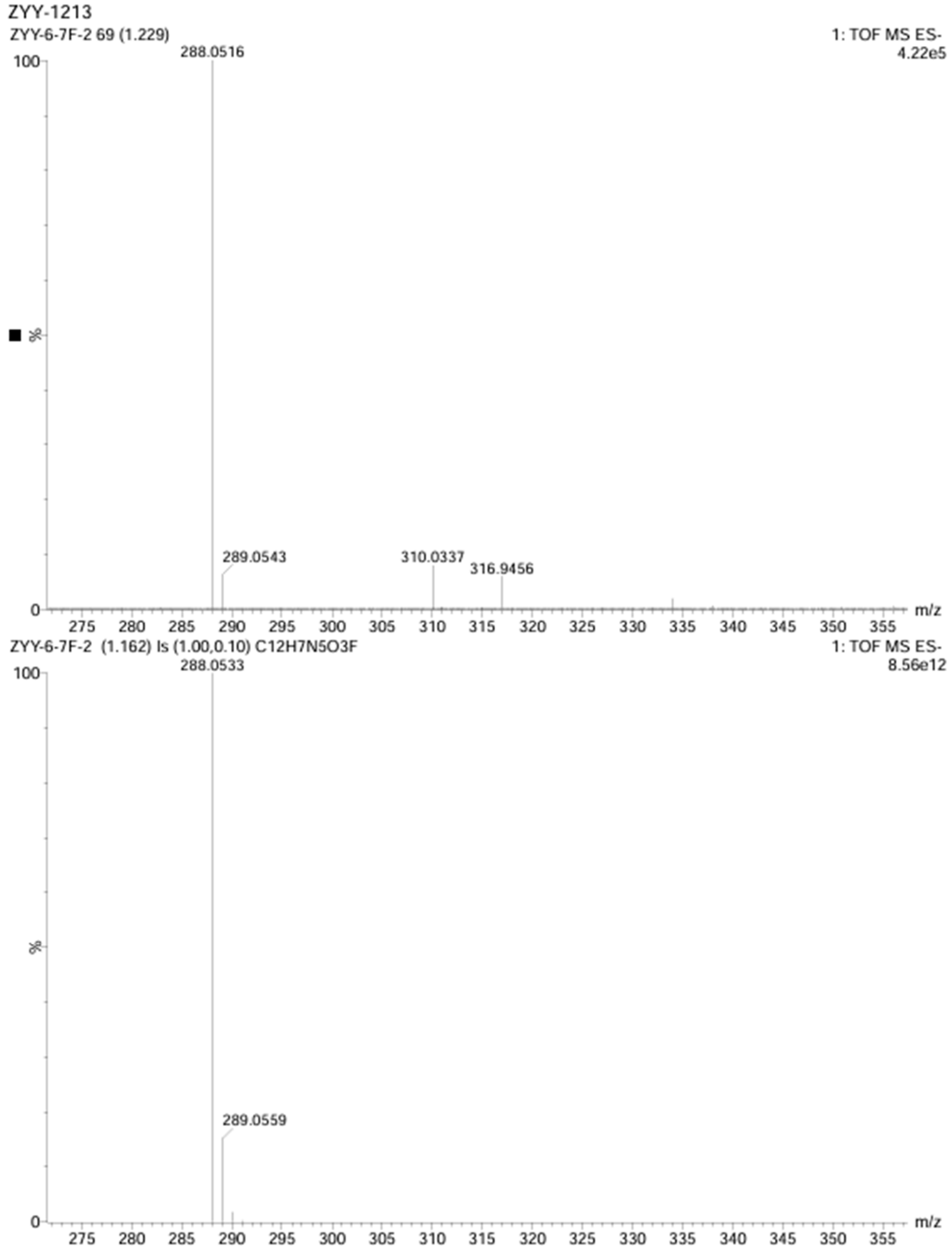

HRMS spectrum of **6k**.

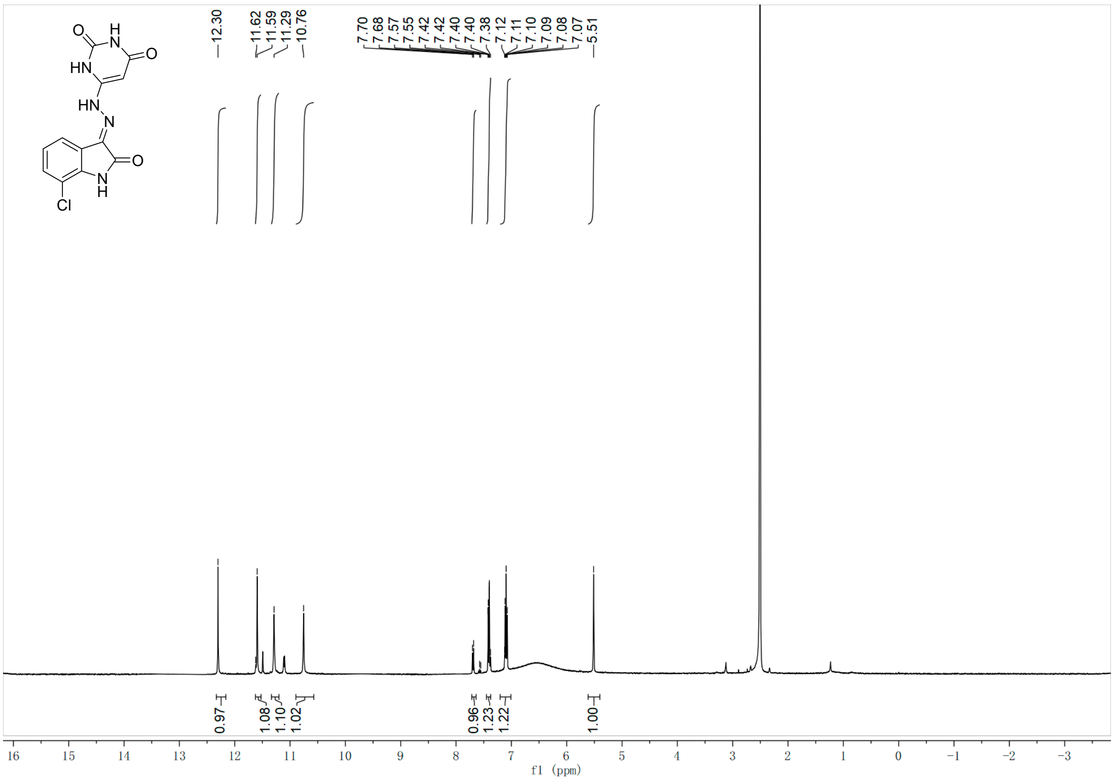

^{1}H NMR spectrum of **6l**.

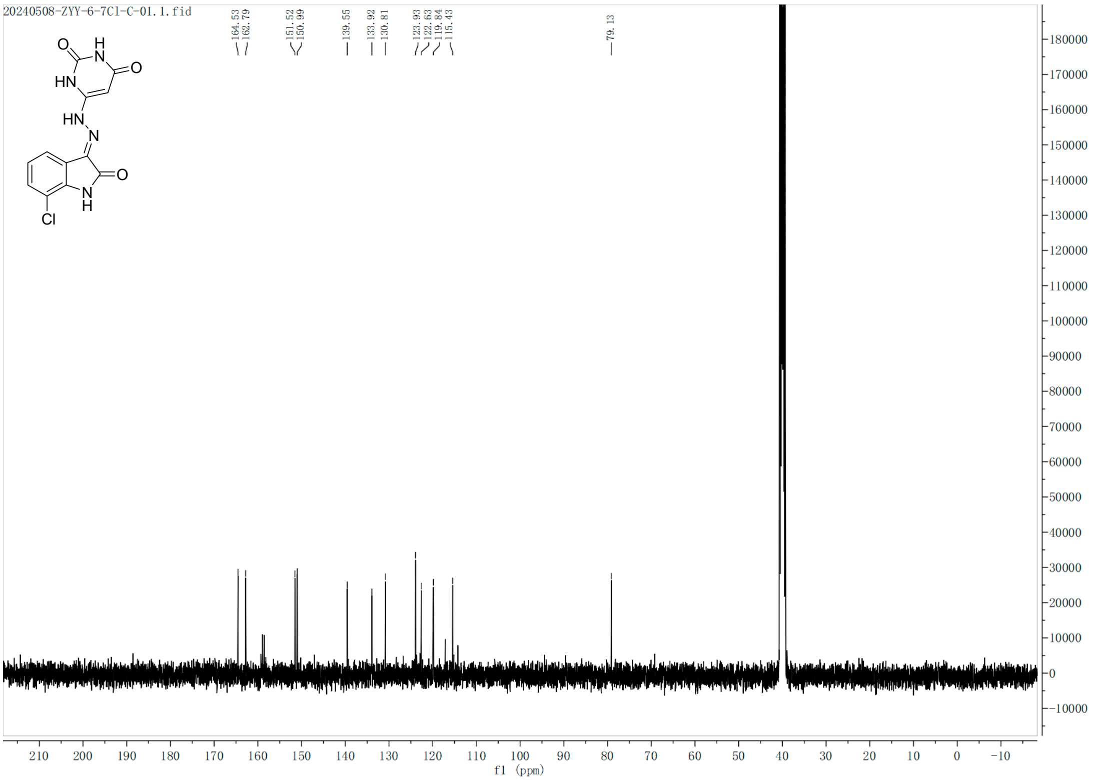

^{13}C NMR spectrum of **6l**.

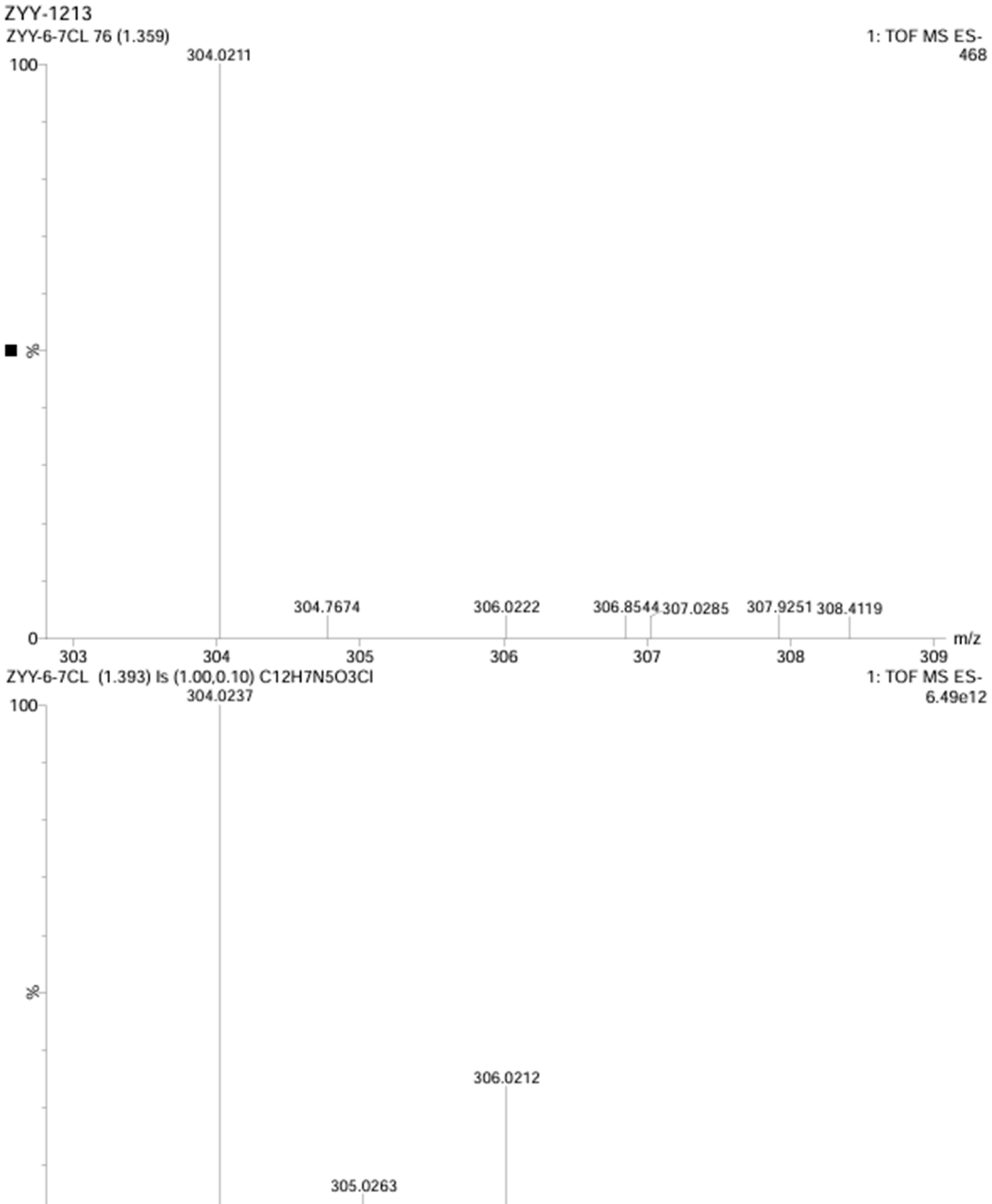

HRMS spectrum of **6l**.

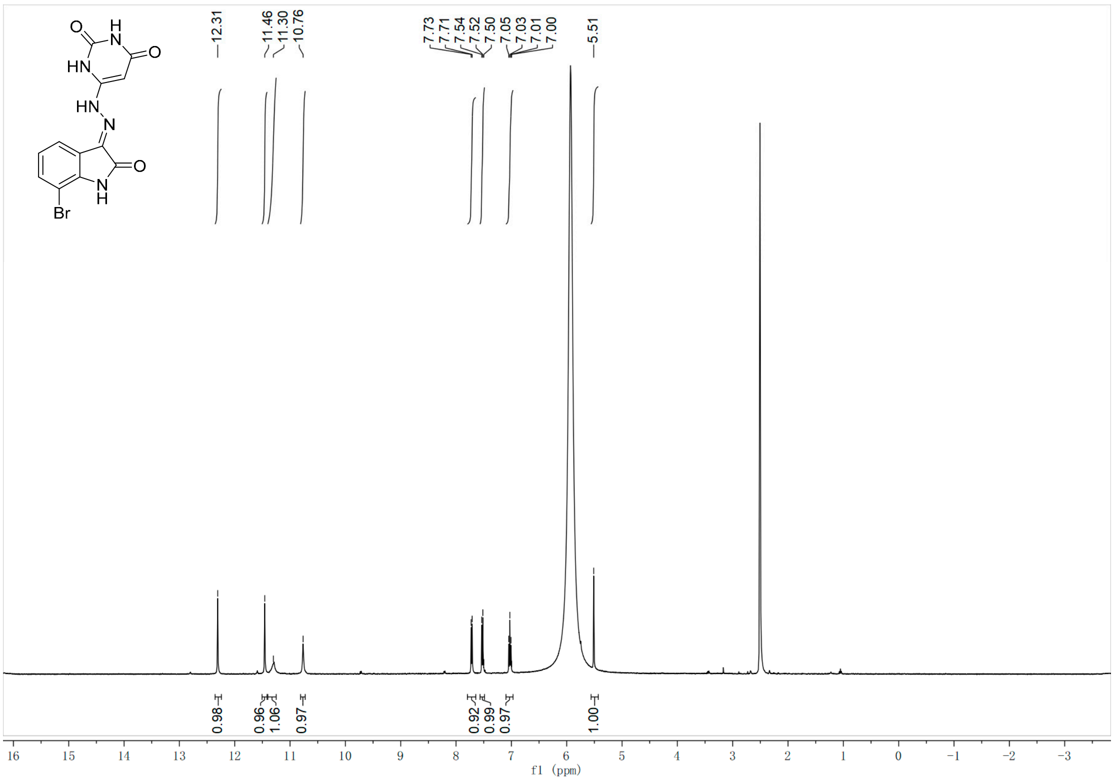

^{1}H NMR spectrum of **6m**.

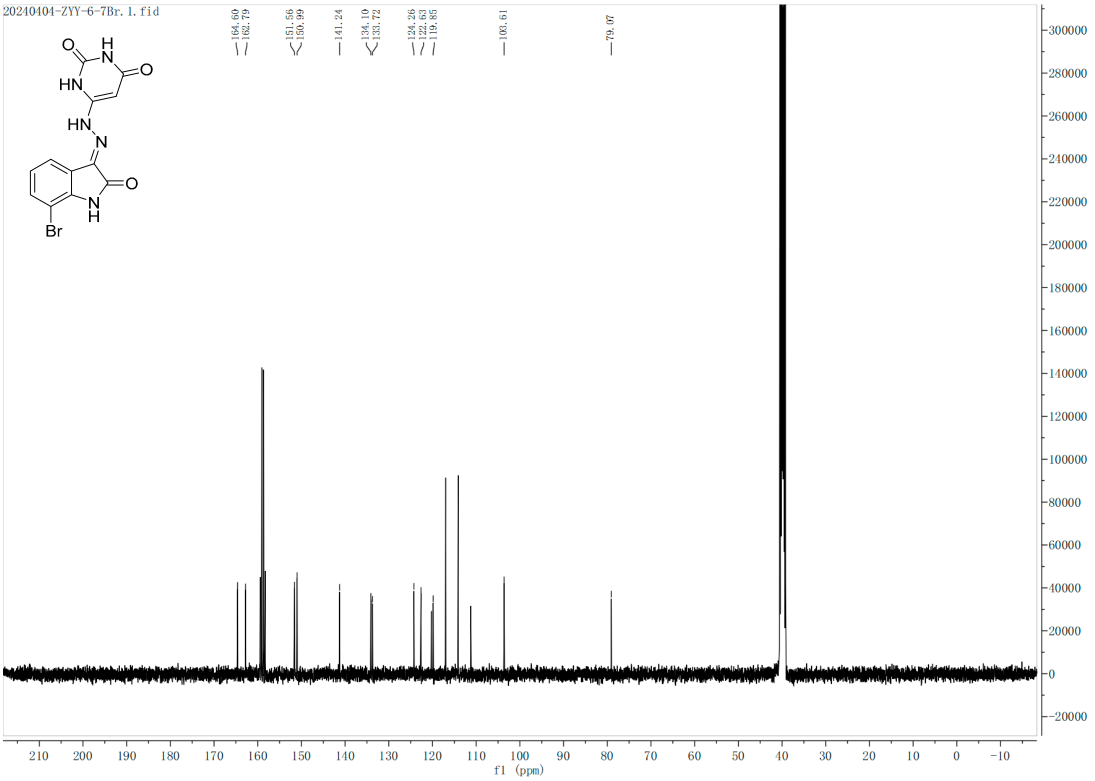

^{13}C NMR spectrum of **6m**.

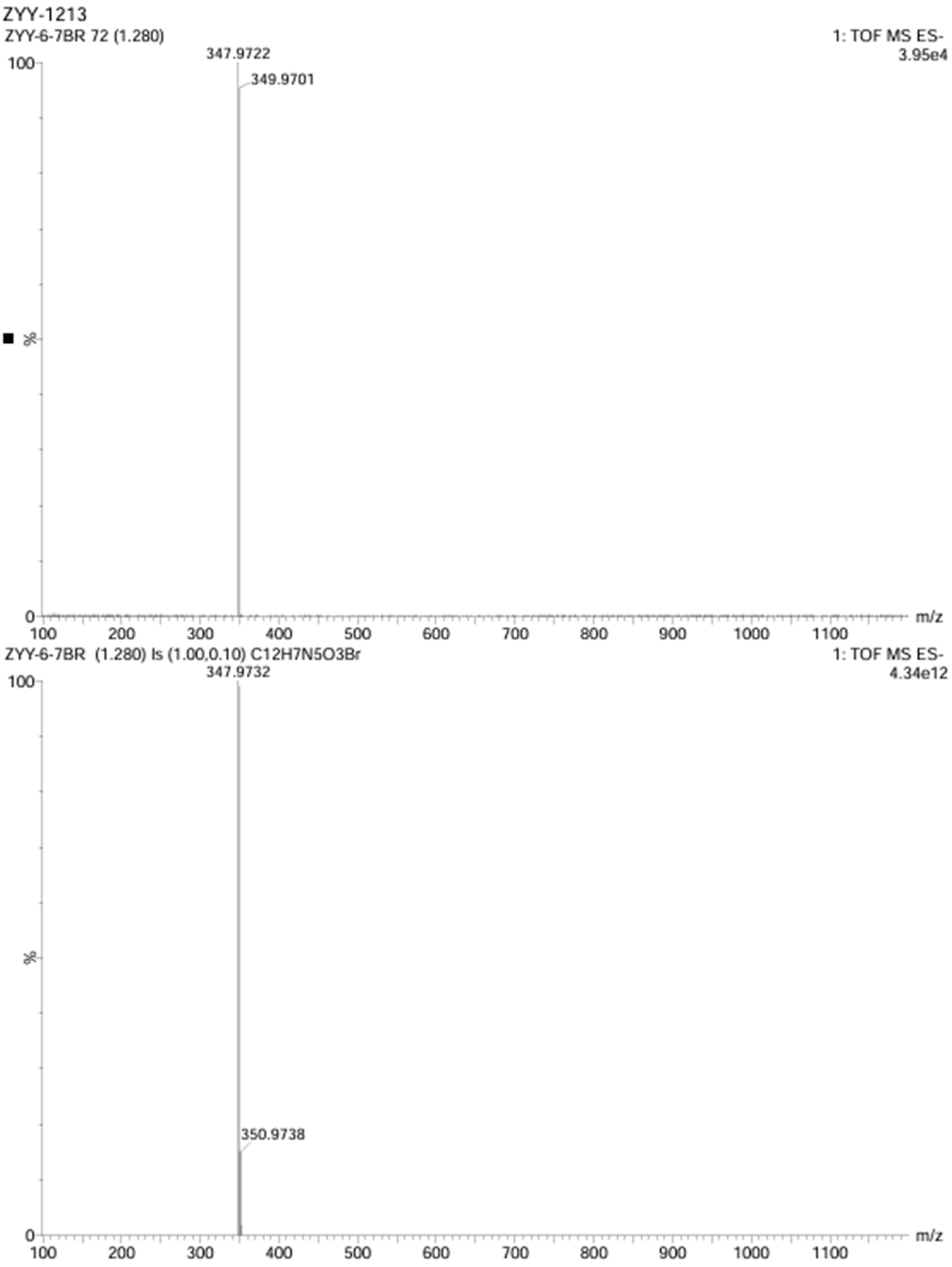

HRMS spectrum of **6m**.

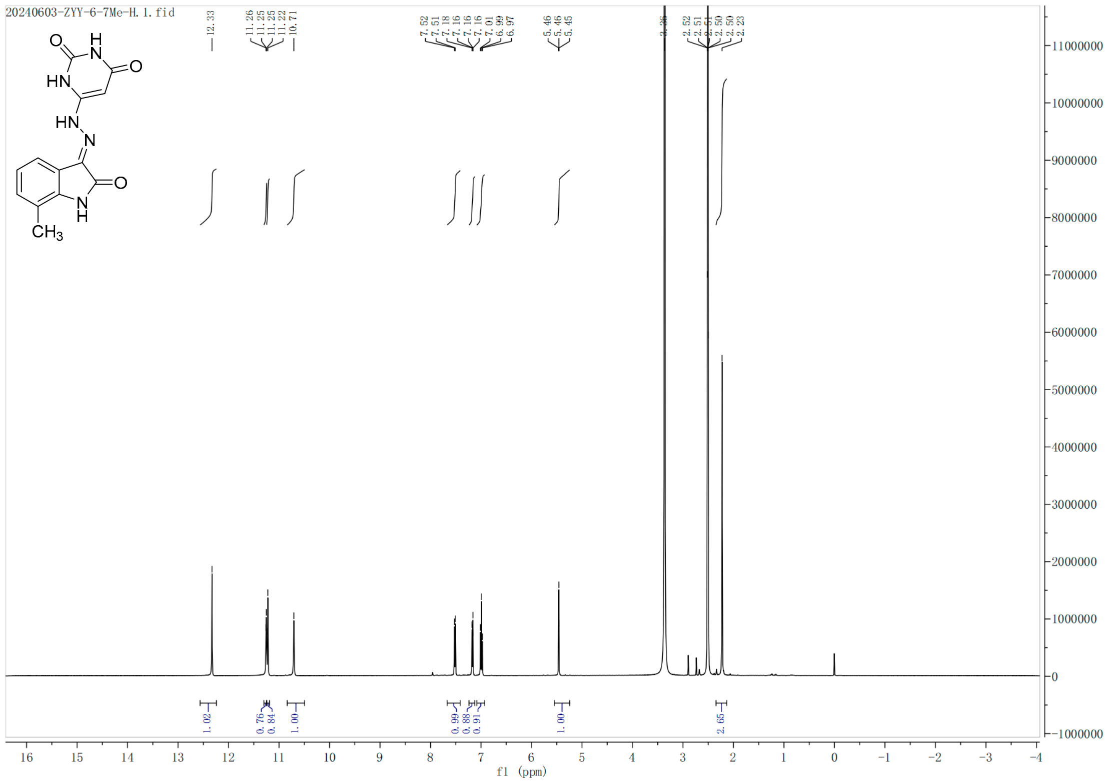

^{1}H NMR spectrum of **6n**.

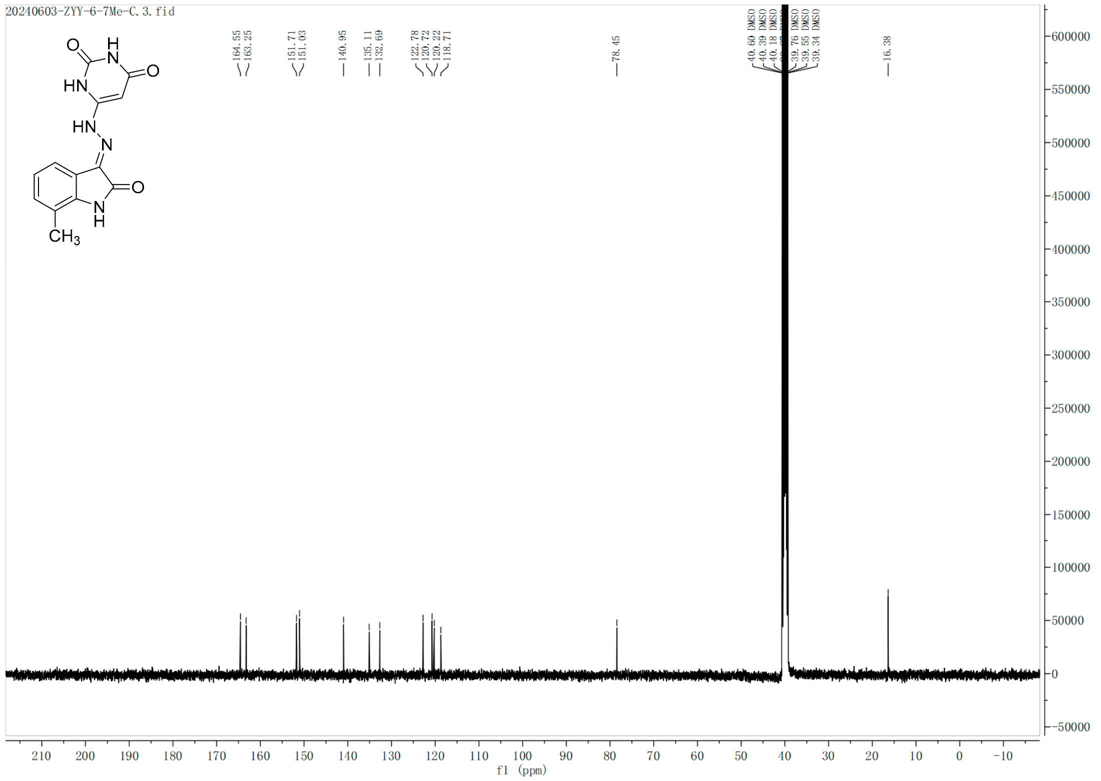

^{13}C NMR spectrum of **6n**.

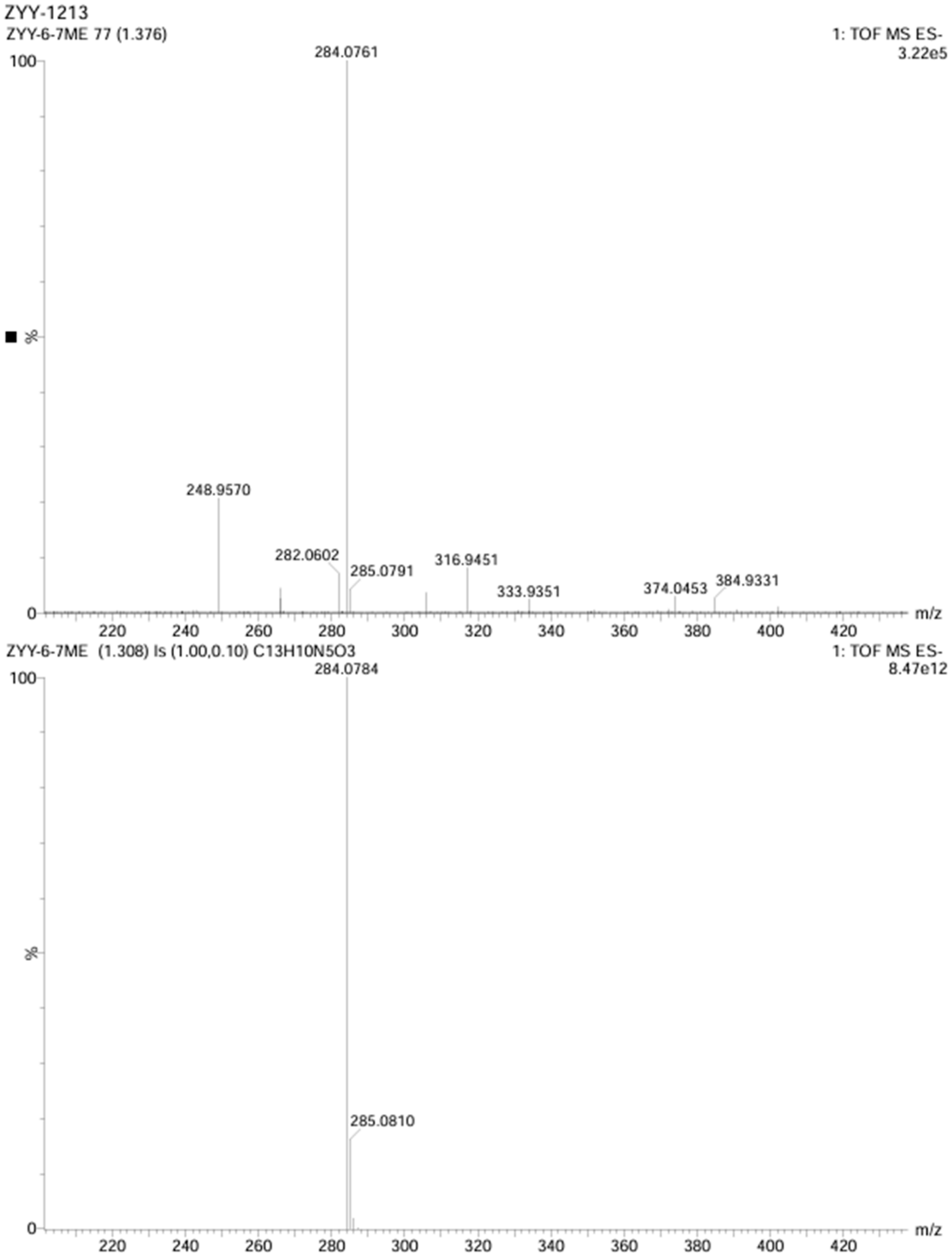

HRMS spectrum of **6n**.

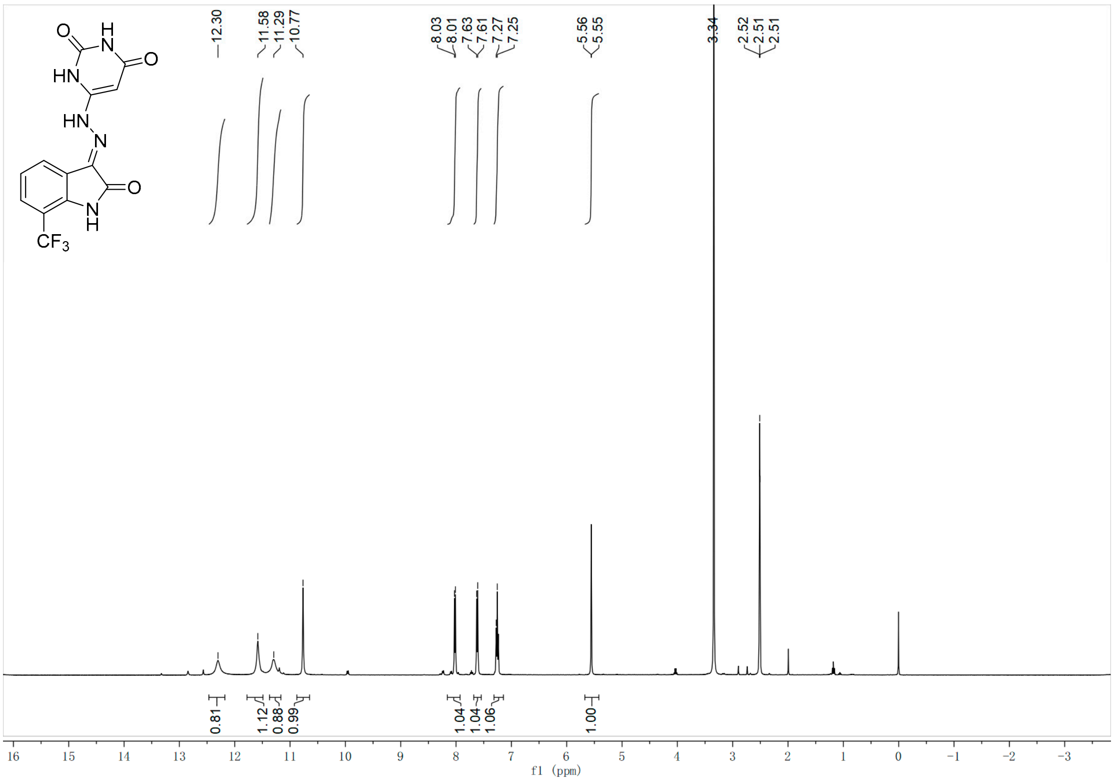

^{1}H NMR spectrum of **6o**.

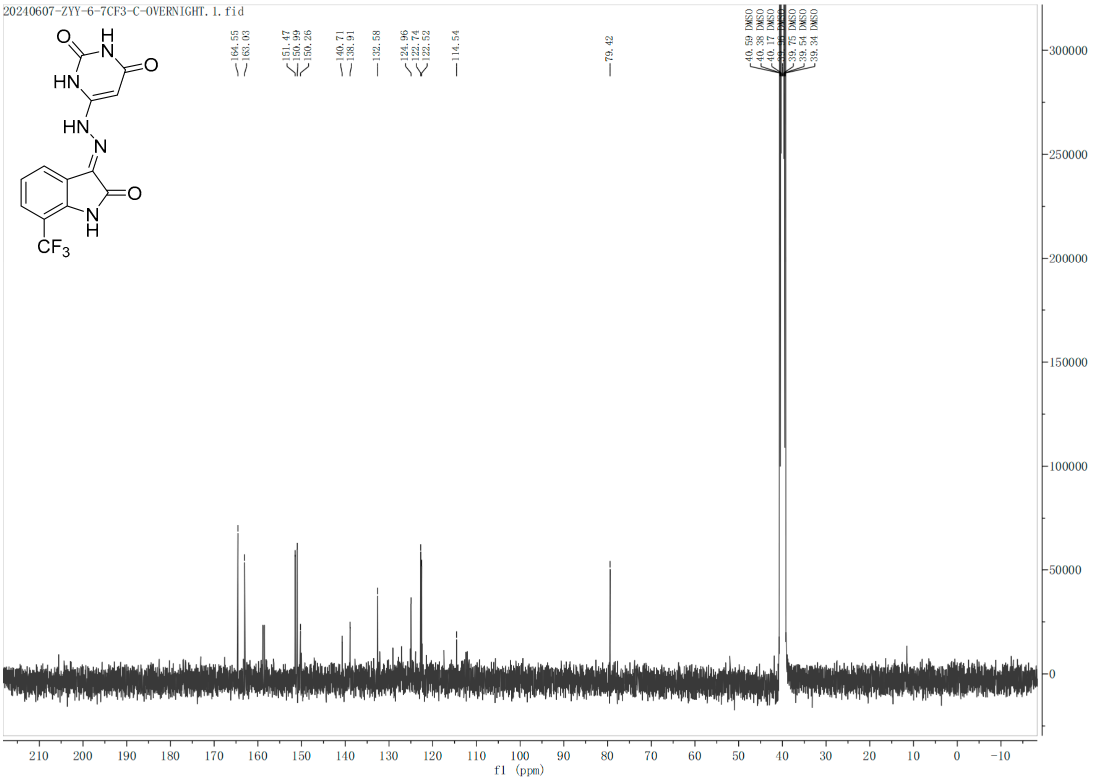

^{13}C NMR spectrum of **6o**.

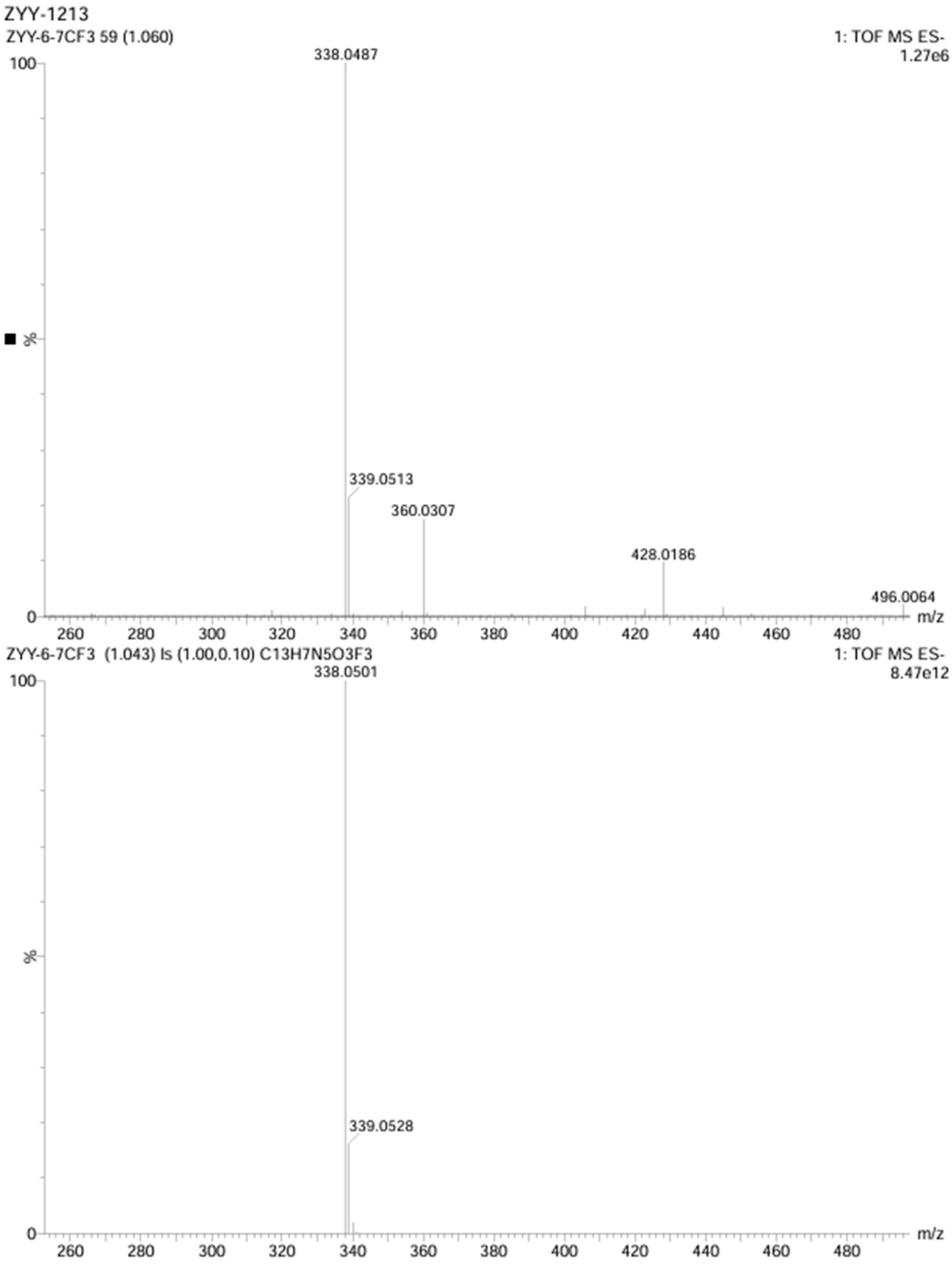

HRMS spectrum of **6o**.

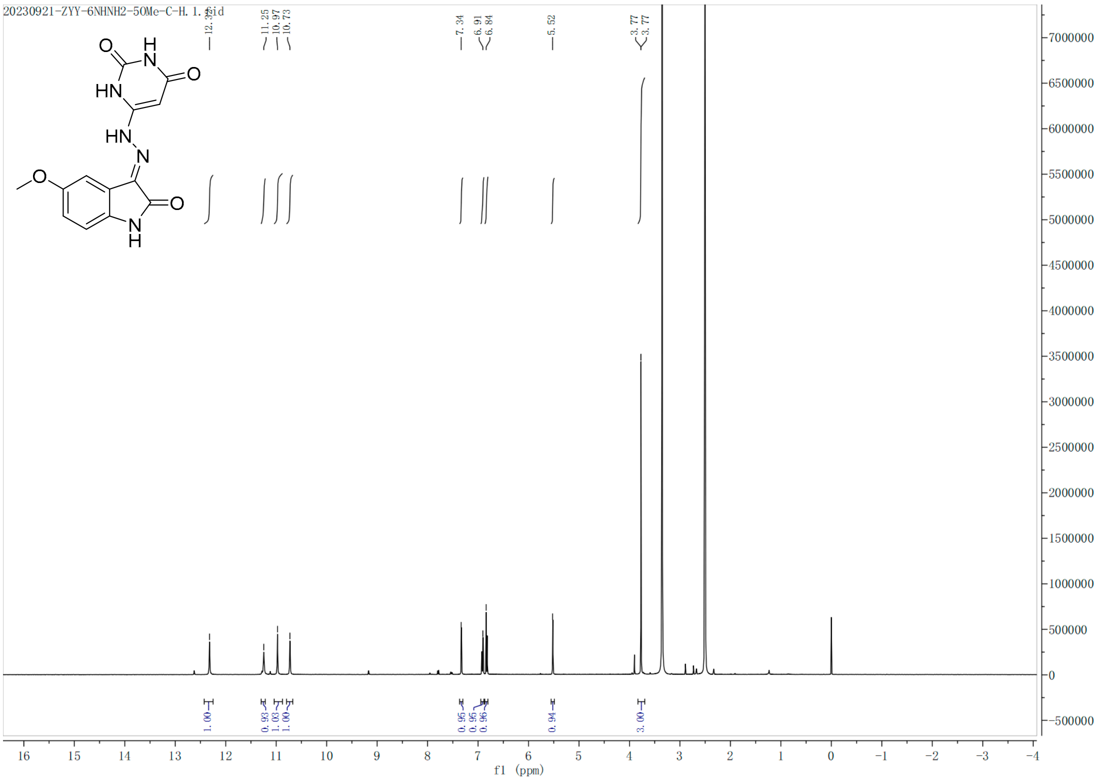

^{1}H NMR spectrum of **6p**.

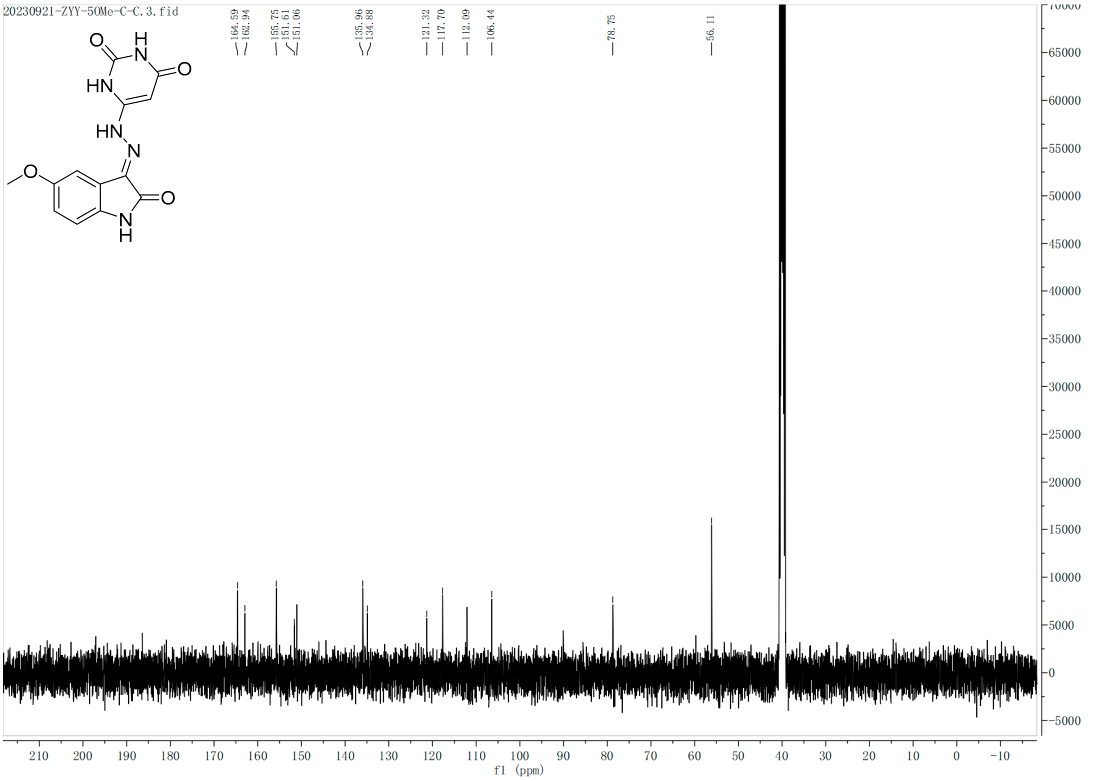

^{13}C NMR spectrum of **6p**.

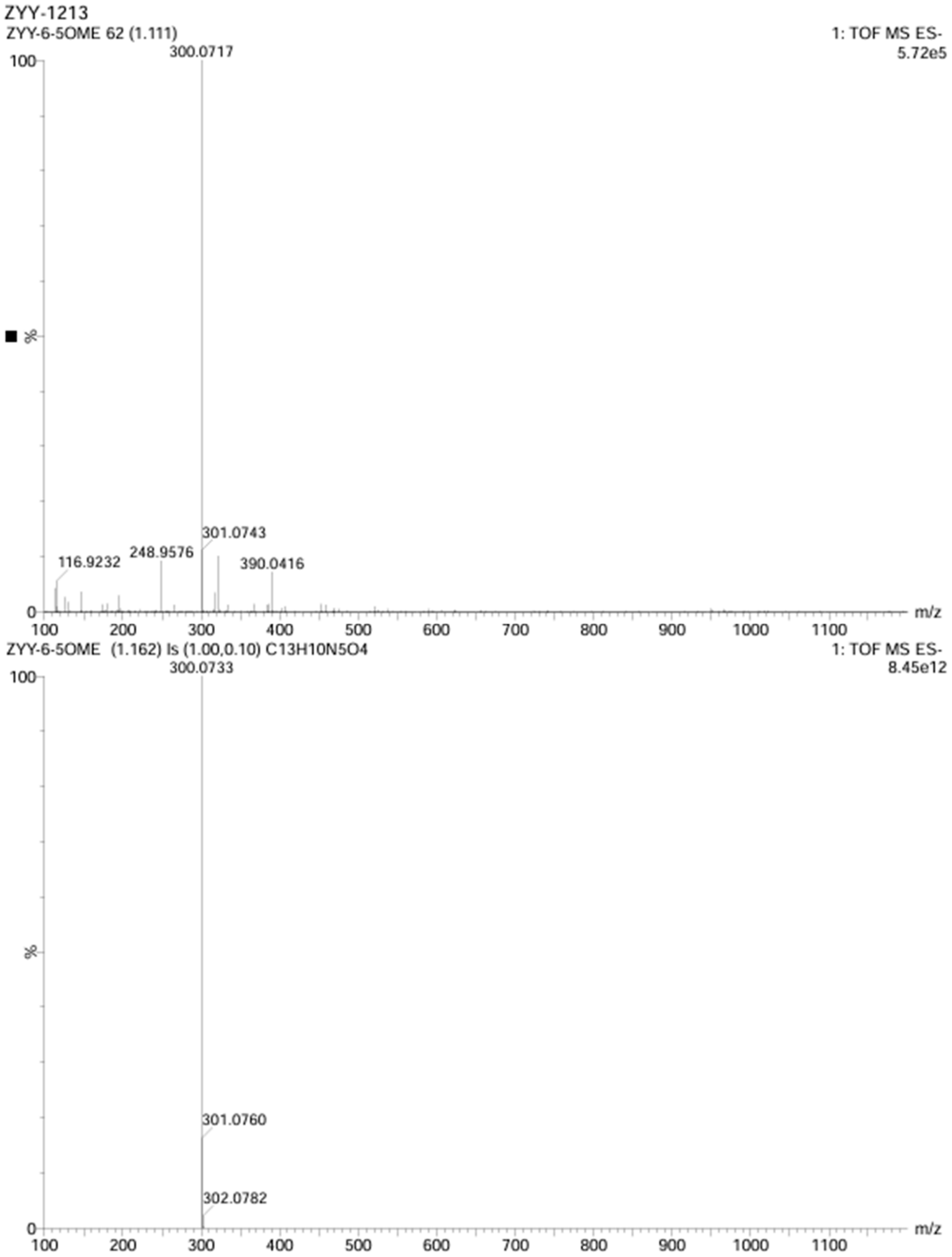

HRMS spectrum of **6p**.

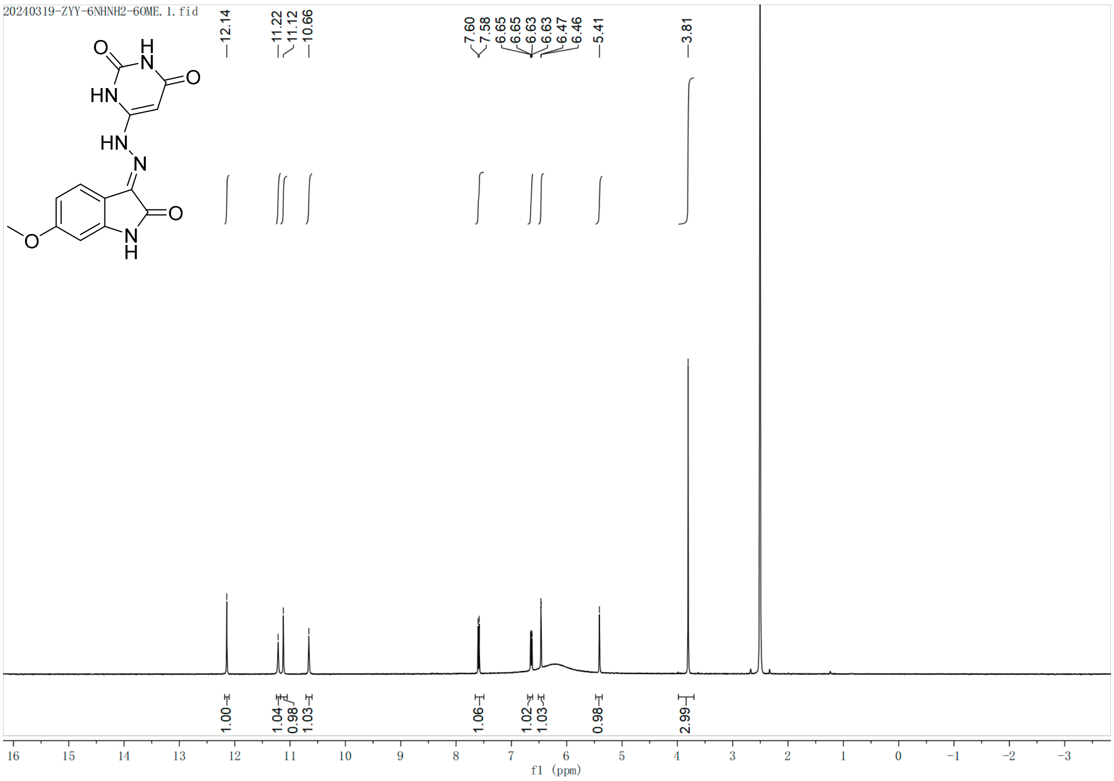

^{1}H NMR spectrum of **6q**.

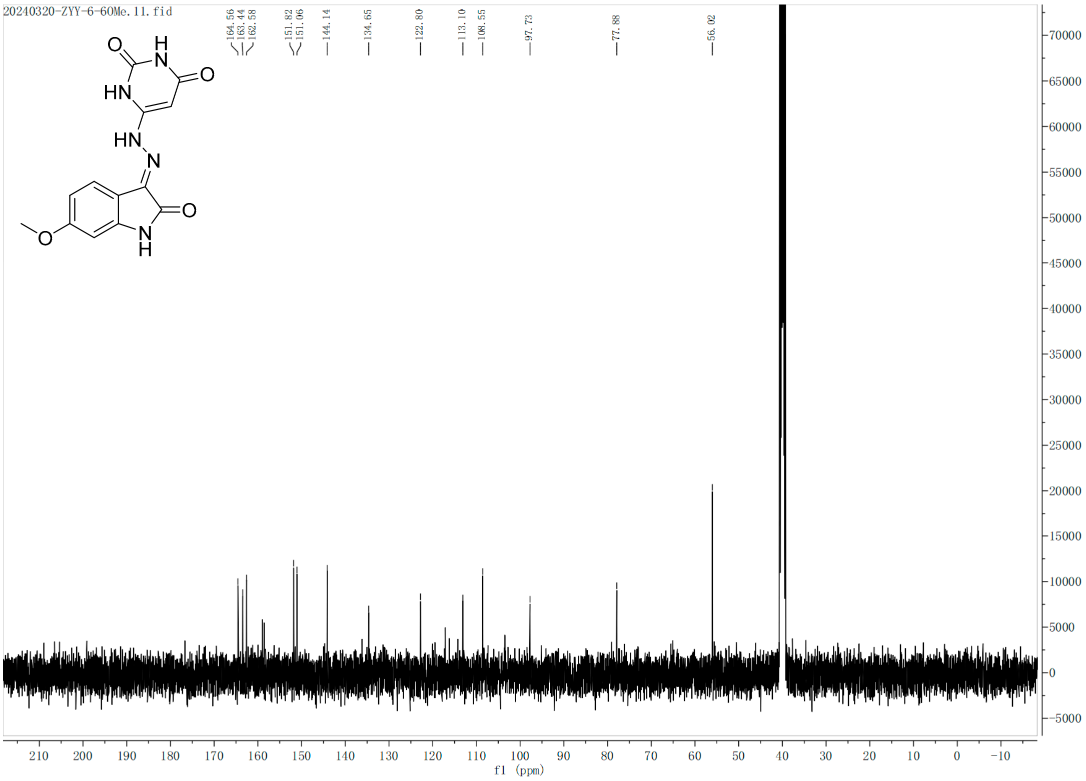

^{13}C NMR spectrum of **6q**.

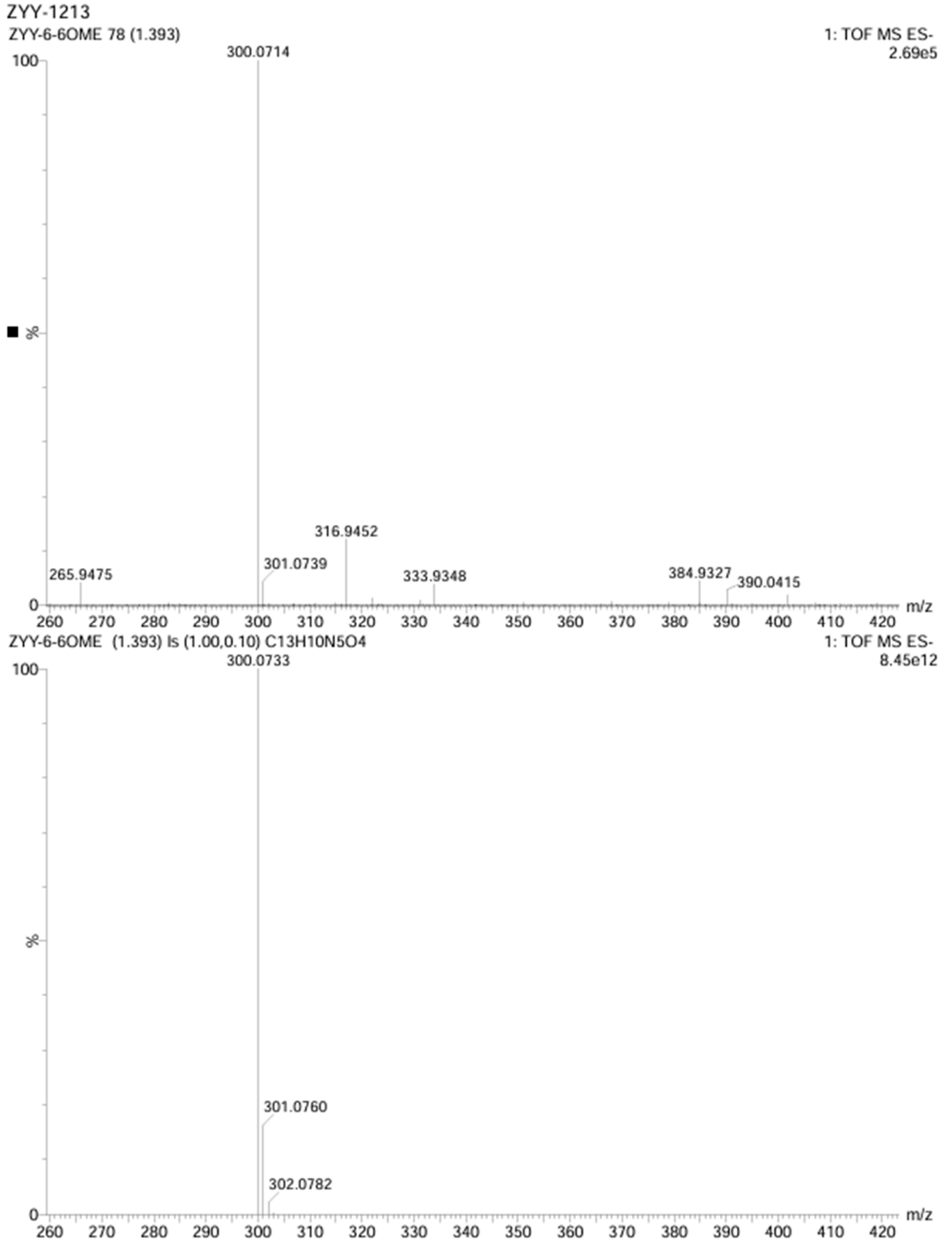

HRMS spectrum of **6q**.

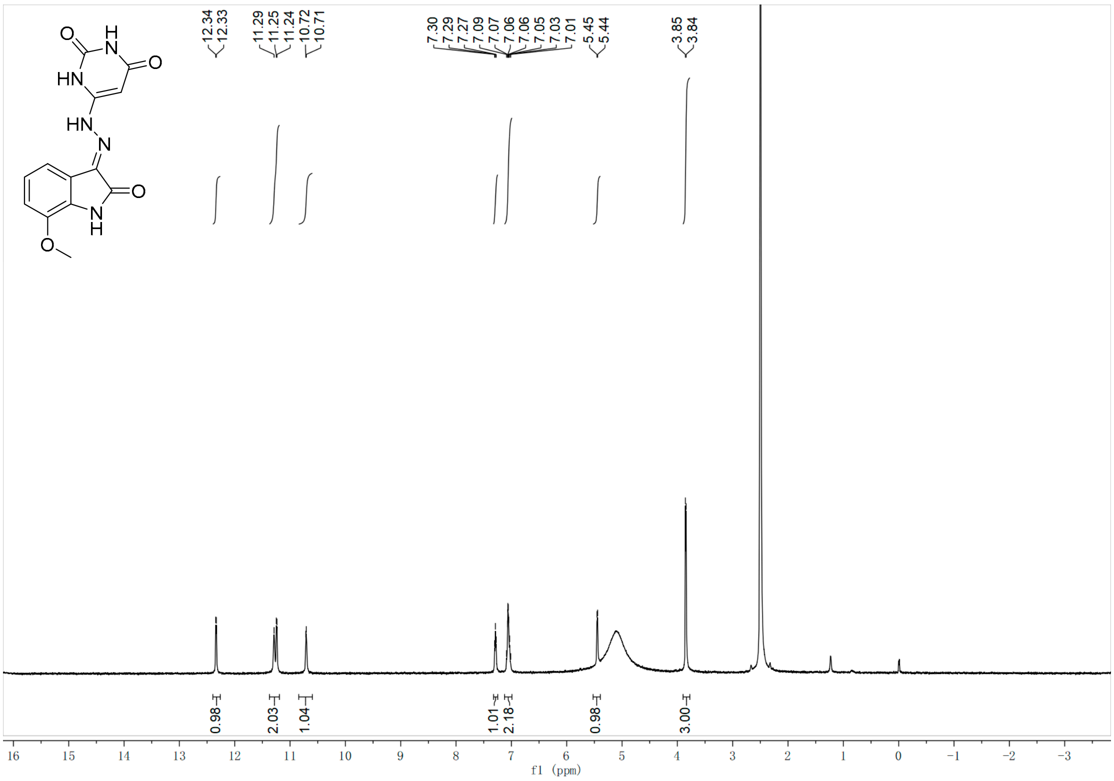

^{1}H NMR spectrum of **6r**.

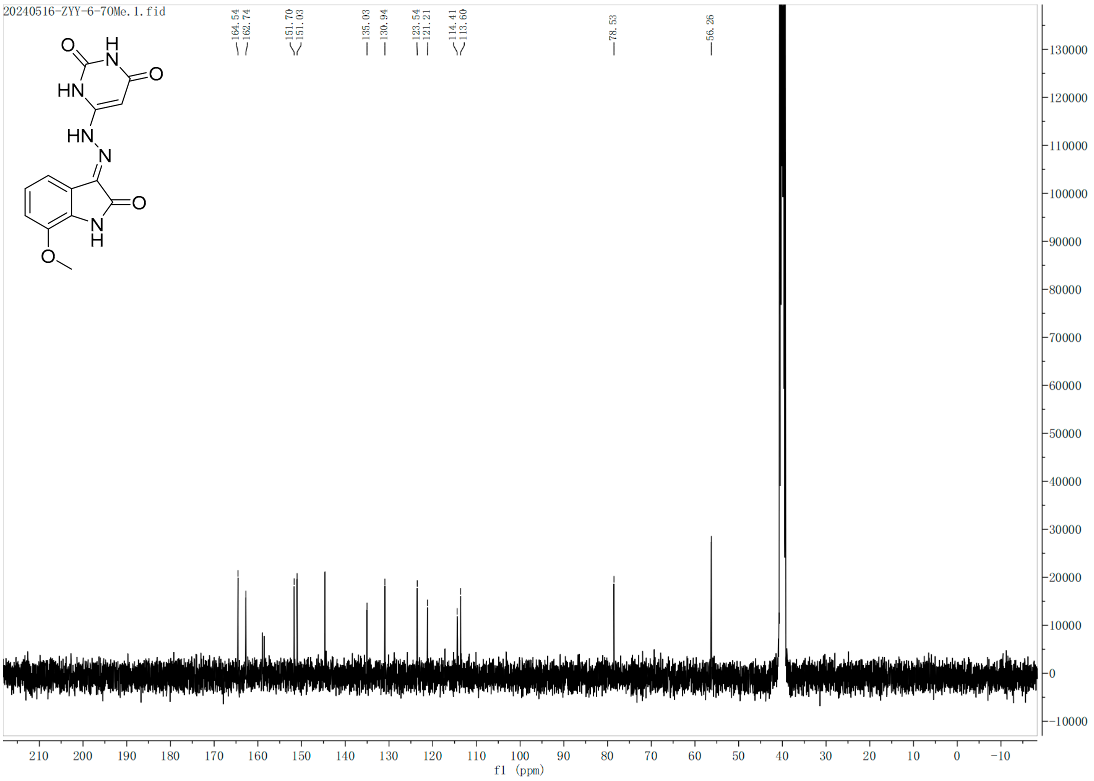

^{13}C NMR spectrum of **6r**.

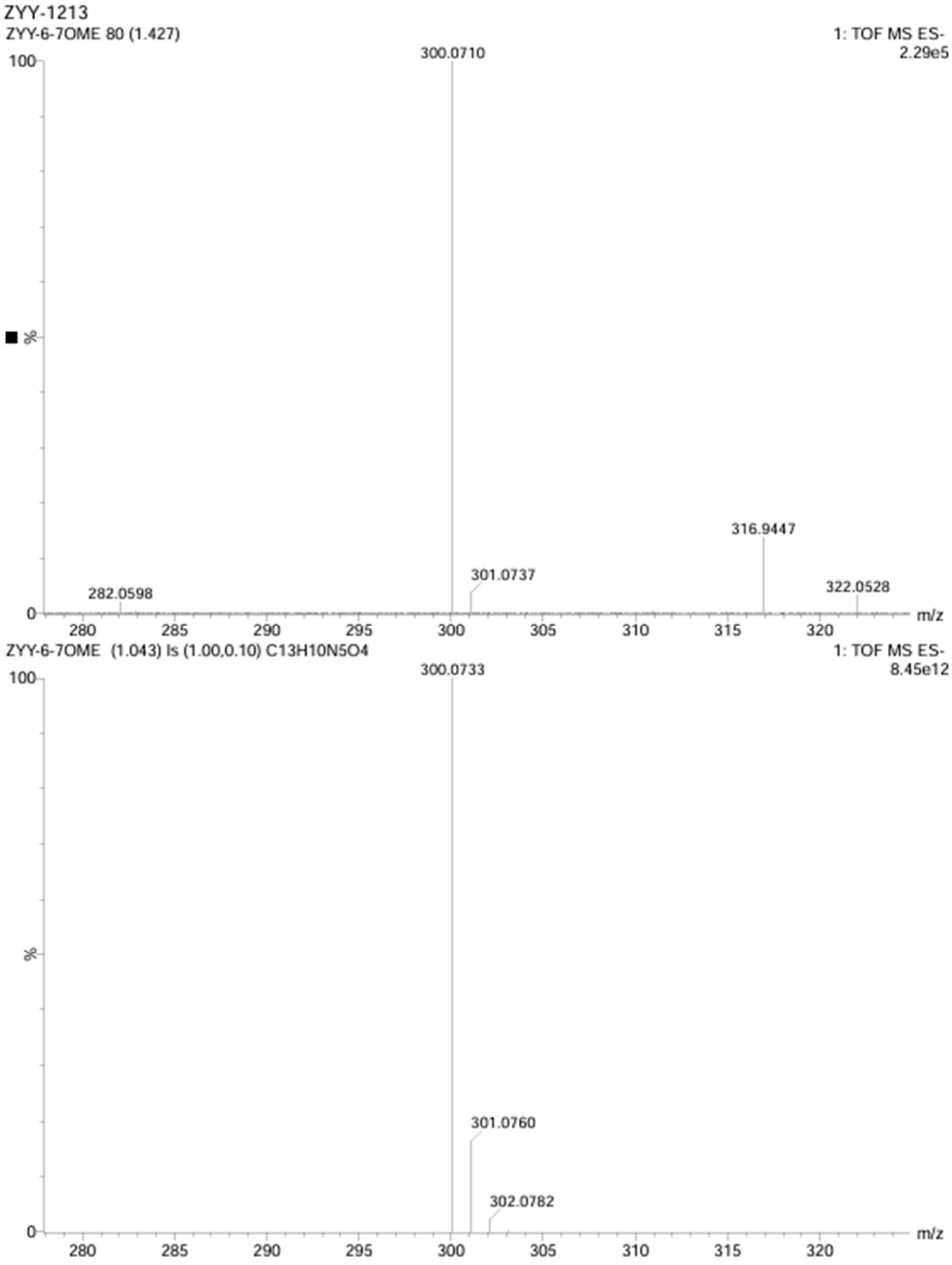

HRMS spectrum of **6r**.

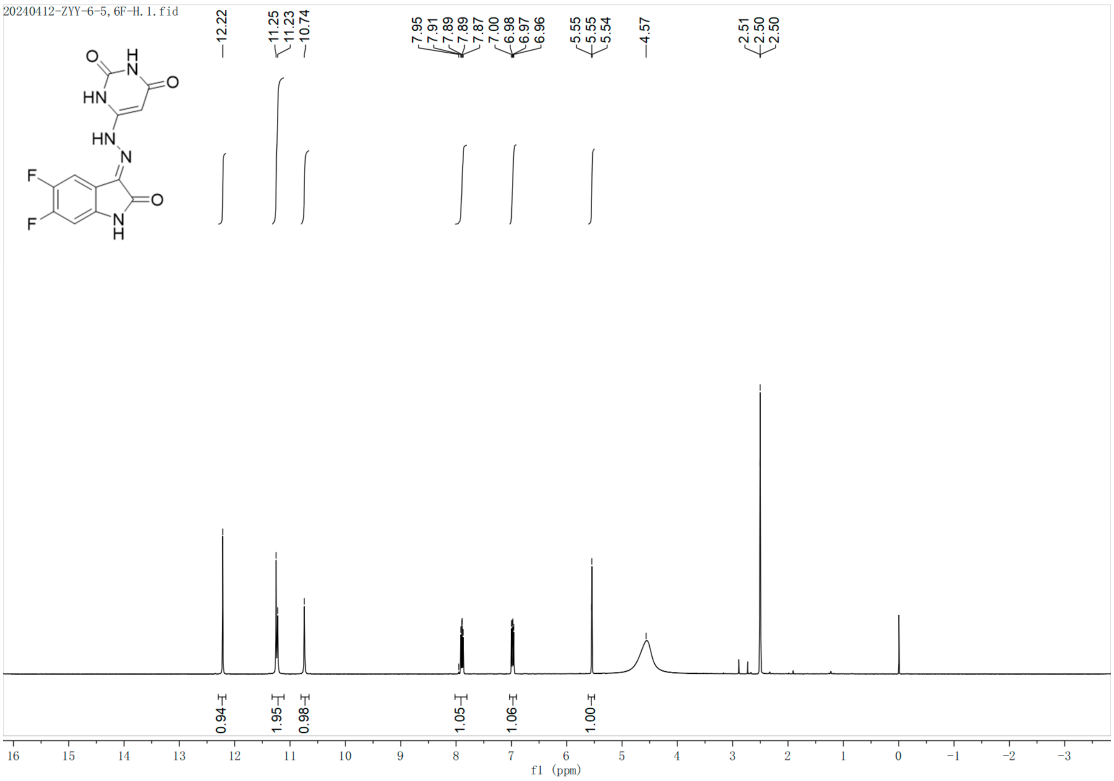

^{1}H NMR spectrum of **6s**.

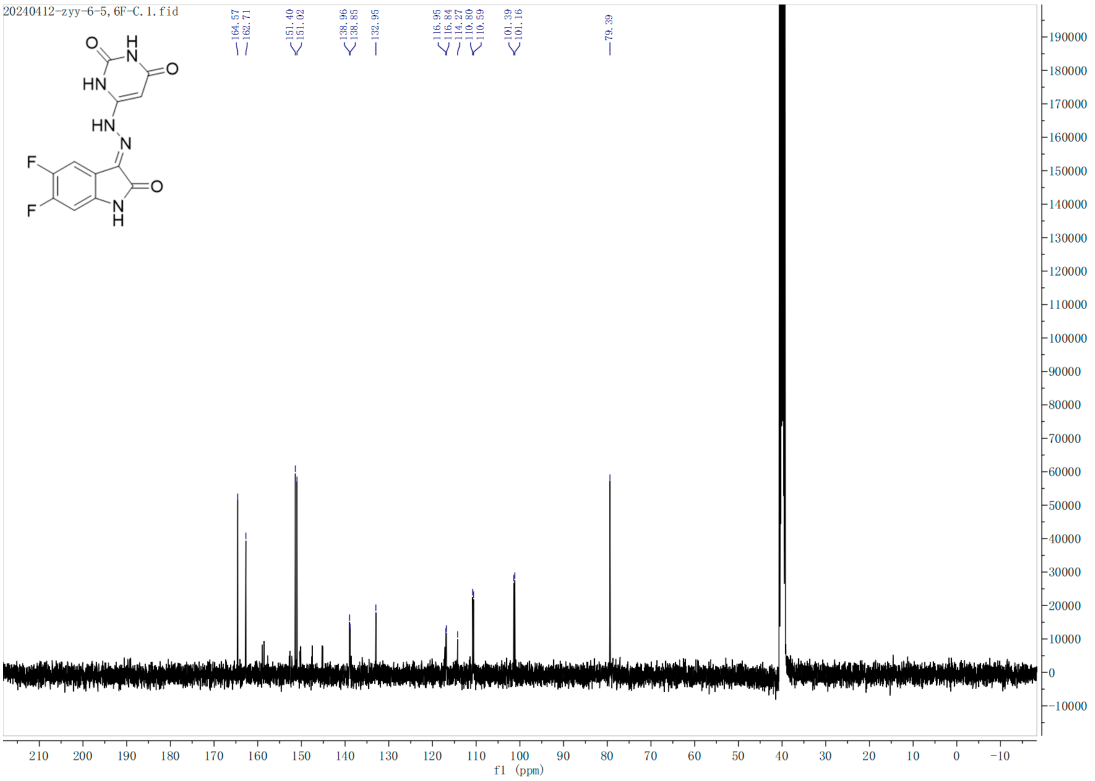

^{13}C NMR spectrum of **6s**.

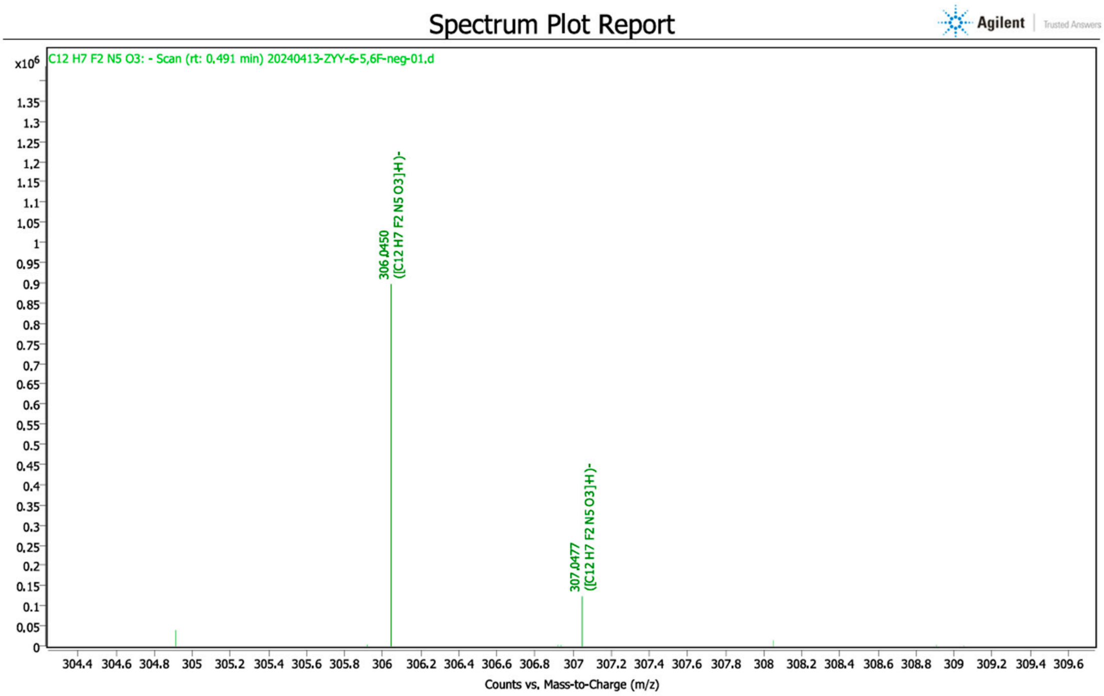

HRMS spectrum of **6s**.

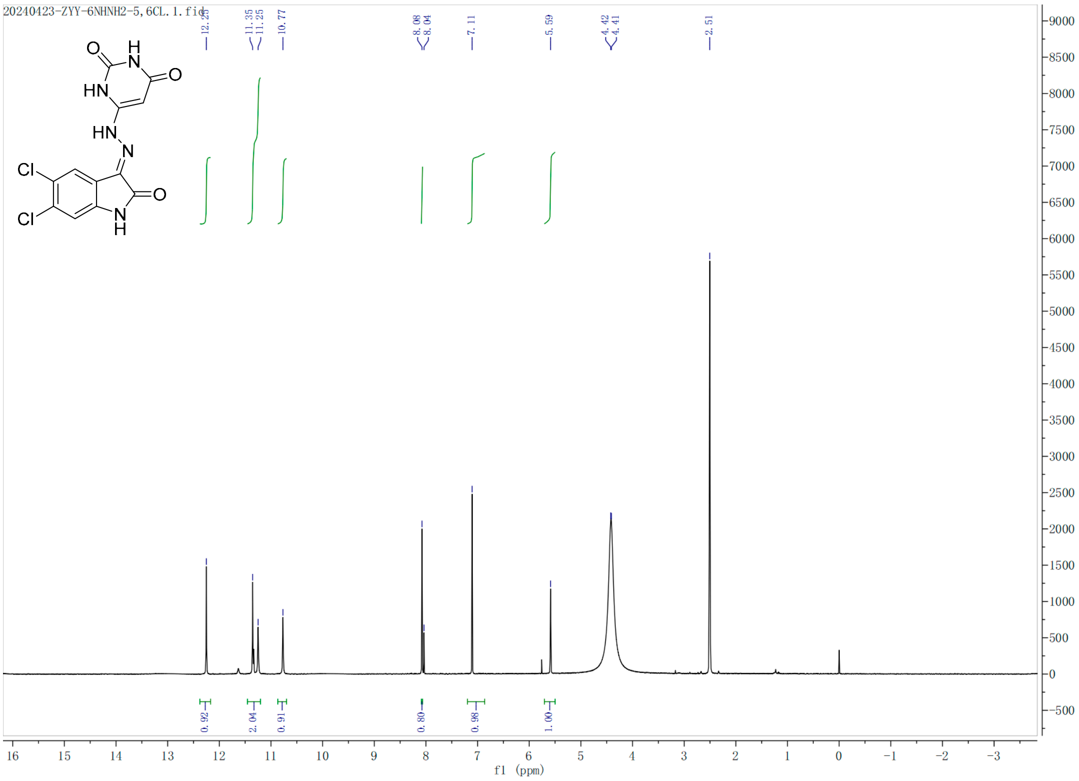

^{1}H NMR spectrum of **6t**.

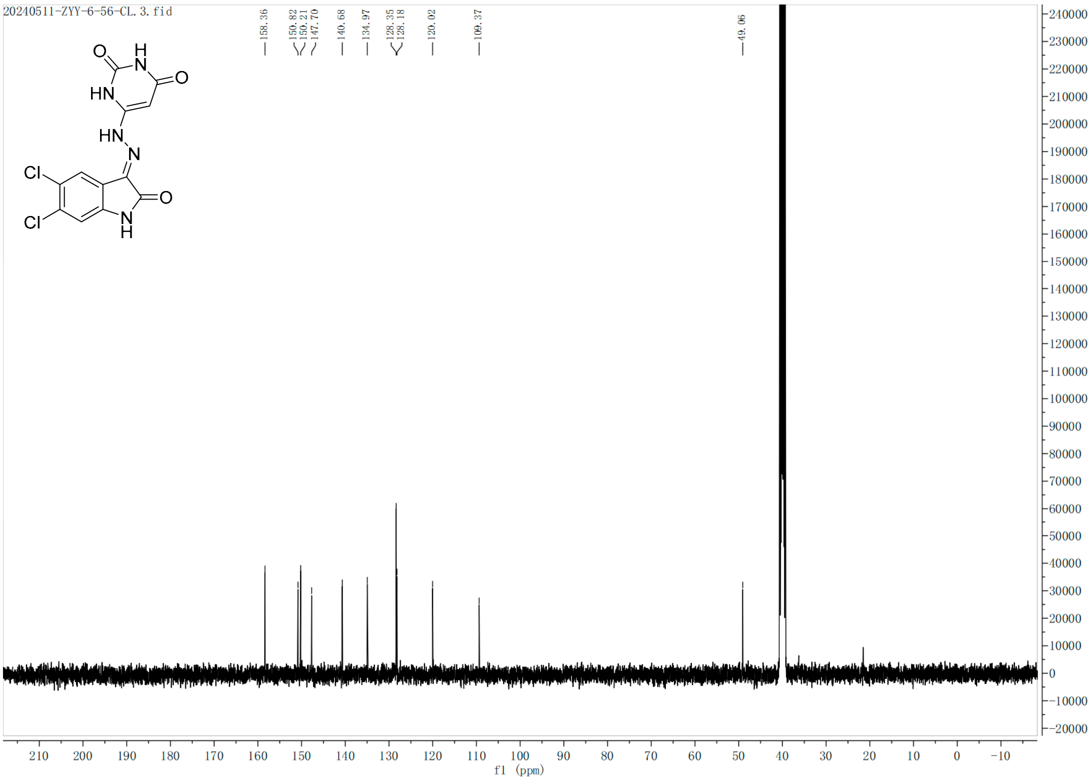

^{13}C NMR spectrum of **6t**.

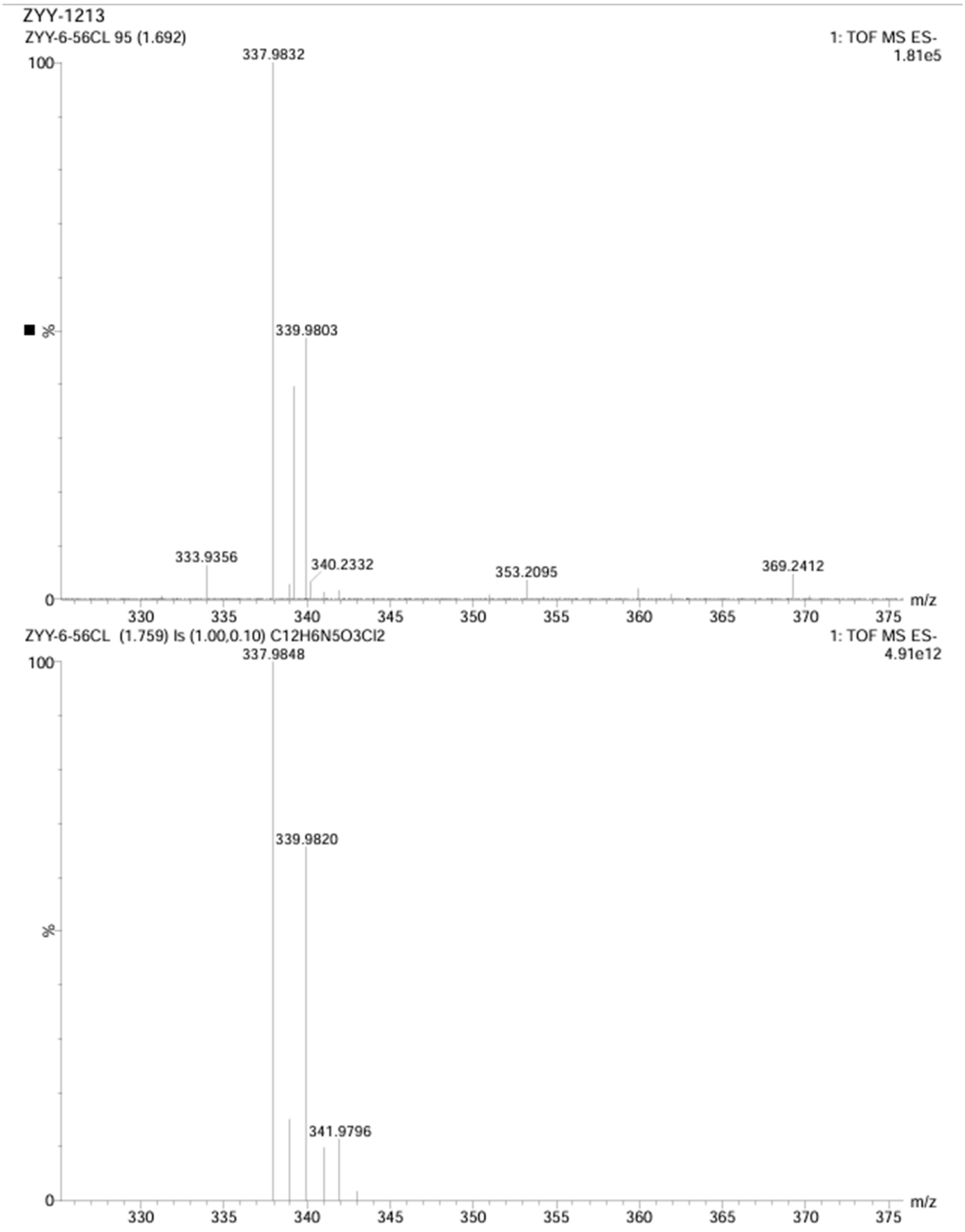

HRMS spectrum of **6t**.

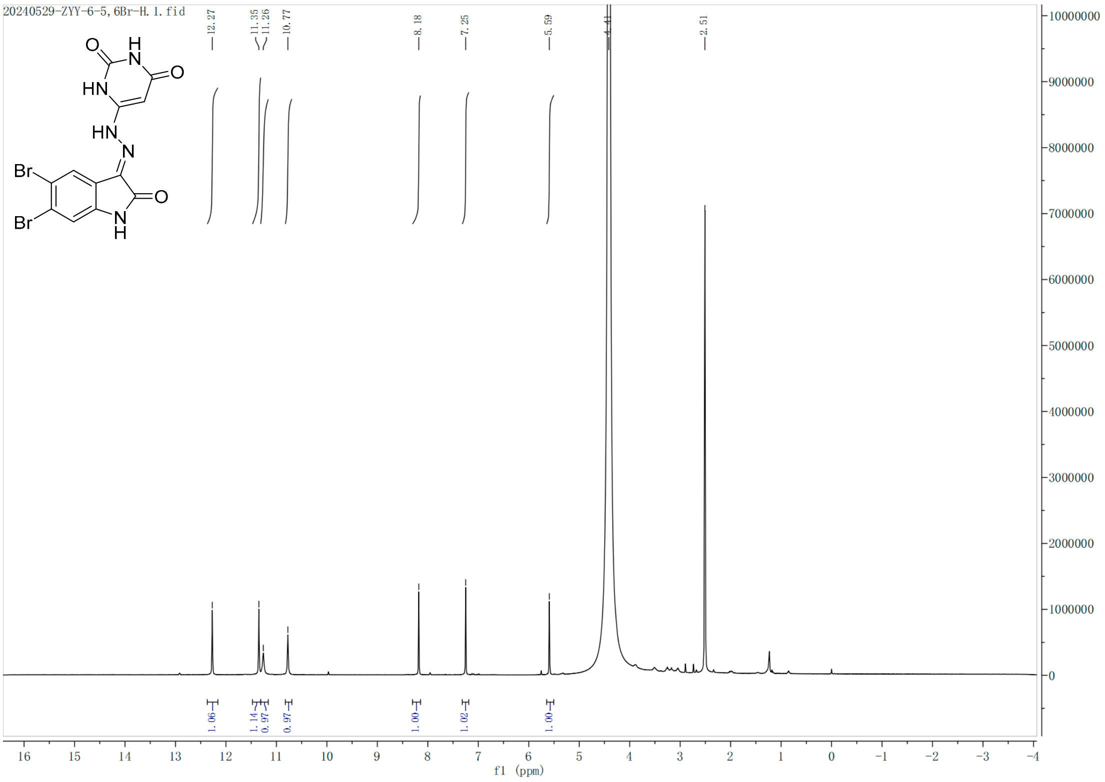

^{1}H NMR spectrum of **6u**.

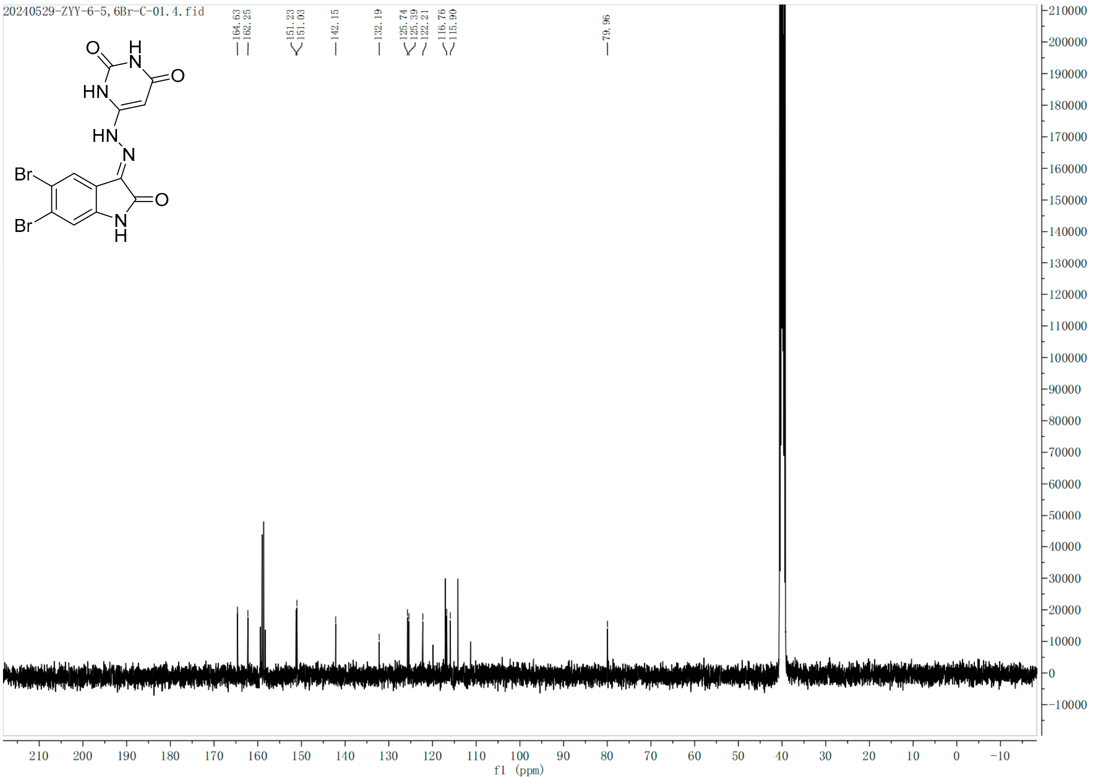

^{13}C NMR spectrum of **6u**.

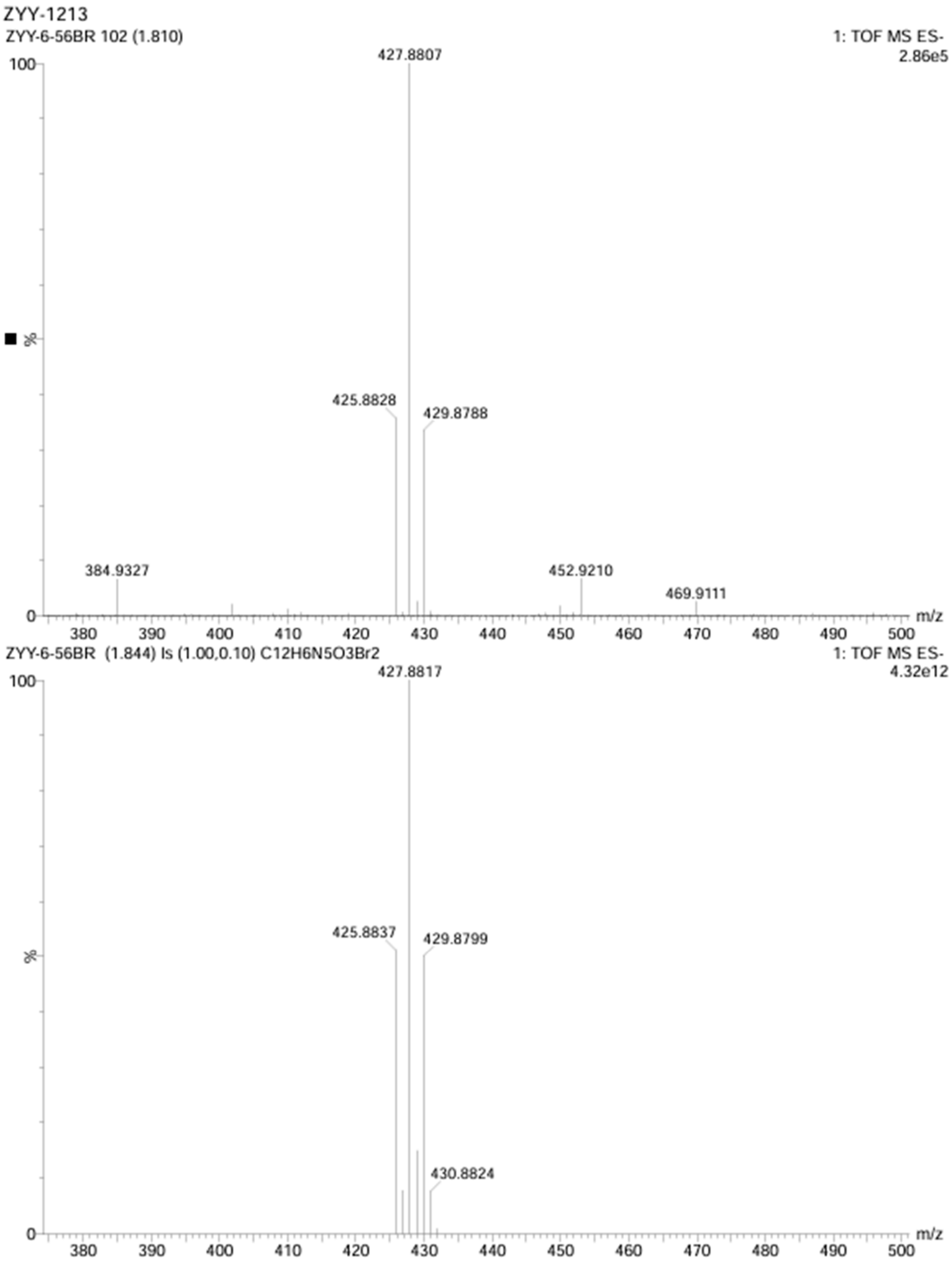

HRMS spectrum of **6u**.

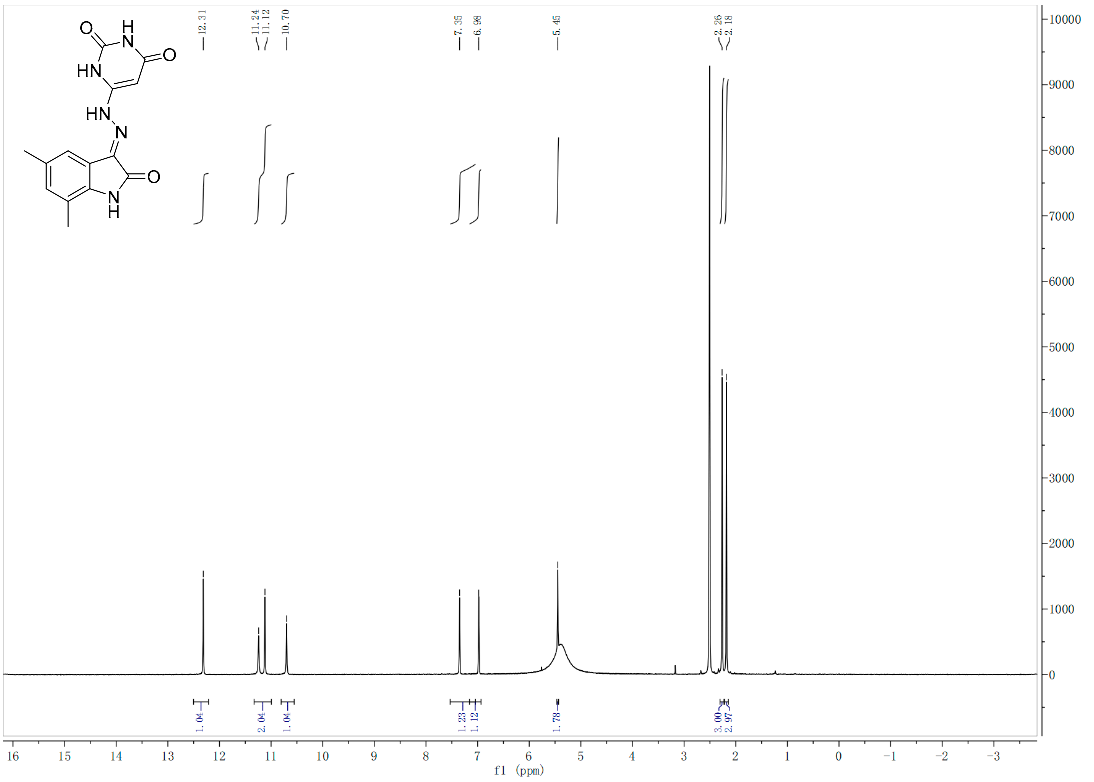

^{1}H NMR spectrum of **6v**.

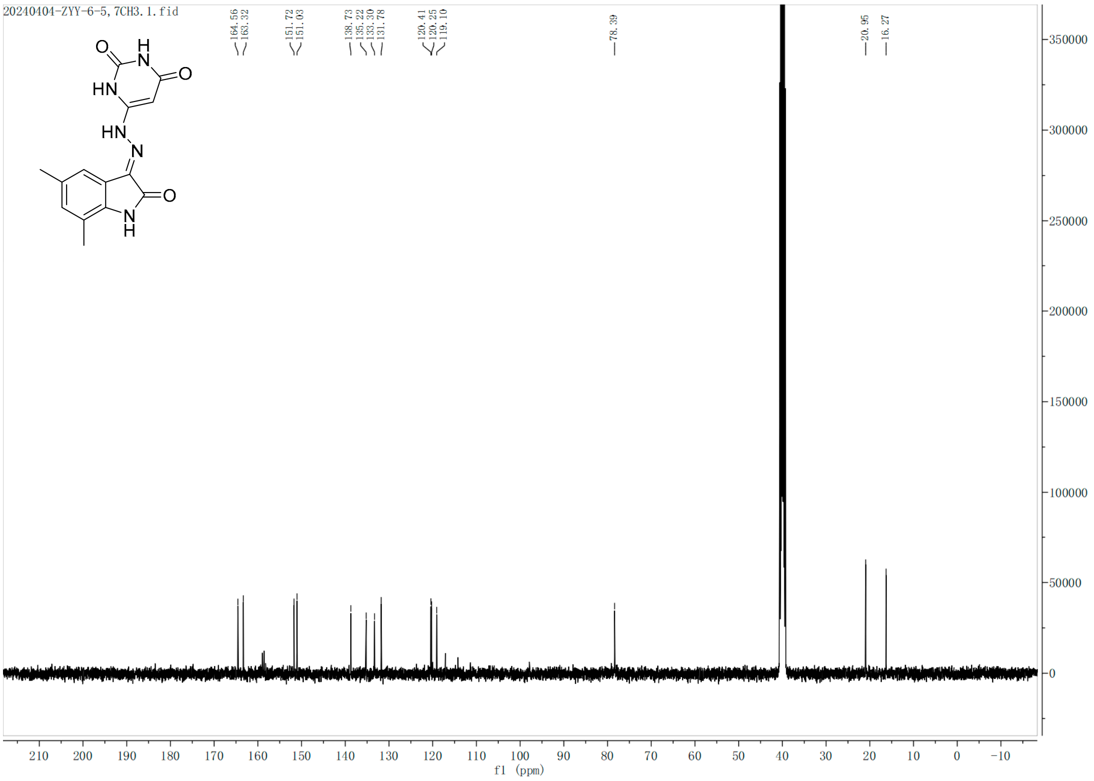

^{13}C NMR spectrum of **6v**.

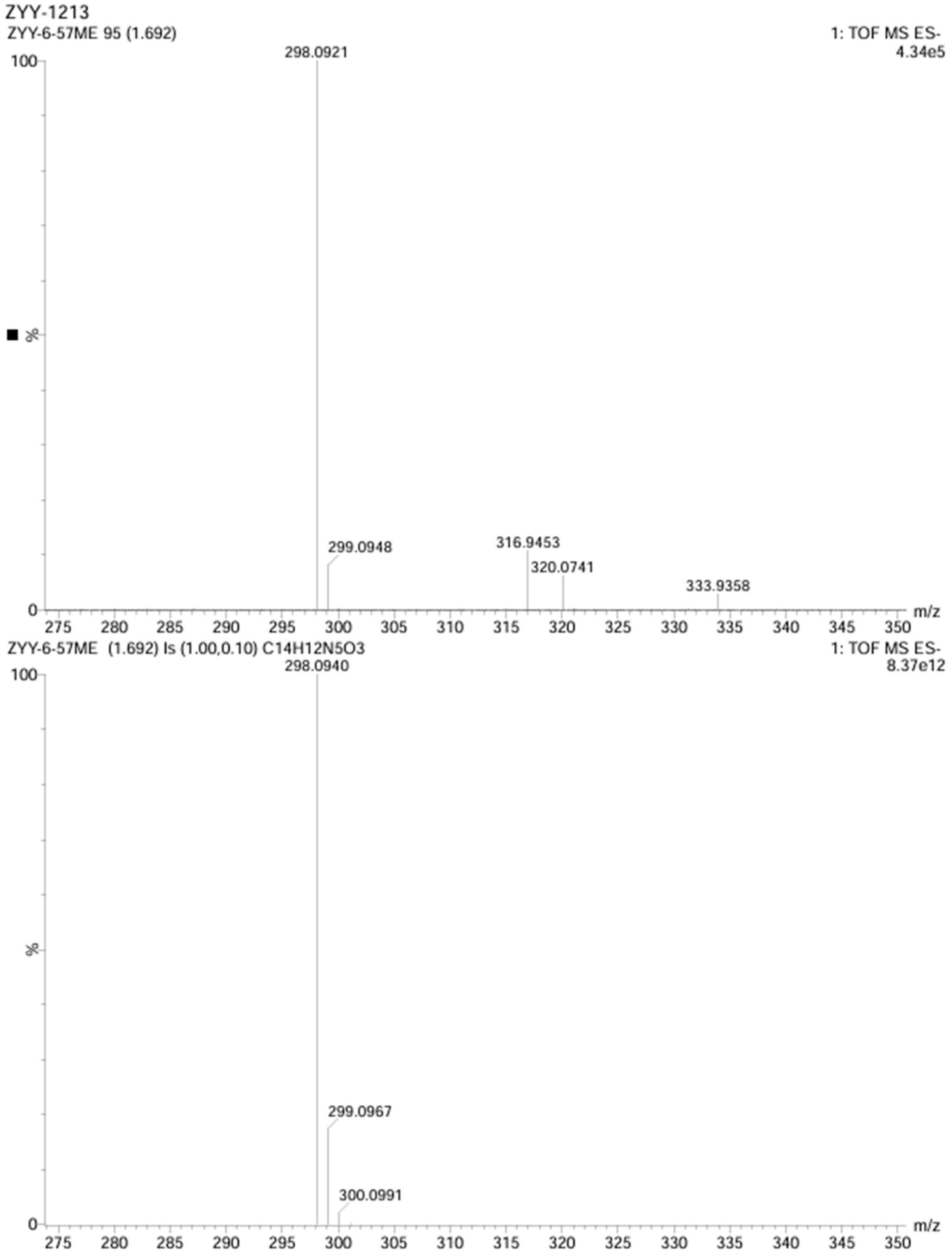

HRMS spectrum of **6v**.

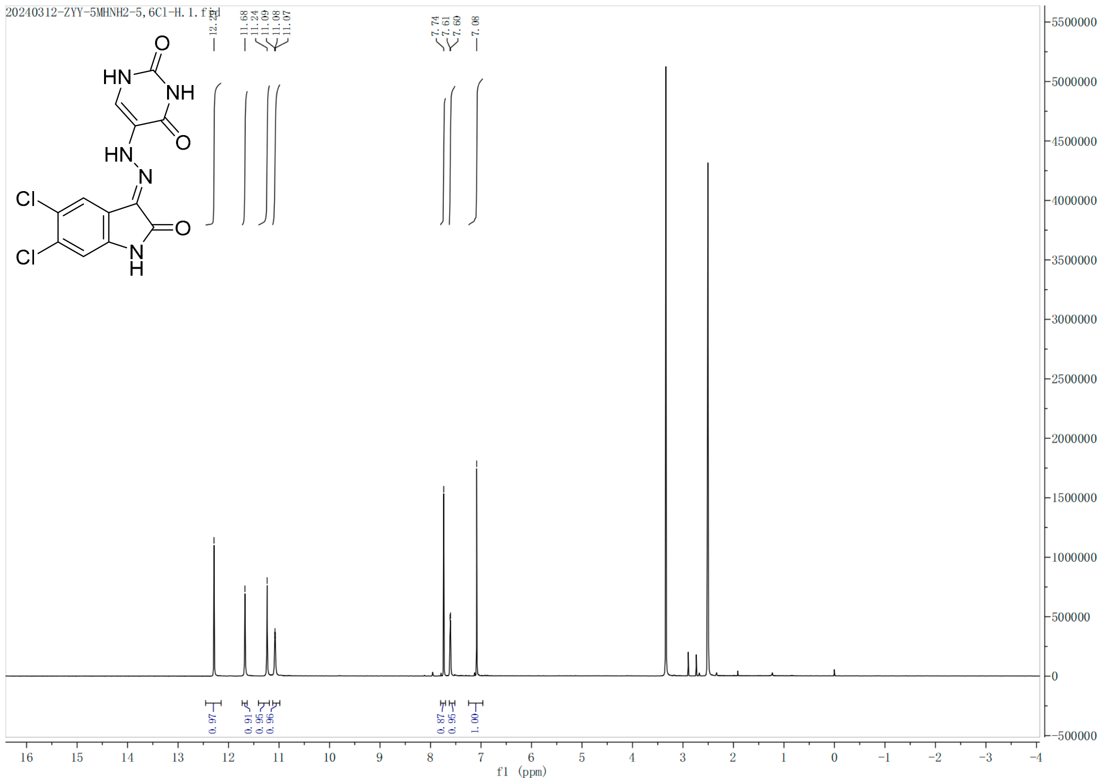

^{1}H NMR spectrum of **11a**.

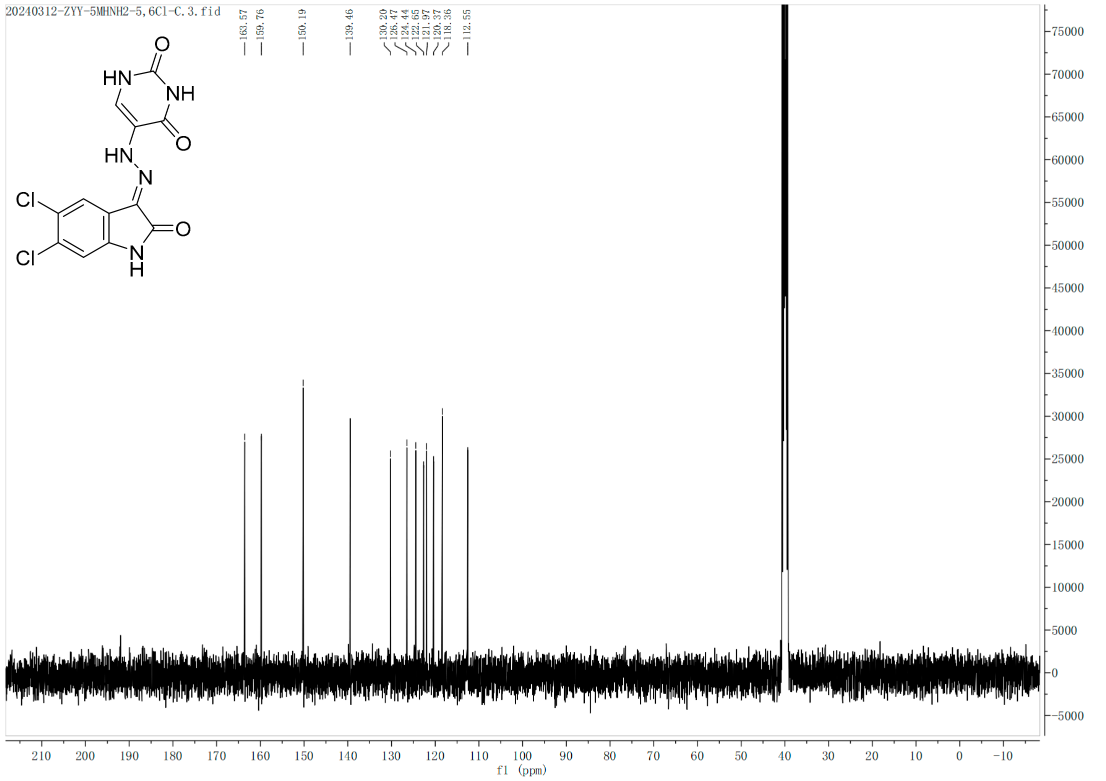

^{13}C NMR spectrum of **11a**.

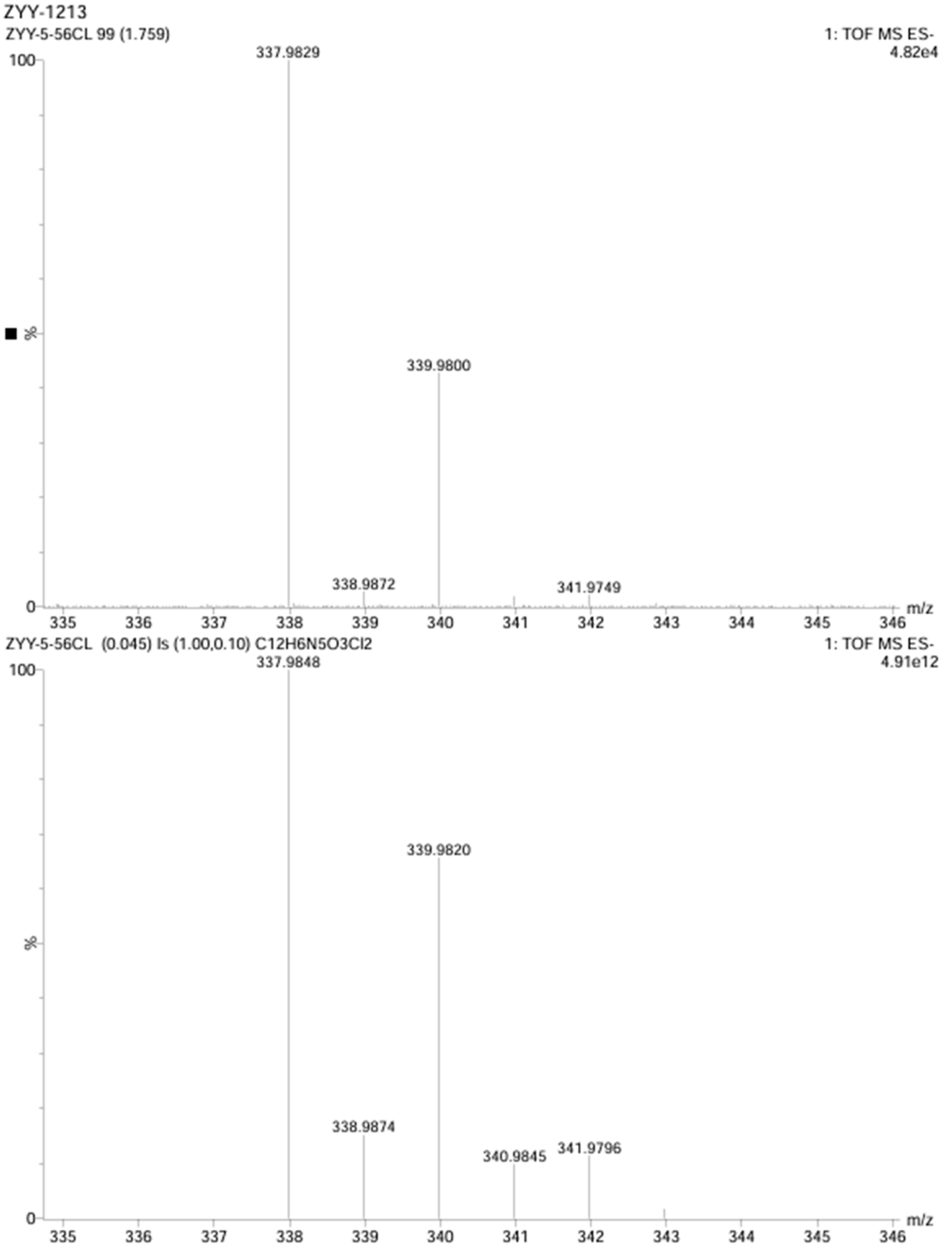

HRMS spectrum of **11a**.

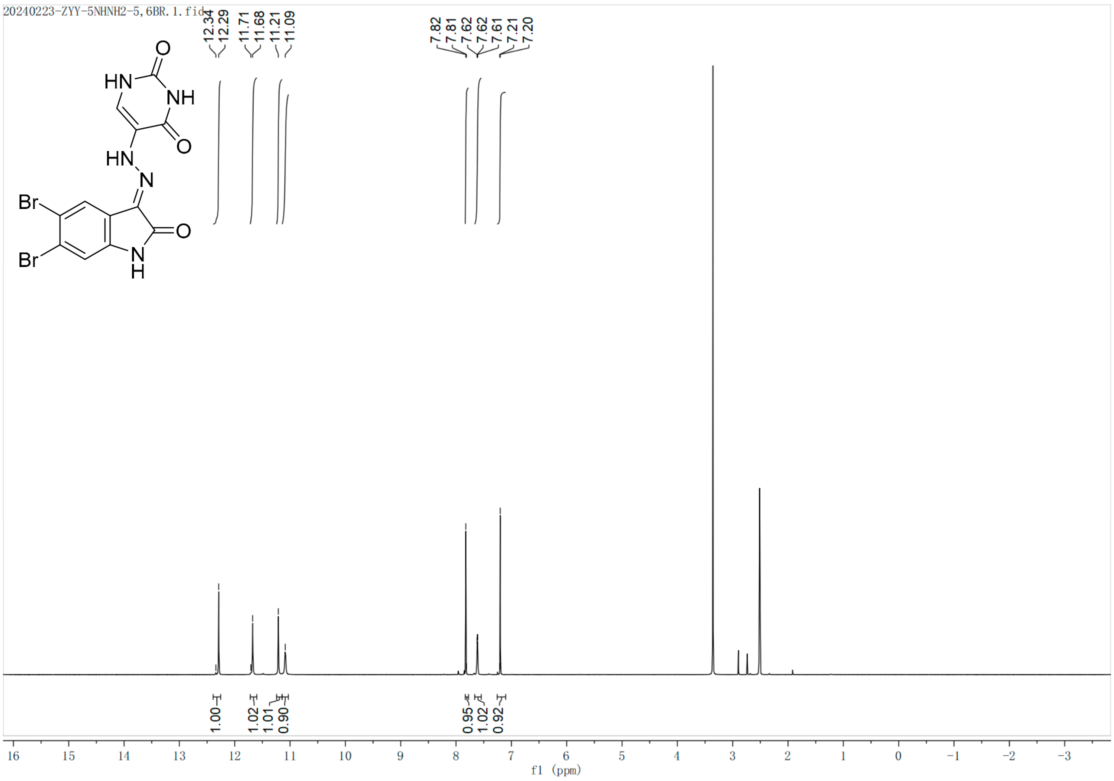

^{1}H NMR spectrum of **11b**.

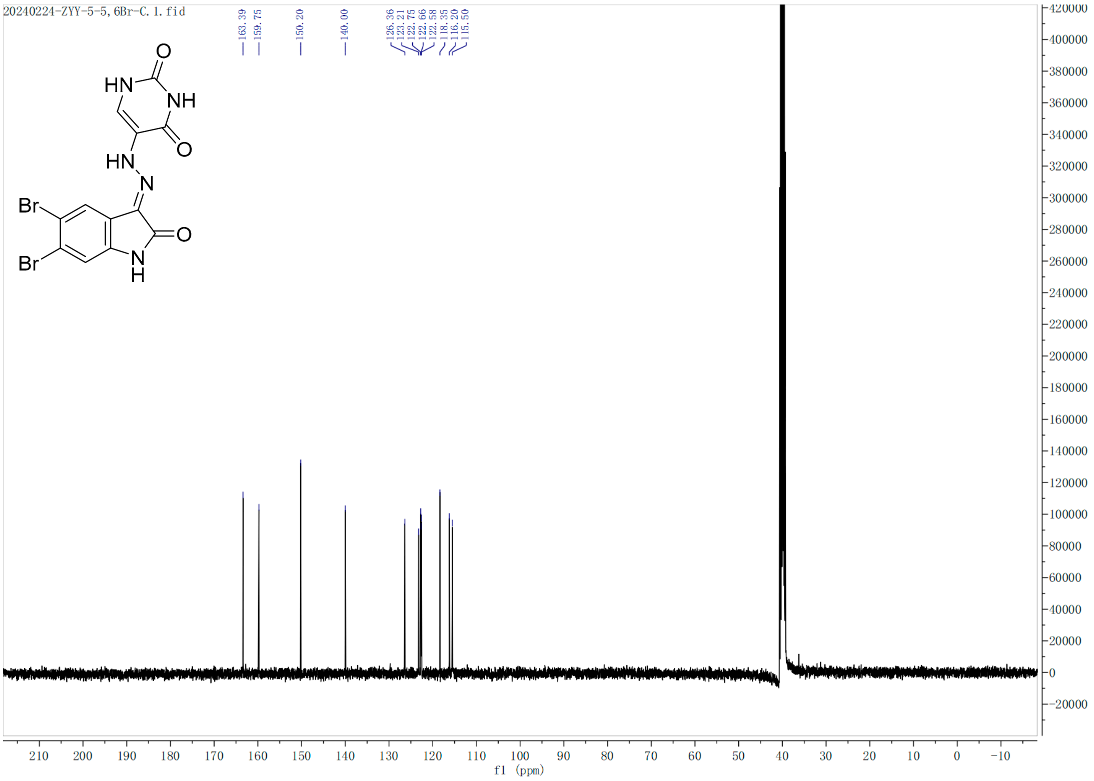

^{13}C NMR spectrum of **11b**.

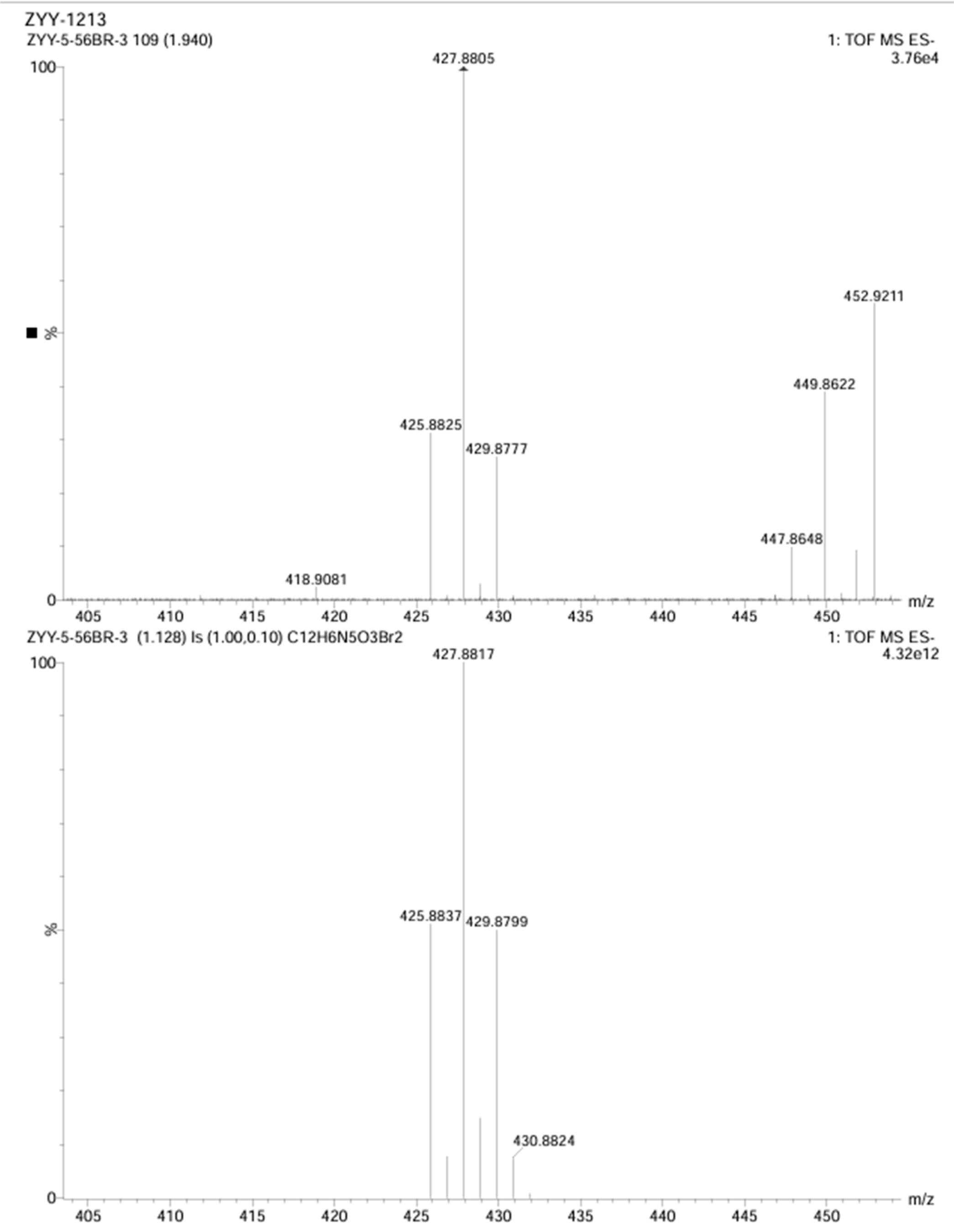

HRMS spectrum of **11b**.
